# Characterizing the HIV care continuum among children and adolescents with HIV in eastern and southern Africa in the era of “Universal Test and Treat”: A systematic review and meta‐analysis

**DOI:** 10.1002/jia2.26526

**Published:** 2025-06-13

**Authors:** Nel Jason L. Haw, Marcela Banegas, Sita Lujintanon, Lee Fairlie, Mutsa Bwakura‐Dangarembizi, Allison Agwu, Derek K. Ng, Catherine R. Lesko

**Affiliations:** ^1^ Department of Epidemiology Johns Hopkins Bloomberg School of Public Health Baltimore Maryland USA; ^2^ Wits RHI, Faculty of Health Sciences University of the Witwatersrand Johannesburg South Africa; ^3^ Department of Pediatrics and Child Health University of Zimbabwe College of Health Sciences Harare Zimbabwe; ^4^ Zvitambo Institute for Maternal and Child Health Research Harare Zimbabwe; ^5^ Division of Infectious Diseases, Department of Pediatrics Johns Hopkins Medicine Baltimore Maryland USA; ^6^ Division of Infectious Diseases, Department of Internal Medicine Johns Hopkins Medicine Baltimore Maryland USA

**Keywords:** adolescent, care cascade, child, eastern and southern Africa, HIV, youth adult

## Abstract

**Introduction:**

The “Universal Test and Treat” (UTT) era for antiretroviral therapy (ART) increased HIV service delivery to children and adolescents aged 0–19 with HIV. The goal is to reach ≥95% of people with HIV diagnosed, receiving ART and virally suppressed. We conducted a systematic review and meta‐analysis to describe the care continuum among children and adolescents with HIV during the UTT era in the UNAIDS eastern and southern African region.

**Methods:**

We searched PubMed, EMBASE and African Index Medicus databases for peer‐reviewed articles published from 1 January 2010 to 1 June 2023. We included studies reporting ≥1 care continuum proportion in ≥1 country in the study region during the country's UTT implementation. We extracted summary proportions and pooled them using random‐effects logistic regression.

**Results:**

Of 10,281 studies screened, 190 met the inclusion criteria. Studies came from 16 countries; many from South Africa (*n* = 37) and Ethiopia (*n* = 32). The meta‐analysis pooled proportions (95% confidence intervals) for children aged 0–14 were: 72% (60%, 81%) aware of HIV diagnosis; 95% (89%, 97%) on ART among diagnosed; 88% (76%, 95%) retained in HIV care after 12 months on ART; 77% (68%, 84%) self‐/caregiver‐reported ART adherence; 90% (79%, 95%) had a viral load test after ART initiation; and 76% (72%, 79%) viral suppression (<1000 copies/ml) while on ART with a viral load test. Similar proportions were estimated among adolescents aged 15–19: 73% (66%, 79%) diagnosed; 93% (92%, 94%) on ART; 80% (54%, 93%) retained; 74% (63%, 83%) adherent; 90% (79%, 95%) viral load test; and 78% (74%, 81%) viral suppression.

**Discussion:**

Estimates from this study on diagnosis, ART initiation and viral suppression were consistent with UNAIDS official estimates. Estimates on retention, adherence and viral suppression were similar to previous meta‐analyses conducted before UTT.

**Conclusions:**

Consistent with UTT expectations, most children and adolescents with HIV in eastern and southern Africa have initiated ART, but challenges remain on other care continuum indicators. Future planning for HIV programmes should consider locally informed, community‐supported approaches to consistently support children and adolescents with HIV throughout the HIV care continuum.

## 1 INTRODUCTION

In 2023, 2.4 million children and adolescents aged 0–19 were living with HIV globally. The majority (1.6 million, 62%) were living in eastern and southern Africa [1]. Between 2013 and 2023, the number of new HIV acquisitions within this age group in this region halved, from 270,000 to 130,000, and the number of AIDS‐related deaths reduced by two‐thirds, from 100,000 to 42,000 [1]. “Universal Test and Treat” (UTT), or initiating antiretroviral therapy (ART) after diagnosis regardless of CD4 count is one of the key policies responsible for reductions in HIV incidence [2] and mortality [3]. The World Health Organization (WHO) recommended “Test and Treat” to infants and children under 2 years of age in 2010, then expanded eligibility to children under 5 and pregnant and breastfeeding women (Option B+) in 2013, before finally expanding eligibility to all people with HIV in 2015 (“Treat All”) [4].

Even though most countries have introduced UTT, many children and adolescents with HIV are not fully engaged in HIV care. The Joint United Nations Programme on HIV/AIDS (UNAIDS) has set 95‐95‐95 targets with the goal that by 2025, 95% of people with HIV know their status, 95% who know their HIV status are on ART and 95% on ART are virally suppressed. These three indicators comprise part of the HIV care continuum, a model describing steps for people with HIV to achieve viral suppression. The HIV care continuum includes diagnosis, linkage to care (which include ART initiation), retention in HIV care, ART adherence and viral suppression [5]. Recent systematic reviews have estimated one or more indicators of the care continuum among children and adolescents with HIV primarily before UTT, such as retention in HIV care [6] and viral suppression [7] in low‐ and middle‐income countries, and adherence in sub‐Saharan Africa [8]. Other systematic reviews have covered years including both before and after UTT, but estimates were not differentiated after UTT [9, 10, 11, 12].

We aim to supplement current literature to inform potential interventions and strategies by providing a comprehensive description of the HIV care continuum for children and adolescents aged 0–19 with HIV during the UTT era in a region where majority of children and adolescents with HIV reside, eastern and southern Africa [13]. Describing the totality of the HIV care continuum across a major global region aids in the effective allocation of HIV global health resources, establishes benchmarks for progress and informs the development of regional HIV policy strategies.

## 2 METHODS

We conducted this systematic review in accordance with Conducting Systematic Reviews and Meta‐Analyses of Observational Studies of Etiology (COSMOS‐E) guidelines and reported results in accordance with the Preferred Reporting Items for Systematic Reviews and Meta‐Analyses (PRISMA) 2020 statement. This review is registered in Prospero (PROSPERO Registration number CRD42023467368).

### 2.1 Search strategy

We searched PubMed, EMBASE and the African Index Medicus databases for peer‐reviewed articles searchable using English keywords published from 1 January 2010 to 1 June 2023. We constructed search terms for children and adolescents with HIV, eastern and southern Africa, and each HIV care continuum indicator: diagnosis and awareness of HIV status, being on ART after diagnosis, retention in HIV care, ART adherence, and viral testing and suppression (Table S1).

### 2.2 Selection criteria

We included studies that: (1) covered ≥1 country in the UNAIDS eastern and southern African region; (2) reported on ≥1 proportion relating to ≥1 HIV care continuum indicator; (3) included study participants aged 19 and younger and reported proportions specific to that age group or overlaps with age 19 and younger with a limit of age 29, for example age 15–29 were included but not age 15–49; and (4) reported statistics during or overlapping with the country's UTT era (Table S2). More information on study selection regarding age range and calendar era is found in Table S2.

We excluded studies that simulated populations of children and adolescents with HIV as they are likely using model parameters from studies included in our screening. We included individual studies from systematic reviews and meta‐analyses that did not appear in our initial screening but excluded the pooled results from the meta‐analysis. We also excluded conference abstracts due to insufficient methodological detail to accurately extract care continuum indicators. We included studies that described the prevention of vertical transmission of HIV cascade only if they reported on any HIV care continuum indicator among pregnant women with HIV aged 19 years or younger during antenatal care or among infants who acquired HIV during postnatal care. There were no exclusions based on the sample size of the population of interest in the included studies.

### 2.3 Screening

Two authors screened titles and abstracts (NJLH and SL) and full‐text publications (NJLH and MB) independently using Covidence [14]. Studies were excluded if reviewers concurred, with any disputes resolved in consultation with a third reviewer as necessary (CRL or DKN). After the full‐text screening, we also hand‐searched the bibliography of the selected articles for any additional articles for inclusion.

### 2.4 Outcomes

We summarized the results across five indicators, described in Table 1, that reflect the WHO approach for describing the HIV care continuum [15]. These five indicators are: (1) proportion living with HIV who were diagnosed and aware of their HIV status, with awareness either on the level of the child/adolescent or on the primary caregiver; (2) proportion diagnosed with HIV who are on ART sometime after diagnosis; (3) proportion on ART who were retained in HIV care; (4) proportion on ART who were adherent, as ascertained by either self/caregiver report or health facility reports (e.g. pill count, frequency of pharmacy refills); and (5) proportion on ART that achieved viral suppression, disaggregated between: (5a) proportion on ART with a viral load test; and (5b) proportion with a viral load test that achieved viral suppression.

**Table 1:** HIV care continuum indicators included in the meta‐analysis

	Indicator	Numerator	Denominator	Remarks	No. (%) of studies reporting on care continuum indicator
Diagnosis and awareness of HIV status	(1) Proportion of children and adolescents with HIV who were diagnosed or aware of their HIV status (first 95‐95‐95 target)	Children or adolescents who were known to have been previously diagnosed or reported a positive diagnosis either by them or by their caregiver prior to the testing conducted during the study	Children or adolescents who were tested positive during the study, regardless of previous knowledge of HIV diagnosis	Studies that looked at HIV status disclosure from caregiver to child/adolescent were not included, as this does not represent a gap that prevents them from proceeding with the rest of the HIV care continuum	19 (10%)
Being on antiretroviral therapy (ART) after diagnosis	(2) Proportion of children and adolescents diagnosed with HIV who are on ART sometime after diagnosis	Children or adolescents who have either reported being on ART by them or their caregiver, have a record of ART use on their health record or reported by their health provider, or have had a laboratory test detecting ART during the study	Children or adolescents who are living with HIV whose status is aware to them or their caregiver	Additionally, in the meta‐analysis, the proportion of children and adolescents diagnosed with HIV who rapidly initiated ART (<7 days) were also pooled	54 (28%)
Retention in HIV care	(3) Proportion of children and adolescents having initiated ART who continuously attended clinic visits	Children or adolescents who have continuously attended clinic visits by a specified time from ART initiation; if the study primarily measured loss to follow up based on non‐attendance within a specified time window, this was taken to be the complement of loss to follow up, with deaths and transfers excluded	Children or adolescents who have initiated ART during study follow‐up	In the meta‐analysis, proportions for pooled for retention at 6, 12 and 24 months	53 (28%)
Adherence to ART	(4) Proportion of children and adolescents on ART who were adherent	Children or adolescents who were determined to have good adherence, based on the study's definition of adherence (e.g. pill counts, validated self‐rated adherence survey questions)	Children or adolescents who were on ART at the beginning or sometime during study follow‐up	In the meta‐analysis, proportions were pooled among studies with self‐ or caregiver‐reported adherence, and among studies with health facility‐reported adherence (i.e. either retrieved from health records or reported by health provider)	47 (25%)
Viral suppression	(5a) Proportion of children and adolescents on ART with a viral load test	Children or adolescents who have a recorded viral load test after ART initiation	Children or adolescents who were on ART	The third 95‐95‐95 target is the proportion of children and adolescents on ART who were virally suppressed	110 (58%)
	(5b) Proportion of children and adolescents on ART and have a viral load test who were virally suppressed	Children or adolescents who achieved viral suppression while on ART	Children or adolescents who were on ART and have a viral load result	In the meta‐analysis, proportions were pooled according to a viral load cutoff of 1000 copies/ml. Studies that used lower cutoffs had appropriate data transformations applied.	

### 2.5 Data extraction

We developed a standardized data extraction database using AirTable, a cloud database, which was piloted by five reviewers (NJLH, SL, MB, CRL, DKN) and revised. Data extraction of summary measures from published studies was conducted by one reviewer (NJLH) and double‐checked by another (MB), with any disagreements resolved by discussion.

Data extraction was done at the study level and HIV care continuum indicator level. For the study level, we extracted: author name, publication year, country, study year/s, study design (cross‐sectional/longitudinal, study setting (health centre/hospital/community), type of residence (urban/rural/both), follow‐up months, age range, sample size and quality assessment. We also indicated if the study came from a specific cohort study, surveillance system or electronic health record system.

For each HIV care continuum indicator in each study, we extracted its definition, numerator unit (e.g. people, tests) and value, denominator unit and value, proportion, calendar years of study, country, health facility setting (health centre/hospital/community), type of residence (urban/rural), study design (cross‐sectional/longitudinal), age and sex at birth. For all HIV care continuum indicators, we extracted the summary study measures, and wherever possible, age‐, sex‐, calendar year‐, country‐specific measures in the main paper and supplement.

For ART initiation, we extracted the time from diagnosis when the proportion on ART was assessed (e.g. initiated ART within 7 days). For retention in HIV care, we extracted the definition of loss to follow‐up (e.g. 3‐month gap from the last HIV clinic visit), and the month from ART initiation when retention was assessed (e.g. 12 months after ART initiation). For ART adherence, we extracted the definition and source of adherence measure (self‐ or caregiver or health provider report) and the month of recall (e.g. 1 month before an interview). For viral suppression, we extracted the month from ART initiation when the viral load test was conducted (e.g. 5 months after ART initiation), and the cutoff used to determine viral suppression (e.g. 1000 copies/ml).

### 2.6 Quality assessment

We performed a quality assessment using the Joanna‐Briggs Institute Critical Appraisal Tool for prevalence and incidence [16], a 9‐item checklist. We reported on the most common types of bias across the included studies.

### 2.7 Data synthesis

We summarized study characteristics and the HIV care continuum indicators in tables. We conducted meta‐analyses to pool proportions among HIV care continuum definitions consistently used in UNAIDS and WHO monitoring and evaluation indicators (Table 1) [17]. We stratified meta‐analysis results by age group, that is children aged < 15 and adolescents aged 15–19.

To pool proportions, we used a random intercept logistic regression model, where the numerator is directly modelled as a binomial distribution with the denominator (sample size) and the logit‐transformed proportion as parameters [18]. We used some data transformations to recover the number of events for survey‐weighted estimates [19], and to standardize the calculation of viral suppression proportions to a common threshold of 1000 copies/ml [20]. Studies that include multiple countries were disaggregated by country whenever possible, so each unit of analysis in the meta‐analysis represents one study country.

The summary of each study estimate in the meta‐analysis was presented graphically using forest plots, stratified by age group (children aged 0–14 and adolescents aged 15–19), with confidence intervals (CIs) recalculated using the Clopper−Pearson approach [21]. We did not conduct statistical tests for heterogeneity, as we expect that proportions differ across study contexts, thus heterogeneity measures do not have a meaningful interpretation [22]. The pooled proportions were interpreted as the median proportion across all these studies [23]. CIs for pooled proportions were calculated with Knapp−Hartung adjustments [24]. We also did not conduct statistical tests for publication bias as the assumptions underlying these tests are not appropriate in the meta‐analyses of proportions [25]. The meta‐analysis was conducted in R 4.3.2 (R Foundation) using the *meta* package.

### 2.8 Role of the funding source

The funders of the study did not play a role in the study design, data collection, data analysis, data interpretation or report writing.

## 3 RESULTS

**Figure 1:**
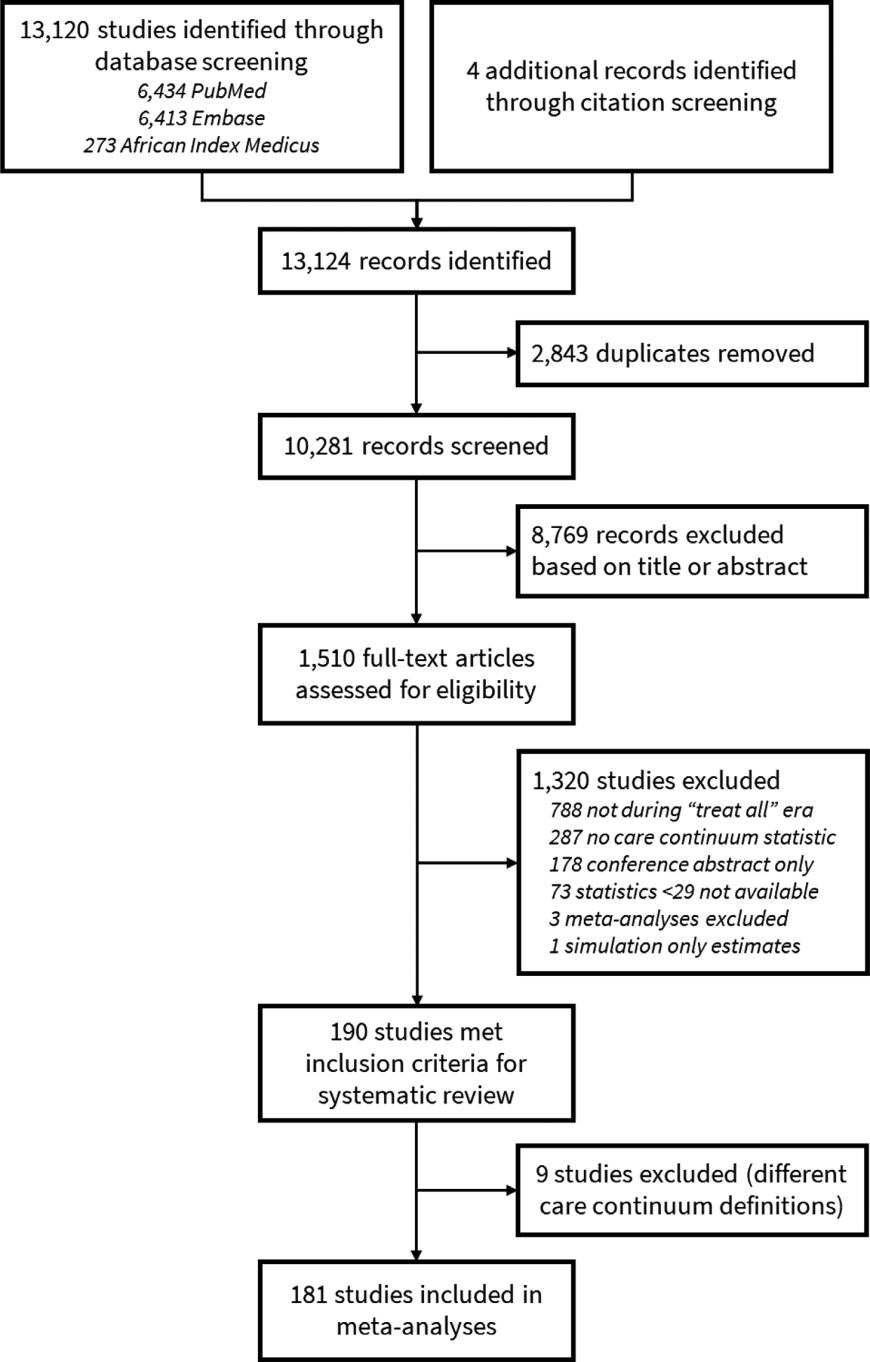
**PRISMA flow diagram of the systematic review and meta‐analysis**.

We identified 10,281 unique records, of which 1510 were examined for full‐text eligibility, and 190 articles were eligible for inclusion in the systematic review, representing data from 647,904 children and adolescents with HIV (Figure 1). The median sample size of children and adolescents with HIV across all studies was 377. Of these studies, 19 (10%) reported on diagnosis and awareness of HIV status [26, 27, 28, 29, 30, 31, 32, 33, 34, 35, 36, 37, 38, 39, 40, 41, 42, 43, 44], 54 (28%) reported being on ART after diagnosis [26, 27, 28, 29, 30, 31, 32, 33, 34, 35, 36, 38, 39, 40, 42, 43, 44, 45, 46, 47, 48, 49, 50, 51, 52, 53, 54, 55, 56, 57, 58, 59, 60, 61, 62, 63, 64, 65, 66, 67, 68, 69, 70, 71, 72, 73, 74, 75, 76, 77, 78, 79, 80, 81], 53 (28%) reported on retention in HIV care [41, 46, 50, 56, 66, 74, 76, 78, 79, 82, 83, 84, 85, 86, 87, 88, 89, 90, 91, 92, 93, 94, 95, 96, 97, 98, 99, 100, 101, 102, 103, 104, 105, 106, 107, 108, 109, 110, 111, 112, 113, 114, 115, 116, 117, 118, 119, 120, 121, 122, 123, 124, 125], 47 (25%) reported on ART adherence [10, 11, 83, 86, 87, 90, 101, 106, 107, 108, 111, 126, 127, 128, 129, 130, 131, 132, 133, 134, 135, 136, 137, 138, 139, 140, 141, 142, 143, 144, 145, 146, 147, 148, 149, 150, 151, 152, 153, 154, 155, 156, 157, 158, 159, 160, 161] and 111 (58%) reported on viral suppression [10, 26, 27, 28, 29, 30, 31, 33, 34, 35, 36, 38, 39, 40, 42, 44, 46, 49, 50, 66, 74, 76, 78, 79, 85, 87, 96, 97, 100, 105, 106, 109, 111, 117, 122, 123, 124, 126, 128, 129, 130, 131, 134, 136, 138, 139, 140, 143, 144, 146, 148, 150, 152, 154, 155, 158, 160, 162, 163, 164, 165, 166, 167, 168, 169, 170, 171, 172, 173, 174, 175, 176, 177, 178, 179, 180, 181, 182, 183, 184, 185, 186, 187, 188, 189, 190, 191, 192, 193, 194, 195, 196, 197, 198, 199, 200, 201, 202, 203, 204, 205, 206, 207, 208, 209, 210, 211, 212, 213, 214]. For the meta‐analysis, 181 studies were included.

**Figure 2:**
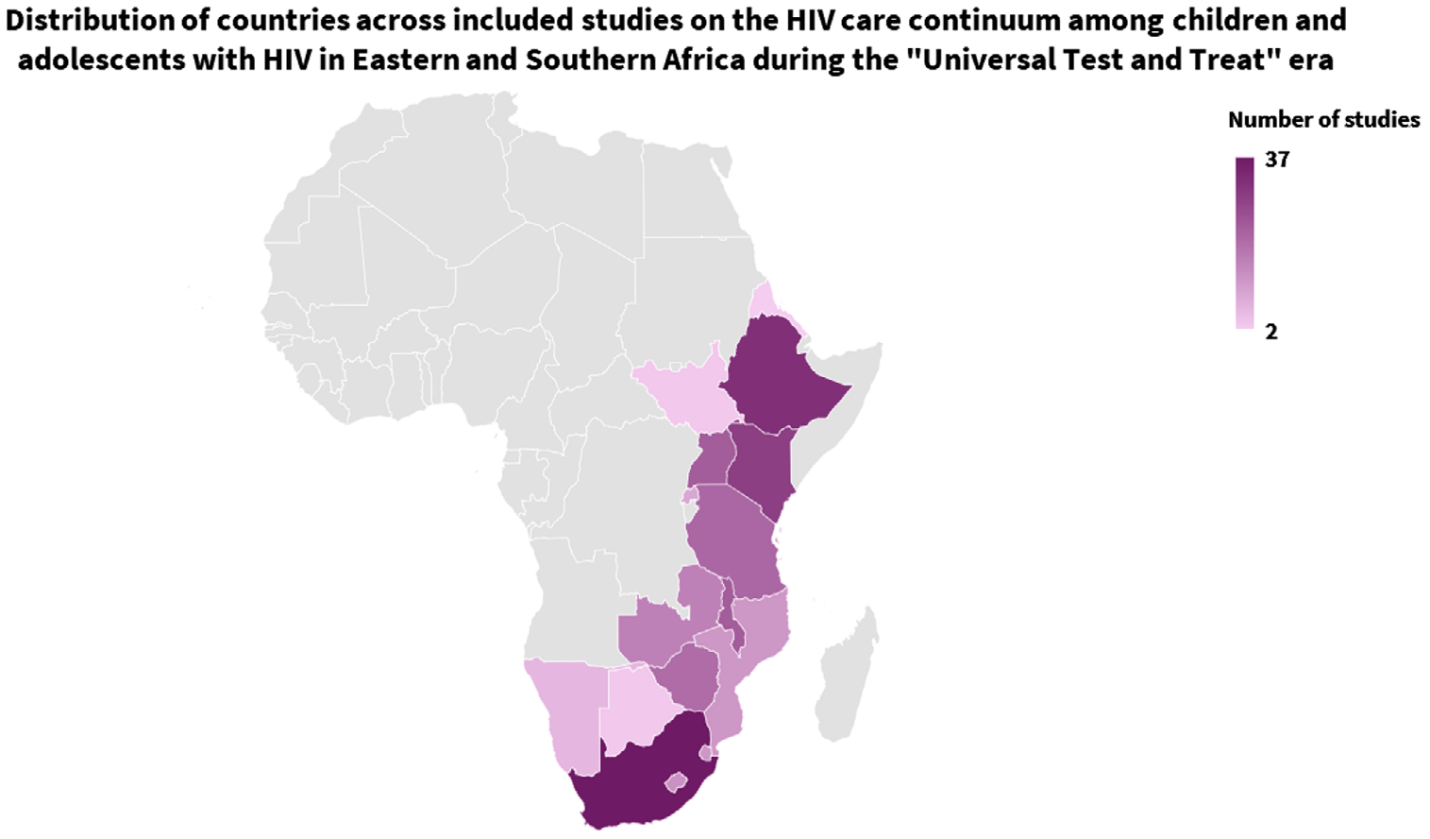
**Distribution of countries included in the systematic review (*n* = 190 studies)**.

The studies altogether represented 16 countries in eastern and southern Africa (Figure 2 and Table 2; Table S3 describes each study). Countries with the greatest number of studies were South Africa (37, 19%), Ethiopia (32, 17%) and Kenya (29, 15%). Two‐thirds (124, 65%) of the included studies were from longitudinal studies, while the rest were cross‐sectional studies. In the risk of bias assessment, the most common bias was the lack of representativeness of the study population to the target population (172, 90%), with most studies (110, 57%) employed a census of all available health records, and these health facility censuses were not necessarily representative of the target population with HIV the health facilities serve.

**Table 2:** Descriptives of the included studies in the systematic review

	Overall	Diagnosis and awareness of HIV status	Being on antiretroviral therapy (ART) after diagnosis	Retention in HIV care	Adherence to ART	Viral suppression
**Total number of studies**	190	19	54	53	47	110
**Country**						
South Africa	37 (19%)	7 (37%)	11 (20%)	12 (23%)	5 (11%)	26 (24%)
Ethiopia	32 (17%)	1 (5%)	3 (6%)	8 (15%)	16 (34%)	16 (15%)
Kenya	29 (15%)	2 (11%)	10 (19%)	5 (9%)	4 (9%)	19 (17%)
Malawi	23 (12%)	6 (32%)	9 (17%)	5 (9%)	3 (6%)	17 (15%)
Uganda	23 (12%)	2 (11%)	7 (13%)	4 (8%)	5 (11%)	12 (11%)
Tanzania	21 (11%)	2 (11%)	6 (11%)	4 (8%)	7 (15%)	11 (10%)
Zimbabwe	20 (11%)	3 (16%)	9 (17%)	5 (9%)	1 (2%)	11 (10%)
Zambia	16 (8%)	4 (21%)	10 (19%)	3 (6%)	··	7 (6%)
Lesotho	13 (7%)	4 (21%)	6 (11%)	3 (6%)	1 (2%)	9 (8%)
Eswatini	12 (6%)	3 (16%)	6 (11%)	2 (4%)	··	7 (6%)
Mozambique	12 (6%)	··	5 (9%)	5 (9%)	1 (2%)	2 (2%)
Rwanda	9 (5%)	··	5 (9%)	6 (11%)	1 (2%)	3 (3%)
Namibia	6 (3%)	1 (5%)	1 (2%)	2 (4%)	1 (2%)	4 (4%)
Botswana	3 (2%)	··	2 (4%)	1 (2%)	··	3 (3%)
South Sudan	3 (2%)	1 (5%)	2 (4%)	··	1 (2%)	1 (1%)
Eritrea	2 (1%)	··	··	2 (4%)	2 (4%)	1 (1%)
**Age range in the whole study**						
Infants only	18 (9%)	··	12 (22%)	4 (8%)	··	7 (6%)
Children 0−4	6 (3%)	··	1 (2%)	3 (6%)	3 (6%)	1 (1%)
Children 0−14	37 (19%)	3 (16%)	9 (17%)	11 (21%)	14 (30%)	17 (15%)
Children and adults ≥ 10	16 (8%)	3 (16%)	3 (6%)	2 (4%)	2 (4%)	13 (12%)
Adolescents 10−19	38 (20%)	1 (5%)	2 (4%)	11 (21%)	17 (36%)	24 (22%)
Children adolescents ≤ 19	24 (13%)	··	3 (6%)	4 (8%)	8 (17%)	21 (19%)
Adults ≥ 15	38 (20%)	12 (63%)	19 (35%)	13 (25%)	3 (6%)	19 (17%)
All ages	13 (7%)	··	5 (9%)	5 (9%)	··	8 (7%)
**Health facility setting**						
Health centre/clinic	121 (64%)	3 (16%)	28 (52%)	39 (74%)	30 (64%)	65 (59%)
Hospital	93 (49%)	2 (11%)	19 (35%)	36 (68%)	27 (57%)	50 (45%)
Home/community	29 (15%)	16 (84%)	20 (37%)	3 (6%)	2 (4%)	20 (18%)
National laboratory system	1 (1%)	··	··	··	··	1 (1%)
**Urban/rural**						
Urban only	11 (6%)	··	3 (6%)	4 (8%)	3 (6%)	7 (6%)
Rural only	21 (11%)	3 (16%)	5 (9%)	7 (13%)	5 (11%)	12 (11%)
Both urban and rural	158 (83%)	16 (84%)	46 (85%)	42 (79%)	39 (83%)	91 (83%)
**Study design**						
Cross‐sectional	66 (35%)	15 (79%)	17 (31%)	2 (4%)	20 (43%)	52 (47%)
Cohort	119 (63%)	4 (21%)	36 (67%)	50 (94%)	25 (53%)	56 (51%)
Quasi‐experimental/experimental	5 (3%)		1 (2%)	1 (2%)	2 (4%)	2 (2%)
**Study population**						
Living with HIV	83 (44%)	18 (95%)	51 (94%)	17 (32%)	8 (17%)	41 (37%)
On ART	107 (56%)	1 (5%)	2 (4%)	35 (66%)	39 (83%)	68 (62%)
**Study population size relevant for systematic review**						
Total *N*	647,904	40,367	215,307	184,410	23,683	363,589
Median (interquartile range)	377 (128, 965)	330 (60, 651)	225 (58, 1036)	542 (257, 2575)	399 (232, 674)	389 (164, 996)
**Key populations**						
Pregnant women	10 (5%)	2 (11%)	3 (6%)	3 (6%)	1 (2%)	6 (5%)
Experienced treatment failure	5 (3%)	··	··	1 (2%)	2 (4%)	4 (4%)
Orphans/vulnerable children	5 (3%)	··	2 (4%)	2 (4%)	1 (2%)	3 (3%)
Female sex workers	2 (1%)	2 (11%)	2 (4%)	··	··	2 (2%)
With non‐tuberculosis comorbidity	2 (1%)	··	····	··	1 (2%)	2 (2%)

The meta‐analysis pooled proportions across all HIV care continuum indicators were similar between children aged 0–14 and adolescents aged 15–19 with HIV (Table 3; Figures S1–S10 show the forest plots). Three‐fourths of children and adolescents with HIV were diagnosed and aware of their status (children: 72% [95% CI: 60%, 81%]; adolescents: 73% [95% CI: 66%, 79%]). More than 90% of children and adolescents with HIV aware of their status were on ART (children: 95% [95% CI: 89%, 97%]; adolescents: 93% [95% CI: 92%, 94%]). Five‐sixths of children and adolescents with HIV were retained in HIV care 12 months after ART initiation (children: 88% [95% CI: 76%, 95%], adolescents: 85% [95% CI: 74%, 92%]). Three‐fourths of children and adolescents with HIV on ART were adherent based on self‐ or caregiver reports (children: 77% [95% CI: 68%, 84%]; adolescents: 74% [95% CI: 63%, 83%]). Nine‐tenths of children and adolescents with HIV on ART had a viral load test (children: 90% [95% CI: 79%, 95%]; adolescents: 90% [95% CI: 78%, 95%]). Three‐fourths of children and adolescents with HIV on ART and had a viral load test achieved viral suppression using a standardized cutoff of 1000 copies/ml (children: 76% [95% CI: 72%, 79%]; adolescents: 78% [95% CI: 74%, 81%]).

**Table 3:** Pooled proportions of the HIV care continuum indicators among children and adolescents in eastern and southern Africa during the Universal Test and Treat (UTT) era, by age group

	Indicator	Children aged 0−14			Adolescents aged 15−19		
		Pooled proportion (95% CI)	Pooled *N*	Number of study countries	Pooled proportion (95% CI)	Pooled *N*	Number of study countries
Diagnosed and awareness of HIV status	(1) Proportion of children and adolescents with HIV who were diagnosed or aware of their HIV status (first 95‐95‐95 target)	72 (60, 81)	760	10	73 (66, 79)	1538	18
Being on antiretroviral therapy (ART) after diagnosis	(2a) Proportion of children and adolescents with HIV on ART sometime after diagnosis (second 95‐95‐95 target)	95 (89, 97)	34,112	40	93 (92, 94)	14,929	35
	(2b) Proportion of children and adolescents with HIV who rapidly initiated ART after diagnosis (<7 days)	81 (62, 91)	1240	8	80 (54, 93)	1196	6
Retention in HIV care	Proportion of children and adolescents having initiated ART who continuously attended clinic visits for:						
	(3a) 6 months	88 (53, 98)	7963	8	86 (57, 96)	5677	7
	(3b) 12 months	88 (76, 95)	4321	16	85 (74, 92)	31,116	13
	(3c) 24 months	84 (67, 92)	11,821	8	68 (34, 90)	6595	6
Adherence to ART	(4) Proportion of children and adolescents on ART who were adherent according to the following approaches:						
	(4a) self‐ or caregiver‐reported approach	77 (68, 84)	6688	19	74 (63, 83)	3579	16
	(4b) health facility‐reported approach	88 (84, 91)	4363	12	73 (58, 84)	3039	12
Viral suppression	(5a) Proportion of children and adolescents on ART with a viral load test	90 (79, 95)	16,581	13	90 (78, 95)	11,940	15
	(5b) Proportion of children and adolescents on ART and have a viral load test who were virally suppressed at a cutoff of: 1000 copies/ml	76 (72, 79)	168,943	101	78 (74, 81)	93,068	93

Abbreviation: CI, confidence interval.

Sex differences in the HIV care continuum indicators differed across countries. For example, in South Africa, a higher proportion of male adolescents (58%) had initiated ART compared to female adolescents (49%) [38], while in Lesotho, a higher proportion of female adolescents (93%) had initiated ART compared to males (84%) [42]. Retention in HIV care was generally higher among females than males. Adherence was similar between males and females [145, 151, 157]. There were no clear patterns of sex differences in viral suppression proportions.

There was some variation in the definitions of retention among the 53 included studies: 22 defined loss to follow‐up as non‐attendance to clinic for ≥3 months, 10 defined it as ≥6 months, while the rest did not provide a clear definition of loss to follow‐up or simply reported continuous clinic attendance during the study's observation period. Despite the variation in definition, there were no clear study‐level patterns in the retention proportions according to the different definitions of loss to follow‐up.

Of the 47 studies reporting on adherence to ART, 21 studies measured adherence from self‐report or caregiver reports, while the rest measured adherence from health facility reports. Forty‐two studies included some definitions of missed doses prior to the measurement of adherence, with the time of recall or review ranging from 7 days to 1 year. Of these, 29 studies used a cutoff of 95% for good adherence. While the proportions on ART adherence were similar between children and adolescents based on self‐ and caregiver reports, children aged 0–14 years living with HIV on ART had higher proportions of ART adherence based on health facility reports than adolescents aged 15–19 (children: 88% [95% CI: 84%, 91%]; adolescents: 73% [95% CI: 58%, 84%]).

## 4 DISCUSSION

This study comprehensively described the HIV care continuum for children and adolescents with HIV in eastern and southern Africa during the UTT era. Consistent with UTT expectations [80], the proportion of children diagnosed with HIV who initiated ART may have reached the 95% UNAIDS target, and came close for adolescents, reaching 93%. However, gaps continue to exist on other HIV care continuum indicators during the first few years of UTT implementation. Among all children and adolescents with HIV, one in four were still undiagnosed. Among children and adolescents with HIV on ART, one in six were not retained in HIV care and one in four were not adherent. Among children and adolescents with HIV on ART with a viral load test at least 6 months after initiation, one in four had not achieved viral suppression. Altogether, these results mean that using a common denominator of all children and adolescents with HIV, three in five were retained in HIV care 12 months after ART initiation, half were adherent to ART and less than half achieved viral suppression. Children aged 0–14 had similar experiences on the HIV care continuum as adolescents aged 15–19, except for retention, where adolescents experienced a lower retention proportion by 24 months than children. Finally, sex differences in the HIV care continuum indicator differed by country.

Pooled meta‐analysis estimates of the HIV care continuum seem similar to official UNAIDS estimates, which uses a model‐based approach using parameters from country surveillance data, household survey data, existing HIV cohort and trial data, and expert opinion [215]. Between 2016 and 2022, the proportion of children aged 0–14 with HIV who were aware of their status ranged from 55% to 71%, the proportion with HIV aware of their status on ART was at 90% and the proportion on ART who were virally suppressed ranged from 67% to 80% [17]. The pooled meta‐analysis results in this study were 72%, 95% and 76%, respectively. This consistency provides strength to our study's results complementing UNAIDS estimates, specifically on retention in HIV care and adherence to ART [215]. Future changes to HIV models that underlie the official UNAIDS estimates may also use the pooled estimates from this study as model parameters.

Except for ART initiation, meta‐analysis results from this study were also consistent with previously published meta‐analysis using data from the pre‐UTT era. One review found that the pooled proportions among children aged 0–14 with HIV in low‐ and middle‐income countries from 2010 to 2016 who were retained in care after ART initiation were 81% by 6 months, 81% by 12 months and 80% by 24 months [6]; another study found that retention among adolescents aged 10−19 in sub‐Saharan Africa was 85% during the period of 2010−2016 [11]. The same trend in retention in HIV care before and after UTT was also observed in a systematic review among adults with HIV in sub‐Saharan Africa [216]. Pooled estimates on ART adherence were similar to two systematic reviews of adolescents aged 10−19 in sub‐Saharan Africa, where the adherence proportions ranged from 64% to 90% among studies published before 2016 [8, 12]. Pooled estimates on viral suppression were similar to another meta‐analysis of children and adolescents aged 18 and younger from 2010 to 2015, with the pooled proportion at 73% [7].

An increase in the proportion of children and adolescents with HIV initiating ART with the introduction of UTT, and the similarity of the proportions of children and adolescents on ART who were retained in HIV care, were adherent to ART, and achieved viral suppression before and after UTT means that there was still an increase in the proportion of children and adolescents with HIV who were retained in HIV care, were adherent to ART and achieved viral suppression. However, there are several barriers to improving HIV outcomes among children and adolescents with HIV: supply‐side barriers include inconsistent availability of paediatric ARTs and accessibility to the health facility, while demand‐side barriers include stigma and discrimination, serious family life events and self‐efficacy to treatment regimen [13, 217]. Additionally, children require the support of an adult caregiver to administer treatment, and some ART may not be palatable to younger children [218]. Therefore, to continue engaging children and adolescents to stay along the continuum, interventions must leverage community support structures to empower children and adolescents to maintain HIV care and must be tailored to age‐specific needs [219, 220, 221]. Adolescents unlike children are generally expected to take responsibility for their care and this may impact on adherence and viral suppression.

This study has some limitations. First, the meta‐analysis was limited to peer‐reviewed articles that were indexable in English language databases and most studies did not have a representative sample of their country. Most estimates came from an urban, health facility setting, which may overestimate the true proportions. Nonetheless, the total pooled sample size represents about one‐third of all children and adolescents with HIV in the eastern and southern African region. Second, we made assumptions in the meta‐analysis regarding some indicators of the care continuum definitions, that may also overestimate the true proportion as they fail to account for disengagement and reengagement in the care continuum [222]. For example, when pooling estimates of viral suppression, we did not anchor them on a fixed time point from ART initiation since the majority of the included studies assessed viral suppression only cross‐sectionally without a specific time frame from ART initiation. Third, the meta‐analysis indicators were conditional proportions, with the denominator an earlier indicator in the HIV care continuum. We decided on conditional proportions to better represent the data extracted from the included studies, although future studies may consider reporting unconditional proportions as well. We only estimated the pooled proportions at the regional level and across a wide age band because of the heterogeneity in health system contexts and study population demographics across studies. The width of the CIs reflects this heterogeneity, as well as the sparsity of available pooled studies. Any further disaggregation would have led to incoherent estimates relative to the overall pooled proportions. The pooled proportions are also the median estimate, which is robust to outliers and studies with small sample sizes [23], so we did not apply a minimum sample size criterion to the studies. Finally, this review does not encompass most post‐COVID era changes in HIV service delivery. Future reviews may explore these developments as more studies become available.

## 5 CONCLUSIONS

The pooled results of the HIV care continuum among children and adolescents in eastern and southern Africa in the UTT era reveal high levels of ART initiation but suboptimal levels of awareness of HIV diagnosis, retention in HIV care, adherence and viral suppression. Future strategic directions for HIV programmes should consider locally informed, systemic approaches to improve coverage of diagnosis and continued engagement in HIV care beyond ART initiation.

## COMPETING INTERESTS

The authors declare no competing interests.

## AUTHORS’ CONTRIBUTIONS

NJLH, DKN and CRL jointly conceptualized the research question and the search strategy. NJLH, SL and MB conducted the systematic review, with DKN and CRL serving to resolve any conflicts between them. NJLH, DKN, CRL, SL and MB jointly developed and piloted the data extraction table. NJLH and MB filled out the data extraction table and conducted the quality assessment. NJLH analysed the data for the meta‐analysis and generated all figures and tables, with some assistance from MB. NJLH also drafted the primary draft of this manuscript, with DKN, CRL, SL, AA, MB, LF and MB‐D providing substantial edits in later versions of the draft.

## FUNDING

This project was funded by the Johns Hopkins Bloomberg School of Public Health Department of Epidemiology.

## Supporting information




**File S1**. Additional tables and figures. Search criteria, summary tables of the systematic review and forest plots.

## Data Availability

The table of articles is available in the Supplement. The table of extracted summary statistics from all included articles is available for download at the following URL: https://bit.ly/calhiv_stats

## References

[1] Joint United Nations Programme on HIV/AIDS . The urgency of now: AIDS at a crossroads. Geneva: UNAIDS; 2024.

[2] Hayes RJ , Donnell D , Floyd S , Mandla N , Bwalya J , Sabapathy K , et al. Effect of Universal Testing and Treatment on HIV Incidence — HPTN 071 (PopART). N Engl J Med. 2019;381(3):207–218. 31314965 10.1056/NEJMoa1814556 PMC6587177

[3] Teeraananchai S , Boettiger DC , Lertpiriyasuwat C , Triamwichanon R , Benjarattanaporn P , Phanuphak N . The impact of same‐day and rapid ART initiation under the Universal Health Coverage programme on HIV outcomes in Thailand: a retrospective real‐life cohort study. J Int AIDS Soc. 2025;28(1):e26406. 39748224 10.1002/jia2.26406 PMC11695198

[4] World Health Organization . Consolidated guidelines on the use of antiretroviral drugs for treating and preventing HIV infection: recommendations for a public health approach. 2nd ed. Geneva: World Health Organization; 2016. 27466667

[5] Gardner EM , McLees MP , Steiner JF , del Rio C , Burman WJ . The spectrum of engagement in HIV care and its relevance to test‐and‐treat strategies for prevention of HIV infection. Clin Infect Dis. 2011;52(6):793–800. 21367734 10.1093/cid/ciq243 PMC3106261

[6] Carlucci JG , Liu Y , Clouse K , Vermund SH . Attrition of HIV‐positive children from HIV services in low and middle‐income countries. AIDS. 2019;33(15):2375–2386. 31764102 10.1097/QAD.0000000000002366 PMC6905128

[7] Boerma RS , Boender TS , Bussink AP , Calis JCJ , Bertagnolio S , Rinke de Wit TF , et al. Suboptimal viral suppression rates among HIV‐infected children in low‐ and middle‐income countries: a meta‐analysis. Clin Infect Dis. 2016;63(12):1645–1654. 27660236 10.1093/cid/ciw645

[8] Ammon N , Mason S , Corkery JM . Factors impacting antiretroviral therapy adherence among human immunodeficiency virus–positive adolescents in sub‐Saharan Africa: a systematic review. Public Health. 2018;157:20–31. 29501984 10.1016/j.puhe.2017.12.010

[9] Green D , Tordoff DM , Kharono B , Akullian A , Bershteyn A , Morrison M , et al. Evidence of sociodemographic heterogeneity across the HIV treatment cascade and progress towards 90‐90‐90 in sub‐Saharan Africa—a systematic review and meta‐analysis. J Int AIDS Soc. 2020;23(3):e25470. 32153117 10.1002/jia2.25470 PMC7062634

[10] Gelaw B , Mulatu G , Tesfa G , Marew C , Chekole B , Alebel A . Magnitude and associated factors of virological failure among children on ART in Bahir Dar Town public health facilities, Northwest Ethiopia: a facility based cross‐sectional study. Ital J Pediatr. 2021;47(1):1–9. 33823890 10.1186/s13052-021-01030-7 PMC8025328

[11] Leshargie CT , Demant D , Burrowes S , Frawley J . The proportion of loss to follow‐up from antiretroviral therapy (ART) and its association with age among adolescents living with HIV in sub‐Saharan Africa: a systematic review and meta‐analysis. PLoS One. 2022;17(8):e0272906. 35951621 10.1371/journal.pone.0272906 PMC9371308

[12] Mengesha MM , Teshome A , Ajema D , Tura AK , Hallström IK , Jerene D . The association between HIV diagnosis disclosure and adherence to anti‐retroviral therapy among adolescents living with HIV in sub‐Saharan Africa: a systematic review and meta‐analysis. PLoS One. 2023;18(5):e0285571. 37167342 10.1371/journal.pone.0285571 PMC10174542

[13] Enane LA , Davies MA , Leroy V , Edmonds A , Apondi E , Adedimeji A , et al. Traversing the cascade: urgent research priorities for implementing the ‘treat all’ strategy for children and adolescents living with HIV in sub‐Saharan Africa. J Virus Erad. 2018;4:40–46. 30515313 10.1016/S2055-6640(20)30344-7 PMC6248846

[14] Veritas Health Innovation . Covidence systematic review software. Melbourne. Accessed June 10, 2024. www.covidence.org

[15] World Health Organization . HIV strategic information for impact: cascade data use manual: to identify gaps in HIV and health services for programme improvement: user manual. Geneva: World Health Organization; 2018.

[16] Munn Z , Moola S , Lisy K , Riitano D , Tufanaru C . Methodological guidance for systematic reviews of observational epidemiological studies reporting prevalence and cumulative incidence data. Int J Evid Based Healthc. 2015;13(3):147–153. 26317388 10.1097/XEB.0000000000000054

[17] Joint United Nations Program on HIV/AIDS (UNAIDS) . The Path that Ends AIDS: UNAIDS Global AIDS Update 2023. Geneva: UNAIDS; 2023.

[18] Stijnen T , Hamza TH , Özdemir P . Random effects meta‐analysis of event outcome in the framework of the generalized linear mixed model with applications in sparse data. Stat Med. 2010;29(29):3046–3067. 20827667 10.1002/sim.4040

[19] Stannah J , Soni N , Lam JKS , Giguère K , Mitchell KM , Kronfli N , et al. Trends in HIV testing, the treatment cascade, and HIV incidence among men who have sex with men in Africa: a systematic review and meta‐analysis. Lancet HIV. 2023;10(8):e528–e542. 37453439 10.1016/S2352-3018(23)00111-X PMC11403132

[20] Johnson LF , Kariminia A , Trickey A , Yiannoutsos CT , Ekouevi DK , Minga AK , et al. Achieving consistency in measures of HIV‐1 viral suppression across countries: derivation of an adjustment based on international antiretroviral treatment cohort data. J Int AIDS Soc. 2021;24(Suppl 5):e25776. 34546623 10.1002/jia2.25776 PMC8454679

[21] Clopper CJ , Pearson ES . The use of confidence or fiducial limits illustrated in the case of the binomial. Biometrika. 1934;26(4):404–413.

[22] Birdthistle I , Tanton C , Tomita A , de Graaf K , Schaffnit SB , Tanser F , et al. Recent levels and trends in HIV incidence rates among adolescent girls and young women in ten high‐prevalence African countries: a systematic review and meta‐analysis. Lancet Glob Health. 2019;7(11):e1521–e1540. 31607465 10.1016/S2214-109X(19)30410-3 PMC7025003

[23] Lin L , Chu H . Meta‐analysis of proportions using generalized linear mixed models. Epidemiology. 2020;31(5):713–717. 32657954 10.1097/EDE.0000000000001232 PMC7398826

[24] Hartung J , Knapp G . A refined method for the meta‐analysis of controlled clinical trials with binary outcome. Stat Med. 2001;20(24):3875–3889. 11782040 10.1002/sim.1009

[25] Anzures‐Cabrera J , Higgins JPT . Graphical displays for meta‐analysis: an overview with suggestions for practice. Res Synth Methods. 2010;1(1):66–80. 26056093 10.1002/jrsm.6

[26] Bossard C , Chihana M , Nicholas S , Mauambeta D , Weinstein D , Conan N , et al. HIV, sexual violence, and termination of pregnancy among adolescent and adult female sex workers in Malawi: a respondent‐driven sampling study. PLoS One. 2022;17(12): e0279692. 36584132 10.1371/journal.pone.0279692 PMC9803093

[27] Brown K , Williams DB , Kinchen S , Saito S , Radin E , Patel H , et al. Status of HIV epidemic control among adolescent girls and young women aged 15–24 years—seven African countries, 2015–2017. MMWR Morb Mortal Wkly Rep. 2018;67(1):29–32. 29329280 10.15585/mmwr.mm6701a6 PMC5769792

[28] Burgos‐Soto J , Ben Farhat J , Alley I , Ojuka P , Mulogo E , Kise‐Sete T , et al. HIV epidemic and cascade of care in 12 east African rural fishing communities: results from a population‐based survey in Uganda. BMC Public Health. 2020;20(1):970. 32560717 10.1186/s12889-020-09121-6 PMC7305611

[29] Conan N , Badawi M , Chihana ML , Wanjala S , Kingwara L , Mambula C , et al. Two‐fold increase in the HIV viral load suppression rate along with decreased incidence over six years in Ndhiwa sub‐county, Kenya. Trop Med Int Health. 2021;26(12):1609–1615. 34637172 10.1111/tmi.13688 PMC9298256

[30] Conan N , Simons E , Chihana ML , Ohler L , FordKamara E , Mbatha M , et al. Increase in HIV viral suppression in KwaZulu‐Natal, South Africa: community‐based cross sectional surveys 2018 and 2013. What remains to be done? PLoS One. 2022;17(3):e0265488. 35324923 10.1371/journal.pone.0265488 PMC8946728

[31] Conan N , Paye CP , Ortuno R , Chijuwa A , Chiwandira B , Goemaere E , et al. What gaps remain in the HIV cascade of care? Results of a population‐based survey in Nsanje District, Malawi. PLoS One. 2021;16(4):e0248410. 33886575 10.1371/journal.pone.0248410 PMC8061928

[32] Floyd S , Shanaube K , Yang B , Schaap A , Griffith S , Phiri M , et al. HIV testing and treatment coverage achieved after 4 years across 14 urban and peri‐urban communities in Zambia and South Africa: an analysis of findings from the HPTN 071 (PopART) trial. PLoS Med. 2020;17(4):e1003067. 32240156 10.1371/journal.pmed.1003067 PMC7117659

[33] Gibbs A , Reddy T , Closson K , Cawood C , Khanyile D , Hatcher A . Intimate partner violence and the HIV care and treatment cascade among adolescent girls and young women in DREAMS, South Africa. J Acquir Immune Defic Syndr. 2022;89(2):136–142. 34723930 10.1097/QAI.0000000000002843 PMC8740602

[34] Hakim AJ , Bolo A , Coy KC , Achut V , Katoro J , Caesar G , et al. Progress toward the UNAIDS 90‐90‐90 targets among female sex workers and sexually exploited female adolescents in Juba and Nimule, South Sudan. BMC Public Health. 2022;22(1):132. 35045854 10.1186/s12889-022-12533-1 PMC8767749

[35] Jonnalagadda S , Auld A , Jahn A , Saito S , Bello G , Sleeman K , et al. Opportunities for closing the gap in HIV diagnosis, treatment, and viral load suppression in children in Malawi: results from a 2015–2016 Population‐based HIV Impact Assessment Survey. Pediatr Infect Dis J. 2021;40(11):1011–1018. 34382613 10.1097/INF.0000000000003288

[36] Low A , Teasdale C , Brown K , Barradas DT , Mugurungi O , Sachathep K , et al. Human immunodeficiency virus infection in adolescents and mode of transmission in southern Africa: a multinational analysis of population‐based survey data. Clin Infect Dis. 2021;73(4):594–604. 33912973 10.1093/cid/ciab031 PMC8366830

[37] Lulseged S , Belete W , Ahmed J , Gelibo T , Teklie H , West CW , et al. Factors associated with unawareness of HIV‐positive status in urban Ethiopia: evidence from the Ethiopia population‐based HIV impact assessment 2017–2018. PLoS One. 2021;16(8):e0255163. 34380145 10.1371/journal.pone.0255163 PMC8357455

[38] Marinda E , Simbayi L , Zuma K , Zungu N , Moyo S , Kondlo L , et al. Towards achieving the 90‐90‐90 HIV targets: results from the South African 2017 national HIV survey. BMC Public Health. 2020;20(1):1375. 32907565 10.1186/s12889-020-09457-z PMC7487872

[39] Mutisya I , Muthoni E , Ondondo RO , Muthusi J , Omoto L , Pahe C , et al. A national household survey on HIV prevalence and clinical cascade among children aged ≤15 years in Kenya (2018). PLoS One. 2022;17(11):e0277613. 36417391 10.1371/journal.pone.0277613 PMC9683548

[40] Ntombela NP , Kharsany ABM , Soogun A , Yende‐Zuma N , Baxter C , Kohler HP , et al. Viral suppression among pregnant adolescents and women living with HIV in rural KwaZulu‐Natal, South Africa: a cross sectional study to assess progress towards UNAIDS indicators and implications for HIV epidemic control. Reprod Health. 2022;19(1):1–13. 35550580 10.1186/s12978-022-01419-5 PMC9097174

[41] Teasdale CA , Zimba R , Abrams EJ , Sachathep K , Ndagije F , Nuwagaba‐Biribonwoha H , et al. Estimates of the prevalence of undiagnosed HIV among children living with HIV in Eswatini, Lesotho, Malawi, Namibia, Tanzania, Zambia, and Zimbabwe from 2015 to 2017: an analysis of data from the cross‐sectional Population‐based HIV Impact Assessment Survey. Lancet HIV. 2022;9(2):e91–e101. 35120641 10.1016/S2352-3018(21)00291-5 PMC10350876

[42] Thin K , Frederix K , McCracken S , Letsie M , Low A , Patel H , et al. Progress toward HIV epidemic control in Lesotho. AIDS. 2019;33(15):2393–2401. 31764104 10.1097/QAD.0000000000002351 PMC8064033

[43] Woldesenbet S , Kufa T , Cheyip M , Ayalew K , Lombard C , Manda S , et al. Awareness of HIV‐positive status and linkage to treatment prior to pregnancy in the ‘test and treat’ era: a national antenatal sentinel survey, 2017, South Africa. PLoS One. 2020;15(3):e0229874. 32168356 10.1371/journal.pone.0229874 PMC7069609

[44] Woldesenbet S , Cheyip M , Lombard C , Manda S , Ayalew K , Kufa T , et al. Progress towards the UNAIDS 95‐95‐95 targets among pregnant women in South Africa: results from the 2017 and 2019 national antenatal HIV sentinel surveys. PLoS One. 2022;17(7):e0271564. 35862306 10.1371/journal.pone.0271564 PMC9302844

[45] Adedimeji A , Edmonds A , Hoover D , Shi Q , Sinayobye JD , Nduwimana M , et al. Characteristics of HIV‐infected children at enrollment into care and at antiretroviral therapy initiation in Central Africa. PLoS One. 2017;12(1):e0169871. 28081230 10.1371/journal.pone.0169871 PMC5230784

[46] Arpadi S , Lamb M , Nzeyimana IN , Vandebriel G , Anyalechi G , Wong M , et al. Better outcomes among HIV‐infected Rwandan children 18–60 months of age after the implementation of ‘Treat All’. J Acquir Immune Defic Syndr. 2019;80(3):e74–e83. 30422899 10.1097/QAI.0000000000001907 PMC6392203

[47] Augustine N , Philip O , Kumar A , Simukai Z , Owen M , Dumisani M , et al. Gaps in the care cascade among human immunodeficiency virus‐exposed infants born in 2017 in Mashonaland East Province of Zimbabwe. J Glob Infect Dis. 2021;13(2):72–79. 34194173 10.4103/jgid.jgid_171_19 PMC8213089

[48] Ayieko J , Petersen ML , Charlebois ED , Brown LB , Clark TD , Kwarisiima D , et al. A patient‐centered multicomponent strategy for accelerated linkage to care following community‐wide HIV testing in rural Uganda and Kenya. J Acquir Immune Defic Syndr. 2019;80(4):414–422. 30807481 10.1097/QAI.0000000000001939 PMC6410970

[49] Bacha JM , Dlamini S , Anabwani F , Gwimile J , Kanywa JB , Farirai J , et al. Achieving antiretroviral therapy uptake and viral suppression among children and adolescents living with HIV in the UNAIDS 90‐90‐90 era across six countries in eastern and southern Africa‐lessons from the BIPAI network. J Acquir Immune Defic Syndr. 2022;90(3):300–308. 35364599 10.1097/QAI.0000000000002957

[50] Bachanas P , Alwano MG , Lebelonyane R , Block L , Behel S , Raizes E , et al. Finding, treating and retaining persons with HIV in a high HIV prevalence and high treatment coverage country: results from the Botswana Combination Prevention Project. PLoS One. 2021;16(4):e0250211. 33882092 10.1371/journal.pone.0250211 PMC8059857

[51] Baisley KJ , Seeley J , Siedner MJ , Koole K , Matthews P , Tanser F , et al. Findings from home‐based HIV testing and facilitated linkage after scale‐up of test and treat in rural South Africa: young people still missing. HIV Med. 2019;20(10):704–708. 31454139 10.1111/hiv.12787 PMC6788950

[52] Bajaria S , Exavery A , Toroka N , Abdul R . Poor linkage to care for HIV‐positive OVC with disabled caregivers: a longitudinal study in Tanzania. BMC Public Health. 2021;21(1):365. 33593313 10.1186/s12889-021-10415-6 PMC7887816

[53] Bekele A , Hrapcak S , Mohammed JA , Yimam JA , Tilahun T , Antefe T , et al. Rates of confirmatory HIV testing, linkage to HIV services, and rapid initiation of antiretroviral treatment among newly diagnosed children living with HIV in Ethiopia: perspectives from caregivers and healthcare workers. BMC Pediatr. 2022;22(1):1–10. 36572846 10.1186/s12887-022-03784-3 PMC9791729

[54] Bianchi F , Cohn J , Sacks E , Bailey R , Lemaire JF , Machekano R , et al. Evaluation of a routine point‐of‐care intervention for early infant diagnosis of HIV: an observational study in eight African countries. Lancet HIV. 2019;6(6):e373–e381. 30987937 10.1016/S2352-3018(19)30033-5

[55] Boeke CE , Joseph J , Wang M , Abate ZM , Atem C , Coulibaly KD , et al. Point‐of‐care testing can achieve same‐day diagnosis for infants and rapid ART initiation: results from government programmes across six African countries. J Int AIDS Soc. 2021;24(3):1–9. 10.1002/jia2.25677 PMC7981587 33745234

[56] Bolton‐Moore C , Sikazwe I , Mubiana‐Mbewe M , Munthali G , Wa Mwanza M , Savory T , et al. Temporal changes in paediatric and adolescent HIV outcomes across the care continuum in Zambia: an interrupted time‐series analysis. Lancet HIV. 2022;9(8):e563–e573. 35905754 10.1016/S2352-3018(22)00127-8 PMC9394542

[57] Denoeud‐Ndam L , Stecker C , Andifasi P , Mushavi A , Maphosa T , Zondo M , et al. Implementation and uptake of raltegravir granules in newborns diagnosed with HIV through birth testing in maternity settings in Zimbabwe during the COVID‐19 pandemic. Pediatr Infect Dis J. 2023;42:573–575. 37000925 10.1097/INF.0000000000003906 PMC10289070

[58] Dougherty G , Abena T , Abesselo JP , Banda JN , Biyaga TP , Boccanera R , et al. Improving services for HIV‐exposed infants in Zambia and Cameroon using a quality improvement collaborative approach. Glob Health Sci Pract. 2021;9(2):399–411. 34234027 10.9745/GHSP-D-20-00550 PMC8324201

[59] Dougherty G , Panya M , Madevu‐Matson C , Anyalechi GE , Clarke K , Fayorsey R , et al. Reaching the first 90: improving inpatient pediatric provider‐initiated HIV testing and counseling using a quality improvement collaborative strategy in Tanzania. J Assoc Nurses AIDS Care. 2019;30(6):682–690. 30817370 10.1097/JNC.0000000000000066 PMC6698429

[60] Etoori D , Kerschberger B , Staderini N , Ndlangamandla M , Nhlabatsi B , Jobanputra K , et al. Challenges and successes in the implementation of option B+ to prevent mother‐to‐child transmission of HIV in southern Swaziland. BMC Public Health. 2018;18(1):374. 29558896 10.1186/s12889-018-5258-3 PMC5859825

[61] Finocchario‐Kessler S , Wexler C , Brown M , Goggin K , Lwembe R , Nazir N , et al. Piloting the feasibility and preliminary impact of adding birth HIV polymerase chain reaction testing to the early infant diagnosis guidelines in Kenya. Pediatr Infect Dis J. 2021;40:741–745. 33990521 10.1097/INF.0000000000003172 PMC8274583

[62] Graça D , Elliott RJ , Magalo M , Muianga M , Mussagi AC , Chongo M , et al. Monitoring and evaluation of HIV screening and testing of hospitalized infants and their mothers. Public Health Action. 2022;12(2):68–73. 35734006 10.5588/pha.21.0074 PMC9176192

[63] Grasso MA , Hamunime N , Maher AD , Cockburn D , Williams DB , Taffa N , et al. Improving the benefits of HIV testing and referrals in large household surveys through active linkages to care: lessons and recommendations from the Namibia population‐based HIV impact assessment (NAMPHIA), 2017. AIDS Care. 2021;33(10):1308–1311. 33486974 10.1080/09540121.2021.1874266 PMC10988420

[64] Gross J , Medley A , Rivadeneira E , Battey K , Srivastava M , Grillo M , et al. Considerations to improve pediatric HIV testing and close the treatment gap in 16 African countries. Pediatr Infect Dis J. 2023;42(2):110–118. 36638395 10.1097/INF.0000000000003778 PMC10935587

[65] Jubilee M , Park FJ , Chipango K , Pule K , Machinda A , Taruberekera N . HIV index testing to improve HIV positivity rate and linkage to care and treatment of sexual partners, adolescents and children of PLHIV in Lesotho. PLoS One. 2019;14(3):e0212762. 30917167 10.1371/journal.pone.0212762 PMC6436679

[66] Kalawan V , Naidoo K , Archary M . Impact of routine birth early infant diagnosis on neonatal HIV treatment cascade in eThekwini district, South Africa. South Afr J HIV Med. 2020;21(1):1–5. 10.4102/sajhivmed.v21i1.1084 PMC7276481 32537251

[67] Kim MH , Ahmed S , Hosseinipour MC , Yu X , Nguyen C , Chimbwandira F , et al. Brief report: impact of option B+ on the infant PMTCT cascade in Lilongwe, Malawi. J Acquir Immune Defic Syndr. 2015;70(1):99–103. 26322670 10.1097/QAI.0000000000000692 PMC4537054

[68] MacKellar D , Williams D , Bhembe B , Dlamini M , Byrd J , Dube L , et al. Peer‐delivered linkage case management and same‐day ART initiation for men and young persons with HIV infection—Eswatini, 2015–2017. MMWR Morb Mortal Wkly Rep. 2018;67(23):663–667. 29902168 10.15585/mmwr.mm6723a3 PMC6002033

[69] Masoza TS , Rwezaula R , Msanga DR , Chami N , Kabirigi J , Ambrose E , et al. Prevalence and outcome of HIV infected children admitted in a tertiary hospital in northern Tanzania. BMC Pediatr. 2022;22(1):1–9. 35189841 10.1186/s12887-022-03105-8 PMC8860281

[70] Matsinhe M , Bollinger T , Lee N , Loquiha O , Meggi B , Mabunda N , et al. Inpatient point‐of‐care HIV early infant diagnosis in Mozambique to improve case identification and linkage to antiretroviral therapy. Glob Health Sci Pract. 2021;9(1):31–39. 33684058 10.9745/GHSP-D-20-00611 PMC8087433

[71] Munthali T , Michelo C , Mee P , Moyo C , Kashoka A , Liswaniso L , et al. Impact of WHO guidelines on trends in HIV testing and ART initiation among children living with HIV in Zambia. AIDS Res Ther. 2020;17(1):1–10. 32408890 10.1186/s12981-020-00277-0 PMC7226945

[72] Mwango LK , Stafford KA , Blanco NC , Lavoie MC , Mujansi M , Nyirongo N , et al. Index and targeted community‐based testing to optimize HIV case finding and ART linkage among men in Zambia. J Int AIDS Soc. 2020;23(Suppl 2):e25520. 32589360 10.1002/jia2.25520 PMC7319128

[73] Pathmanathan I , Nelson R , de Louvado A , Thompson R , Pals S , Casavant I , et al. High coverage of antiretroviral treatment with annual home‐based HIV testing, follow‐up linkage services, and implementation of test and start: findings from the Chókwè Health Demographic Surveillance System, Mozambique, 2014–2019. J Acquir Immune Defic Syndr. 2021;86(4):e97–e105. 33252546 10.1097/QAI.0000000000002583 PMC7970427

[74] Ross J , Ribakare M , Remera E , Murenzi G , Munyaneza A , Hoover DR , et al. High levels of viral load monitoring and viral suppression under Treat All in Rwanda—a cross‐sectional study. J Int AIDS Soc. 2020;23(6):1–6. 10.1002/jia2.25543 PMC7293767 32536033

[75] Rufu A , Chitimbire VTS , Nzou C , Timire C , Owiti P , Harries AD , et al. Implementation of the ‘test and treat’ policy for newly diagnosed people living with HIV in Zimbabwe in 2017. Public Health Action. 2018;8(3):145–150. 30271732 10.5588/pha.18.0030 PMC6147065

[76] Shah P , Kibel M , Ayuku D , Lobun R , Ayieko J , Keter A , et al. A pilot study of ‘peer navigators’ to promote uptake of HIV testing, care and treatment among street‐connected children and youth in Eldoret, Kenya. AIDS Behav. 2019;23(4):908–919. 30269232 10.1007/s10461-018-2276-1 PMC6458975

[77] Sutcliffe CG , Mutanga JN , Moyo N , Schue JL , Hamahuwa M , Thuma PE , et al. Acceptability and feasibility of testing for HIV infection at birth and linkage to care in rural and urban Zambia: a cross‐sectional study. BMC Infect Dis. 2020;20(1):1–10. 10.1186/s12879-020-4947-6 PMC7079396 32183751

[78] Tapera T , Willis N , Madzeke K , Napei T , Mawodzeke M , Chamoko S , et al. Effects of a peer‐led intervention on HIV care continuum outcomes among contacts of children, adolescents, and young adults living with HIV in Zimbabwe. Glob Health Sci Pract. 2019;7(4):575–584. 31852741 10.9745/GHSP-D-19-00210 PMC6927836

[79] Technau KG , Strehlau R , Patel F , Shiau S , Burke M , Conradie M , et al. 12‐month outcomes of HIV‐infected infants identified at birth at one maternity site in Johannesburg, South Africa: an observational cohort study. Lancet HIV. 2018;5(12):e706–e714. 30416043 10.1016/S2352-3018(18)30251-0 PMC6336389

[80] Tymejczyk O , Brazier E , Wools‐Kaloustian K , Davies MA , DiLorenzo M , Edmonds A , et al. Impact of universal antiretroviral treatment eligibility on rapid treatment initiation among young adolescents with human immunodeficiency virus in sub‐Saharan Africa. J Infect Dis. 2020;222(5):755–764. 31682261 10.1093/infdis/jiz547 PMC7530553

[81] Gill MM , Natumanya EK , Hoffman HJ , Okomo G , Taasi G , Guay L , et al. Active pediatric HIV case finding in Kenya and Uganda: a look at missed opportunities along the prevention of mother‐to‐child transmission of HIV (PMTCT) cascade. PLoS One. 2020;15(6):e0233590. 32484815 10.1371/journal.pone.0233590 PMC7266341

[82] Ahonkhai AA , Aliyu MH , Audet CM , Bravo M , Simmons M , Claquin G , et al. Poor retention and care‐related sex disparities among youth living with HIV in rural Mozambique. PLoS One. 2021;16(5):e0250921. 34019582 10.1371/journal.pone.0250921 PMC8139489

[83] Alemayehu T , Abebe W . Second line anti‐retroviral therapy failure in a pediatric cohort of an Ethiopian tertiary hospital: a retrospective observational study. Sci Rep. 2020;10(1):8699. 32457309 10.1038/s41598-020-65714-6 PMC7250842

[84] Alhaj M , Amberbir A , Singogo E , Banda V , van Lettow M , Matengeni A , et al. Retention on antiretroviral therapy during Universal Test and Treat implementation in Zomba district, Malawi: a retrospective cohort study. J Int AIDS Soc. 2019;22(2):1–7. 10.1002/jia2.25239 PMC6367572 30734510

[85] Amzel A , Srivastava M , Isavwa A , Sanders J , Tumbare E , Membe I , et al. Community‐based interventions to reach 95‐95‐95 for children and adolescents: an exploratory programmatic review from Lesotho. J Acquir Immune Defic Syndr. 2018;78:S81–S87. 29994829 10.1097/QAI.0000000000001735

[86] Antelman G , Jahanpour O , Machalo T , Audi C , van de Ven R , Rutaihwa M , et al. Adolescent support club attendance and self‐efficacy associated with HIV treatment outcomes in Tanzania. PLOS Glob Public Health. 2022;2(10):e0000065. 36962483 10.1371/journal.pgph.0000065 PMC10021176

[87] Barnhart DA , Uwamariya J , Nshimyumuremyi JN , Mukesharurema G , Anderson T , Ndahimana JD , et al. Receipt of a combined economic and peer support intervention and clinical outcomes among HIV‐positive youth in rural Rwanda: a retrospective cohort. PLOS Glob Public Health. 2022;2(6):e0000492. 36962346 10.1371/journal.pgph.0000492 PMC10021781

[88] Bimer KB , Sebsibe GT , Desta KW , Zewde A , Sibhat MM . Incidence and predictors of attrition among children attending antiretroviral follow‐up in public hospitals, Southern Ethiopia, 2020: a retrospective study. BMJ Paediatr Open. 2021;5(1):e001135. 10.1136/bmjpo-2021-001135 PMC8386224 34514177

[89] Biru M , Hallström I , Lundqvist P , Jerene D . Rates and predictors of attrition among children on antiretroviral therapy in Ethiopia: a prospective cohort study. PLoS One. 2018;13(2):e0189777. 29408897 10.1371/journal.pone.0189777 PMC5800538

[90] Biyazin Y , Wondwossen K , Wubie AB , Getachew M , Gebremichael B . Survival and predictors of mortality among HIV‐positive children on antiretroviral therapy in public hospitals. J Pharmaceut Policy Pract. 2022;15(1):1–13. 10.1186/s40545-022-00448-6 PMC9382771 35978382

[91] Brown LB , Havlir DV , Ayieko J , Mwangwa F , Owaraganise A , Kwarisiima D , et al. High levels of retention in care with streamlined care and universal test and treat in East Africa. AIDS. 2016;30(18):2855–2864. 27603290 10.1097/QAD.0000000000001250 PMC5332142

[92] Cassidy T , Cornell M , Runeyi P , Dutyulwa T , Kilani C , Duran LT , et al. Attrition from HIV care among youth initiating ART in youth‐only clinics compared with general primary healthcare clinics in Khayelitsha, South Africa: a matched propensity score analysis. J Int AIDS Soc. 2022;25(1):1–12. 10.1002/jia2.25854 PMC8789247 35077610

[93] Chanie ES , Tesgera Beshah D , Ayele AD . Incidence and predictors of attrition among children on antiretroviral therapy at University of Gondar Comprehensive Specialized Hospital, Northwest Ethiopia, 2019: retrospective follow‐up study. SAGE Open Med. 2022;10:1–12. 10.1177/20503121221077843 PMC8841924 35173969

[94] Charles J , Exavery A , Ally A , Mseya R , Mbwambo T , Barankena A , et al. Rates and determinants of retention on ART among orphans and vulnerable children living with HIV in Tanzania. Front Public Health. 2022;10:934412. 35968450 10.3389/fpubh.2022.934412 PMC9366305

[95] Ciccacci F , Ismael F , Chume V , Ruth L , Mbula P , Orlando S , et al. Enhancing retention in care in HIV‐infected adolescents during COVID‐19 in Mozambique: results from the DREAM program. Int J Adolesc Med Health. 2023;35(2):227–231. 36708359 10.1515/ijamh-2022-0107

[96] Ciccacci F , Orlando S , Sagno JB , Kamponda M , Gondwe J , Lunghi R , et al. Evaluation of nutritional conditions, haemoglobin levels, retention in care and viral suppression in a cohort of HIV‐infected Malawian adolescents undergoing a one‐year tailored intervention within the diseases relief through excellence and advanced means. South Afr J Child Health. 2020;14(4):228.

[97] Dorward J , Sookrajh Y , Gate K , Khubone T , Mtshaka N , Mlisana K , et al. HIV treatment outcomes among people with initiation CD4 counts >500 cells/µL after implementation of Treat All in South African public clinics: a retrospective cohort study. J Int AIDS Soc. 2020;23(4):e25479. 32319203 10.1002/jia2.25479 PMC7174836

[98] Dunlop EM , Darougar S , Treharne JD . Epidemiology of infection by serotypes D to K of chlamydia trachomatis. Br J Vener Dis. 1980;56(3):163–168. 7427689 10.1136/sti.56.3.163 PMC1045760

[99] Dzangare J , Takarinda KC , Harries AD , Tayler‐Smith K , Mhangara M , Apollo TM , et al. HIV testing uptake and retention in care of HIV‐infected pregnant and breastfeeding women initiated on ‘Option B+’ in rural Zimbabwe. Trop Med Int Health. 2016;21(2):202–209. 26555353 10.1111/tmi.12637

[100] Haghighat R , Toska E , Cluver L , Gulaid L , Mark D , Bains A . Transition pathways out of pediatric care and associated HIV outcomes for adolescents living with HIV in South Africa. J Acquir Immune Defic Syndr. 2019;82(2):166–174. 31335586 10.1097/QAI.0000000000002125 PMC6749967

[101] Hibstie YT , Kibret GD , Talie A , Temesgen B , Melkamu MW , Alebel A . Nearly one in every six HIV‐infected children lost from ART follow‐up at Debre Markos Referral Hospital, Northwest Ethiopia: a 14‐year retrospective follow‐up study. PLoS One. 2020;15:e0239013. 32931502 10.1371/journal.pone.0239013 PMC7491726

[102] Iyun V , Technau KG , Vinikoor M , Yotebieng M , Vreeman R , Abuogi L , et al. Variations in the characteristics and outcomes of children living with HIV following universal ART in sub‐Saharan Africa (2006–17): a retrospective cohort study. Lancet HIV. 2021;8(6):e353–e362. 33932330 10.1016/S2352-3018(21)00004-7 PMC8178242

[103] Iyun V , Technau KG , Eley B , Rabie H , Boulle A , Fatti G , et al. Earlier antiretroviral therapy initiation and decreasing mortality among HIV‐infected infants initiating antiretroviral therapy within 3 months of age in South Africa, 2006–2017. Pediatr Infect Dis J. 2020;39(2):127–133. 31725119 10.1097/INF.0000000000002516 PMC7073445

[104] Kerschberger B , Schomaker M , Jobanputra K , Kabore SM , Teck R , Mabhena E , et al. HIV programmatic outcomes following implementation of the ‘Treat‐All’ policy in a public sector setting in Eswatini: a prospective cohort study. J Int AIDS Soc. 2020;23(3):e25458. 32128964 10.1002/jia2.25458 PMC7054447

[105] Kose J , Tiam A , Siamba S , Lenz C , Okoth E , Wolters T , et al. Clinical outcomes among adolescents living with HIV in Kenya following initiation on antiretroviral treatment. PLOS Glob Public Health. 2022;2(2):e0000094. 36962291 10.1371/journal.pgph.0000094 PMC10022018

[106] Mengistu ST , Ghebremeskel GG , Achila OO , Abrehe MB , Tewelde SF , Idris MM , et al. Prevalence and factors associated with pediatric HIV therapy failure in a tertiary hospital in Asmara, Eritrea: a 15‐year retrospective cohort study. PLoS One. 2023;18(3):e0282642. 36893200 10.1371/journal.pone.0282642 PMC9997912

[107] Mengistu ST , Ghebremeskel GG , Rezene A , Idris MM , Tikue TG , Hamida ME , et al. Attrition and associated factors among children living with HIV at a tertiary hospital in Eritrea: a retrospective cohort analysis. BMJ Paediatr Open. 2022;6(1):e001414. 10.1136/bmjpo-2022-001414 PMC9252199 36053603

[108] Menshw Snr T , Birhanu S , Gebremaryam T , Yismaw W , Endalamaw A . Incidence and predictors of loss to follow‐up among children attending ART clinics in northeast Ethiopia: a retrospective cohort study. HIV/AIDS. 2021;13:801–812. 10.2147/HIV.S320601 PMC8364847 34408500

[109] Millar JR , Bengu N , Fillis R , Sprenger K , Ntlantsana V , Vieira VA , et al. HIGH‐FREQUENCY failure of combination antiretroviral therapy in paediatric HIV infection is associated with unmet maternal needs causing maternal NON‐ADHERENCE. EClinicalMedicine. 2020;22:100344. 32510047 10.1016/j.eclinm.2020.100344 PMC7264978

[110] Munyayi FK , van Wyk BE . Determinants and rates of retention in HIV care among adolescents receiving antiretroviral therapy in Windhoek, Namibia: a baseline cohort analysis. BMC Public Health. 2023;23(1):458. 36890540 10.1186/s12889-023-15356-w PMC9994767

[111] Munyayi FK , van Wyk BE . The comparison of teen clubs vs. standard care on treatment outcomes for adolescents on antiretroviral therapy in Windhoek, Namibia. AIDS Res Treat. 2020;2020:8604276. 33178455 10.1155/2020/8604276 PMC7609153

[112] Mushy SE , Mtisi E , Mboggo E , Mkawe S , Yahya‐Malima KI , Ndega J , et al. Predictors of the observed high prevalence of loss to follow‐up in ART‐experienced adult PLHIV: a retrospective longitudinal cohort study in the Tanga Region, Tanzania. BMC Infect Dis. 2023;23(1):1–9. 36788523 10.1186/s12879-023-08063-9 PMC9926646

[113] Muwanguzi M , Lugobe HM , Ssemwanga E , Lule AP , Atwiine E , Kirabira V , et al. Retention in HIV care and associated factors among youths aged 15–24 years in rural southwestern Uganda. BMC Public Health. 2021;21(1):1489. 34332556 10.1186/s12889-021-11547-5 PMC8325848

[114] Nhampossa T , Fernandez S , Augusto O , Fuente‐Soro L , MacUluve SÓN , Bernardo E , et al. Discordant retention of HIV‐infected mothers and children: evidence for a family‐based approach from Southern Mozambique. Medicine (United States). 2020;99(32):E21410. 10.1097/MD.0000000000021410 PMC7593016 32769871

[115] Nimwesiga C , Taremwa IM , Nakanjako D , Nasuuna E . Factors associated with retention in HIV care among HIV‐positive adolescents in public antiretroviral therapy clinics in Ibanda district, rural south western Uganda. HIV/AIDS. 2023;15:71–81. 10.2147/HIV.S401611 PMC9994664 36910020

[116] Ntabanganyimana D , Rugema L , Omolo J , Nsekuye O , Malamba SS . Incidence and factors associated with being lost to follow‐up among people living with HIV and receiving antiretroviral therapy in Nyarugenge the central business district of Kigali city, Rwanda. PLoS One. 2022;17:e0275954. 36228004 10.1371/journal.pone.0275954 PMC9562217

[117] Nyakato P , Schomaker M , Fatti G , Tanser F , Euvrard J , Sipambo N , et al. Virologic non‐suppression and early loss to follow up among pregnant and non‐pregnant adolescents aged 15–19 years initiating antiretroviral therapy in South Africa: a retrospective cohort study. J Int AIDS Soc. 2022;25(1):1–10. 10.1002/jia2.25870 PMC8760609 35032096

[118] Ross J , Sinayobye JD , Yotebieng M , Hoover DR , Shi Q , Ribakare M , et al. Early outcomes after implementation of treat all in Rwanda: an interrupted time series study. J Int AIDS Soc. 2019;22(4):1–9. 10.1002/jia2.25279 PMC6468264 30993854

[119] Sifr Z , Ando T , Semeon W , Rike M , Ashami K . Level of attrition from antiretroviral therapy among human immune deficiency virus‐infected children: the cases of Sidama Zone, southern Ethiopia. HIV/AIDS. 2021;13:813–822. 10.2147/HIV.S317117 PMC8370599 34413684

[120] Sikhondze N , Mahomed OH . Retention of children under 18 months testing HIV positive in care in Swaziland: a retrospective study. Pan Afr Med J. 2017;28:1–6. 10.11604/pamj.2017.28.316.13857 PMC5927566 29721146

[121] Tesha ED , Kishimba R , Njau P , Revocutus B , Mmbaga E . Predictors of loss to follow up from antiretroviral therapy among adolescents with HIV/AIDS in Tanzania. PLoS One. 2022;17(7):e0268825. 35857796 10.1371/journal.pone.0268825 PMC9299289

[122] Tsondai PR , Sohn AH , Phiri S , Sikombe K , Sawry S , Chimbetete C , et al. Characterizing the double‐sided cascade of care for adolescents living with HIV transitioning to adulthood across Southern Africa. J Int AIDS Soc. 2020;23(1):1–10. 10.1002/jia2.25447 PMC6992508 32003159

[123] Tweya H , Feldacker C , Kiruthu‐Kamamia C , Billion L , Gumulira J , Nhlema A , et al. Virologic failure and switch to second‐line antiretroviral therapy in children with HIV in Lilongwe, Malawi: an observational cohort study. Trans R Soc Trop Med Hyg. 2020;114(1):31–7. 31713619 10.1093/trstmh/trz087

[124] Van Liere GAFS , Lilian R , Dunlop J , Tait C , Rees K , Mabitsi M , et al. High rate of loss to follow‐up and virological non‐suppression in HIV‐infected children on antiretroviral therapy highlights the need to improve quality of care in South Africa. Epidemiol Infect. 2021;149:1–8. 10.1017/S0950268821000637 PMC8080219 33745490

[125] Zingoni ZM , Chirwa T , Todd J , Musenge E . Competing risk of mortality on loss to follow‐up outcome among patients with HIV on ART: a retrospective cohort study from the Zimbabwe national ART programme. BMJ Open. 2020;10(10):1–15. 10.1136/bmjopen-2019-036136 PMC7539573 33028546

[126] Ally A , Exavery A , Charles J , Kikoyo L , Mseya R , Barankena A , et al. Determinants of viral load suppression among orphaned and vulnerable children living with HIV on ART in Tanzania. Front Public Health. 2023;11:1076614. 37006553 10.3389/fpubh.2023.1076614 PMC10065403

[127] Amour M , Sangeda RZ , Kidenya B , Balandya E , Mmbaga BT , Machumi L , et al. Adherence to antiretroviral therapy by medication possession ratio and virological suppression among adolescents and young adults living with HIV in Dar es Salaam, Tanzania. Trop Med Infect Dis. 2022;7(4):1–10. 10.3390/tropicalmed7040052 PMC9028327 35448827

[128] Bayleyegn B , Kifle ZD , Geremew D . Virological failure and associated factors among children receiving anti‐retroviral therapy, Northwest Ethiopia. PLoS One. 2021;16(9):e0257204. 34506553 10.1371/journal.pone.0257204 PMC8432779

[129] Berihun H , Bazie GW , Beyene A , Zewdie A , Kebede N . Viral suppression and associated factors among children tested for HIV viral load at Amhara Public Health Institute, Dessie Branch, Ethiopia: a cross‐sectional study. BMJ Open. 2023;13(1):e068792. 10.1136/bmjopen-2022-068792 PMC9890760 36720566

[130] Bitwale NZ , Mnzava DP , Kimaro FD , Jacob T , Mpondo BCT , Jumanne S . Prevalence and factors associated with virological treatment failure among children and adolescents on antiretroviral therapy attending HIV/AIDS care and treatment clinics in Dodoma Municipality, Central Tanzania. J Pediatr Infect Dis Soc. 2021;10(2):131–140. 10.1093/jpids/piaa030 32463083

[131] Brathwaite R , Ssewamala FM , Neilands TB , Okumu M , Mutumba M , Damulira C , et al. Predicting the individualized risk of poor adherence to ART medication among adolescents living with HIV in Uganda: the Suubi+Adherence study. J Int AIDS Soc. 2021;24(6):1–10. 10.1002/jia2.25756 PMC8188571 34105865

[132] Cluver LD , Zhou S , Orkin M , Rudgard W , Meinck F , Langwenya N , et al. Impacts of intimate partner violence and sexual abuse on antiretroviral adherence among adolescents living with HIV in South Africa. AIDS. 2023;37(3):503–511. 36695360 10.1097/QAD.0000000000003440 PMC9894135

[133] Cluver L , Shenderovich Y , Toska E , Rudgard WE , Zhou S , Orkin M , et al. Clinic and care: associations with adolescent antiretroviral therapy adherence in a prospective cohort in South Africa. AIDS. 2021;35(8):1263–1271. 33730747 10.1097/QAD.0000000000002882 PMC8183481

[134] Crowley T , van der Merwe A , Kidd M , Skinner D . Adolescent human immunodeficiency virus self‐management: associations with treatment adherence, viral suppression, sexual risk behaviours and health‐related quality of life. South Afr J HIV Med. 2020;21(1):1–11. 10.4102/sajhivmed.v21i1.1054 PMC7203195 32391177

[135] Desta AA , Kidane KM , Woldegebriel AG , Ajemu KF , Berhe AA , Zgita DN , et al. Level of adherence and associated factors among HIV‐infected patients on antiretroviral therapy in Northern Ethiopia: retrospective analysis. Patient Preference Adherence. 2020;14:1585–1594. 32943850 10.2147/PPA.S268395 PMC7481295

[136] Edun O , Shenderovich Y , Zhou S , Toska E , Okell L , Eaton JW , et al. Predictors and consequences of HIV status disclosure to adolescents living with HIV in Eastern Cape, South Africa: a prospective cohort study. J Int AIDS Soc. 2022;25(5):e25910. 35543100 10.1002/jia2.25910 PMC9092159

[137] Gemechu J , Gebremichael B , Tesfaye T , Seyum A , Erkalo D . Predictors of mortality among TB‐HIV co‐infected children attending anti‐retroviral therapy clinics of selected public hospitals in southern, Ethiopia: retrospective cohort study. Arch Public Health. 2022;80(1):11. 34983618 10.1186/s13690-021-00713-1 PMC8728901

[138] Gitahi‐Kamau N , Wahome S , Bukusi EA , Ngure K . Determinants of antiretroviral therapy adherence among older adolescents living with HIV in Kenya during the transition to adult care; an observational study. J AIDS HIV Res. 2020;12(2):24–33. 34540322 10.5897/jahr2020.0513 PMC8445519

[139] Jackson C , Rehman AM , McHugh G , Gonzalez‐Martinez C , Ngwira LG , Bandason T , et al. Risk factors for sustained virological non‐suppression among children and adolescents living with HIV in Zimbabwe and Malawi: a secondary data analysis. BMC Pediatr. 2022;22(1):340. 35690762 10.1186/s12887-022-03400-4 PMC9188224

[140] Kabogo J , Muniu E , Wamunyokoli F , Musoke R , Songok E . Evidence of reduced treatment adherence among HIV infected paediatric and adolescent populations in Nairobi at the onset of the UNAIDS Universal Test and Treat Program. BMC Res Notes. 2018;11(1):134. 29452597 10.1186/s13104-018-3205-0 PMC5816400

[141] Kairania R , Onyango‐Ouma W , Ondicho TG , Kigozi G . HIV status disclosure and antiretroviral therapy adherence among children in Masaka region, Uganda. Afr J AIDS Res. 2022;21(3):251–260. 36111384 10.2989/16085906.2022.2060843

[142] Madiba S , Mohlabane N . Attendance of psychosocial teen clubs and self‐reported antiretroviral medication adherence: a cross section study of adolescents with perinatal HIV in the Kingdom of Lesotho. AIMS Public Health. 2021;8(3):541–552. 34395704 10.3934/publichealth.2021044 PMC8334641

[143] Mageda K , Kulemba K , Olomi W , Kapologwe N , Katalambula L , Petrucka P . Determinants of nonsuppression of HIV viral load among children receiving antiretroviral therapy in the Simiyu region: a cross‐sectional study. AIDS Res Ther. 2023;20(1):1–9. 37055786 10.1186/s12981-023-00515-1 PMC10099818

[144] Martelli G , Antonucci R , Mukurasi A , Zepherine H , Nöstlinger C . Adherence to antiretroviral treatment among children and adolescents in Tanzania: comparison between pill count and viral load outcomes in a rural context of Mwanza region. PLoS One. 2019;14(3):e0214014. 30897131 10.1371/journal.pone.0214014 PMC6428300

[145] McBride K , Parent J , Mmanga K , Chivwala M , Nyirenda MH , Schooley A , et al. ART adherence among Malawian youth enrolled in teen clubs: a retrospective chart review. AIDS Behav. 2019;23(9):2629–2633. 31292826 10.1007/s10461-019-02580-y

[146] Mugo C , Kohler P , Kumar M , Badia J , Kibugi J , Wamalwa DC , et al. Effect of HIV stigma on depressive symptoms, treatment adherence, and viral suppression among youth with HIV. AIDS. 2023;37(5):813–821. 36728652 10.1097/QAD.0000000000003473 PMC10023427

[147] Mussa FM , Massawe HP , Bhalloo H , Moledina S , Assenga E . Magnitude and associated factors of antiretroviral therapy adherence among children attending HIV care and treatment clinics in Dar es Salaam, Tanzania. PLoS One. 2022;17:e0275420. 36178915 10.1371/journal.pone.0275420 PMC9524636

[148] Mwangi A , van Wyk B . Factors associated with viral suppression among adolescents on antiretroviral therapy in Homa Bay County, Kenya: a retrospective cross‐sectional study. HIV/AIDS. 2021;13:1111–1118. 10.2147/HIV.S345731 PMC8713714 34992469

[149] Nabunya P , Samuel K , Ssewamala FM . The effect of family support on self‐reported adherence to ART among adolescents perinatally infected with HIV in Uganda: a mediation analysis. J Adolescence. 2023;95:834–843. 10.1002/jad.12157 PMC10257769 36810778

[150] Natukunda J , Kirabira P , Ong KIC , Shibanuma A , Jimba M . Virologic failure in HIV‐positive adolescents with perfect adherence in Uganda: a cross‐sectional study. Trop Med Health. 2019;47:8. 30679930 10.1186/s41182-019-0135-z PMC6337787

[151] Nguyen N , Lovero KL , Falcao J , Brittain K , Zerbe A , Wilson IB , et al. Mental health and ART adherence among adolescents living with HIV in Mozambique. AIDS Care. 2023;35(2):182–190. 35277102 10.1080/09540121.2022.2032574 PMC10243515

[152] Osman FT , Yizengaw MA . Virological failure and associated risk factors among HIV/AIDS pediatric patients at the ART clinic of Jimma University Medical Center, southwest Ethiopia. Open AIDS J. 2020;14(1):61–67.

[153] Ssanyu JN , Nakafeero M , Nuwaha F . Multi‐measure assessment of adherence to antiretroviral therapy among children under five years living with HIV in Jinja, Uganda. BMC Public Health. 2020;20(1):1319. 32867724 10.1186/s12889-020-09430-w PMC7457490

[154] Tadesse BT , Foster BA , Latour E , Lim JY , Jerene D , Ruff A , et al. Predictors of virologic failure among a cohort of HIV‐infected children in southern Ethiopia. Pediatr Infect Dis J. 2021;40(1):60–65. 32925538 10.1097/INF.0000000000002898

[155] Tekliye E , Alemayehu T , Bacha T . Clinical, immunologic and virologic outcomes of children and adolescents receiving second line anti‐retroviral therapy in two referral hospitals in Addis Ababa, Ethiopia. PLoS One. 2021;16(3):e0249085. 33784335 10.1371/journal.pone.0249085 PMC8009351

[156] Tesfahunegn TB , Berhe N , Abraha TH , Hintsa S , Yohanes G , Desta K , et al. Adherence to antiretroviral therapy and associated factors among HIV‐infected children in public health institutions of Adwa, Axum, and Shire Towns of Tigray, Northern Ethiopia: a cross‐sectional study. HIV AIDS (Auckl). 2023;15:217–24. 37163177 10.2147/HIV.S282938 PMC10164383

[157] Tong PD , Atuhairwe C , Taremwa IM . Differential self‐reported determinants to antiretroviral therapy adherence: findings from caregivers of children under five years living with human immunodeficiency virus infection attending Al‐Sabah hospital, south Sudan. HIV/AIDS. 2020;12:175–186. 10.2147/HIV.S248057 PMC7244449 32547247

[158] Umar E , Levy JA , Bailey RC , Donenberg G , Hershow RC , Mackesy‐Amiti ME . Virological non‐suppression and its correlates among adolescents and young people living with HIV in southern Malawi. AIDS Behav. 2019;23(2):513–522. 30132172 10.1007/s10461-018-2255-6 PMC8896833

[159] Weldemariam SA , Dagnew Z , Tafere Y , Bereka TM , Bitewa YB . Time to death among HIV‐infected under‐five children after initiation of anti‐retroviral therapy and its predictors in Oromiya liyu zone, Amhara region, Ethiopia: a retrospective cohort study. BMC Pediatr. 2022;22(1):5. 34980032 10.1186/s12887-021-03072-6 PMC8722209

[160] Yihun BA , Kibret GD , Leshargie CT . Incidence and predictors of treatment failure among children on first‐line antiretroviral therapy in Amhara Region Referral Hospitals, northwest Ethiopia 2018: a retrospective study. PLoS One. 2019;14(5):e0215300. 31042743 10.1371/journal.pone.0215300 PMC6494040

[161] Zhou S , Cluver L , Shenderovich Y , Toska E . Uncovering ART adherence inconsistencies: an assessment of sustained adherence among adolescents in South Africa. J Int AIDS Soc. 2021;24(10):1–6. 10.1002/jia2.25832 PMC8552454 34708912

[162] Alibi M , Mwapasa V , Ngwalangwa F . Retrospective cohort study comparing antiretroviral treatment outcomes among adolescents in teen clubs and standard care clinics: Blantyre, Malawi. J Int Assoc Provid AIDS Care. 2023;22:1–7. 10.1177/23259582231172340 PMC10196531 37194291

[163] Amour MA , Shayo GA , Matee MM , Machumi L , Rugarabamu A , Aris EA , et al. Predictors of mortality among adolescents and young adults living with HIV on antiretroviral therapy in Dar es Salaam, Tanzania: a retrospective cohort study. J Int AIDS Soc. 2022;25(2):1–8. 10.1002/jia2.25886 PMC8863353 35192739

[164] Bacha JM , Dlamini S , Anabwani F , Gwimile J , Kanywa JB , Farirai J , et al. Realizing the promise of dolutegravir in effectively treating children and adolescents living with HIV in real‐world settings in 6 countries in eastern and southern Africa. Pediatr Infect Dis J. 2023;42:576–581. 36795586 10.1097/INF.0000000000003878 PMC10259212

[165] Bermudez LG , Ssewamala FM , Neilands TB , Lu L , Jennings L , Nakigozi G , et al. Does economic strengthening improve viral suppression among adolescents living with HIV? Results from a cluster randomized trial in Uganda. AIDS Behav. 2018;22(11):3763–3772. 29846836 10.1007/s10461-018-2173-7 PMC6204092

[166] Chouraya C , Ashburn K , Khumalo P , Mpango L , Mthethwa N , Machekano R , et al. Association of antiretroviral drug regimen with viral suppression in HIV‐positive children on antiretroviral therapy in Eswatini. Pediatr Infect Dis J. 2019;38(8):835–839. 31033912 10.1097/INF.0000000000002347

[167] Desta AA , Woldearegay TW , Futwi N , Gebrehiwot GT , Gebru GG , Berhe AA , et al. HIV virological non‐suppression and factors associated with non‐suppression among adolescents and adults on antiretroviral therapy in northern Ethiopia: a retrospective study. BMC Infect Dis. 2020;20(1):1585–1593. 10.1186/s12879-019-4732-6 PMC6941313 31898535

[168] de Waal R , Rabie H , Technau KG , Eley B , Sipambo N , Cotton M , et al. Abacavir safety and effectiveness in young infants with HIV in South African observational cohorts. Antivir Ther. 2023;28(2):1–5. 10.1177/13596535231168480 PMC10961679 37038365

[169] Elashi BAY , Van Wyk BE . Factors associated with viral suppression among adolescents on antiretroviral therapy in Free State province, South Africa. South Afr J HIV Med. 2022;23(1):1–5. 10.4102/sajhivmed.v23i1.1356 PMC9257832 35923610

[170] Endalamaw Alamneh D , Shiferaw MB , Demissie MG , Emiru MA , Kassie TZ , Lakew KE , et al. Virological outcomes among pregnant women receiving antiretroviral treatment in the Amhara Region, North West Ethiopia. HIV/AIDS. 2023;15:209–216. 10.2147/HIV.S389506 PMC10163878 37159581

[171] Fenta DA , Wube TB , Nuru MM . Long‐term immunological and virological outcomes in children receiving highly active antiretroviral therapy at Hawassa University College of Medicine and Health Sciences, Southern Ethiopia. J Immunol Res. 2021;2021:1–9. 10.1155/2021/2498025 PMC8053046 33928167

[172] Filiatreau LM , Pettifor A , Edwards JK , Masilela N , Twine R , Xavier Gómez‐Olivé F , et al. Associations between key psychosocial stressors and viral suppression and retention in care among youth with HIV in rural South Africa. AIDS Behav. 2021;25(8):2358–2368. 33624194 10.1007/s10461-021-03198-9 PMC8222008

[173] Gordon TP , Talbert M , Mugisha MK , Herbert AE . Factors associated with HIV viral suppression among adolescents in Kabale district, South Western Uganda. PLoS One. 2022;17(8):e0270855. 35980902 10.1371/journal.pone.0270855 PMC9387807

[174] Hansoti B , Stead D , Eisenberg A , Mvandaba N , Mwinnyaa G , Patel EU , et al. A window into the HIV epidemic from a South African Emergency Department. AIDS Res Hum Retroviruses. 2019;35(2):139–144. 30215268 10.1089/aid.2018.0127 PMC6360397

[175] Humphrey JM , Genberg BL , Keter A , Musick B , Apondi E , Gardner A , et al. Viral suppression among children and their caregivers living with HIV in western Kenya. J Int AIDS Soc. 2019;22(4):1–10. 10.1002/jia2.25272 PMC6462809 30983148

[176] Lain MG , Vaz P , Sanna M , Ismael N , Chicumbe S , Simione TB , et al. Viral response among early treated HIV perinatally infected infants: description of a cohort in southern Mozambique. Healthcare (Basel). 2022;10(11):1–15. 10.3390/healthcare10112156 PMC9691232 36360495

[177] Leshargie CT , Demant D , Burrowes S , Frawley J . Incidence and predictors of mortality among adolescents on antiretroviral therapy in Amhara Region, Ethiopia: a retrospective cohort analysis. BMJ Open. 2022;12(11):e063879. 10.1136/bmjopen-2022-063879 PMC9664312 36351711

[178] Levy M , Duffy M , Pearson J , Akuno J , Oduong S , Yemaneberhan A , et al. Health and social outcomes of HIV‐vulnerable and HIV‐positive pregnant and post‐partum adolescents and infants enrolled in a home visiting team programme in Kenya. Trop Med Int Health. 2021;26(6):640–648. 33662176 10.1111/tmi.13568 PMC9291167

[179] Maena J , Banke‐Thomas A , Mukiza N , Kuteesa CN , Kakumba RM , Kataike H , et al. Determinants of viral load non‐suppression among adolescents in Mbale District, eastern rural Uganda. AIDS Res Ther. 2021;18(1):91. 34863196 10.1186/s12981-021-00408-1 PMC8642852

[180] Mapangisana T , Machekano R , Kouamou V , Maposhere C , McCarty K , Mudzana M , et al. Viral load care of HIV‐1 infected children and adolescents: a longitudinal study in rural Zimbabwe. PLoS One. 2021;16(1):e0245085. 33444325 10.1371/journal.pone.0245085 PMC7808638

[181] Masaba R , Woelk G , Siamba S , Ndimbii J , Ouma M , Khaoya J , et al. Antiretroviral treatment failure and associated factors among people living with HIV on therapy in Homa Bay, Kenya: a retrospective study. PLOS Glob Public Health. 2023;3(3):e0001007. 36962996 10.1371/journal.pgph.0001007 PMC10021395

[182] Mathamo A , Naidoo KL , Dorward J , Archary T , Bottomley C , Archary M . COVID‐19 and HIV viral load suppression in children and adolescents in Durban, South Africa. South Afr J HIV Med. 2022;23(1):1–7. 10.4102/sajhivmed.v23i1.1424 PMC9772656 36575700

[183] Mburu M , Guzé MA , Ong'wen P , Okoko N , Moghadassi M , Cohen CR , et al. Evaluating the effectiveness of the HIV adolescent package of care (APOC) training on viral load suppression in Kenya. Public Health. 2019;173:146–149. 31310874 10.1016/j.puhe.2019.05.026

[184] Mburugu P , Muiruri P , Opiyo N , Simba J , Adunda J , Kawira R , et al. Antiretroviral therapy outcomes among adolescents and young adults in a tertiary hospital in Kenya. Afr Health Sci. 2021;21:1–7. 10.4314/ahs.v21i1.2S PMC8367303 34447417

[185] Merrill KG , Campbell JC , Decker MR , McGready J , Burke VM , Mwansa JK , et al. Past‐year violence victimization is associated with viral load failure among HIV‐positive adolescents and young adults. AIDS Behav. 2021;25(5):1373–1383. 32761474 10.1007/s10461-020-02958-3 PMC8052241

[186] Mhlanga TT , Jacobs BKM , Decroo T , Govere E , Bara H , Chonzi P , et al. Virological outcomes and risk factors for non‐suppression for routine and repeat viral load testing after enhanced adherence counselling during viral load testing scale‐up in Zimbabwe: analytic cross‐sectional study using laboratory data from 2014 to 2018. AIDS Res Ther. 2022;19(1):1–13. 35810317 10.1186/s12981-022-00458-z PMC9270749

[187] Moyo S , Ncube RT , Shewade HD , Ngwenya S , Ndebele W , Takarinda KC , et al. Children and adolescents on anti‐retroviral therapy in Bulawayo, Zimbabwe: how many are virally suppressed by month six? F1000Res. 2020;9:191. 32399206 10.12688/f1000research.22744.1 PMC7194453

[188] Munyayi FK , van Wyk B . Closing the HIV treatment gap for adolescents in Windhoek, Namibia: a retrospective analysis of predictors of viral non‐suppression. Int J Environ Res Public Health. 2022;19(22):14710. 36429431 10.3390/ijerph192214710 PMC9690371

[189] Nasuuna E , Kigozi J , Babirye L , Muganzi A , Sewankambo NK , Nakanjako D . Low HIV viral suppression rates following the intensive adherence counseling (IAC) program for children and adolescents with viral failure in public health facilities in Uganda. BMC Public Health. 2018;18(1):1048. 30134880 10.1186/s12889-018-5964-x PMC6103875

[190] Nega J , Taye S , Million Y , Rodrigo C , Eshetie S . Antiretroviral treatment failure and associated factors among HIV patients on first‐line antiretroviral treatment in Sekota, northeast Ethiopia. AIDS Res Ther. 2020;17(1):1–9. 32650796 10.1186/s12981-020-00294-z PMC7350666

[191] Negash H , Welay M , Legese H , Adhanom G , Mardu F , Tesfay K , et al. Increased virological failure and determinants among HIV patients on highly active retroviral therapy in Adigrat General Hospital, northern Ethiopia, 2019: hospital‐based cross‐sectional study. Infect Drug Resist. 2020;13:1863–1872. 32606835 10.2147/IDR.S251619 PMC7308120

[192] Ng'ambi WF , Estill J , Jahn A , Orel E , Chimpandule T , Nyirenda R , et al. Factors associated with HIV viral suppression among children and adults receiving antiretroviral therapy in Malawi in 2021: evidence from the Laboratory Management Information System. Trop Med Int Health. 2022;27(7):639–646. 35622358 10.1111/tmi.13782

[193] Ngandu NK , Lombard CJ , Mbira TE , Puren A , Waitt C , Prendergast AJ , et al. HIV viral load non‐suppression and associated factors among pregnant and postpartum women in rural northeastern South Africa: a cross‐sectional survey. BMJ Open. 2022;12(3):e058347. 10.1136/bmjopen-2021-058347 PMC8915310 35273061

[194] Njuguna I , Neary J , Mburu C , Black D , Beima‐Sofie K , Wagner AD , et al. Clinic‐level and individual‐level factors that influence HIV viral suppression in adolescents and young adults: a national survey in Kenya. AIDS. 2020;34(7):1065–1074. 32287060 10.1097/QAD.0000000000002538 PMC7274775

[195] Nuttall J , Pillay V . Characteristics and early outcomes of children and adolescents treated with darunavir/ritonavir‐, raltegravir‐ or etravirine‐containing antiretroviral therapy in the Western Cape Province of South Africa. S Afr Med J. 2018;108(2):105. 29429441 10.7196/SAMJ.2017.v108i2.12573

[196] Nzivo MM , Waruhiu CN , Kang'Ethe JM , Budambula NLM . HIV virologic failure among patients with persistent low‐level viremia in Nairobi, Kenya: it is time to review the >1000 virologic failure threshold. Biomed Res Int. 2023;2023:1–10. 10.1155/2023/8961372 PMC10159743 37152588

[197] Okonji EF , van Wyk B , Mukumbang FC , Hughes GD . Determinants of viral suppression among adolescents on antiretroviral treatment in Ehlanzeni district, South Africa: a cross‐sectional analysis. AIDS Res Ther. 2021;18(1):1–9. 34627300 10.1186/s12981-021-00391-7 PMC8501534

[198] Onyango B , Mokaya R , Wasianga J , Wao H , Achwoka D , Onyango N , et al. Factors associated with viral load suppression among orphans and vulnerable children and adolescents living with HIV in Kenya. PLOS Glob Public Health. 2023;3(3):e0000794. 36963026 10.1371/journal.pgph.0000794 PMC10035747

[199] Philbert D , Msovela J , Burengelo D , Hassan FE , Kitinya C , Soka G , et al. HIV treatment outcomes and their associated factors among adolescents and youth living with HIV in Tanzania. Tanzania J Health Res. 2023;24(1):1–15.

[200] Porter JD , Porter MNM , Du Plessis M . Not yet 90‐90‐90: a quality improvement approach to human immunodeficiency virus viral suppression in paediatric patients in the rural Eastern Cape, South Africa . S Afr Fam Pract (2004). 2020;62(1):1–6. 10.4102/safp.v62i1.5169 PMC7674377 33179951

[201] Ravishankar M , Dallah I , Mathews M , Bositis CM , Mwenechanya M , Kalungwana‐Mambwe L , et al. Clinical characteristics and outcomes after new‐onset seizure among Zambian children with HIV during the antiretroviral therapy era. Epilepsia Open. 2022;7(2):315–324. 35305291 10.1002/epi4.12595 PMC9159241

[202] Rugemalila J , Kamori D , Kunambi P , Mizinduko M , Sabasaba A , Masoud S , et al. HIV virologic response, patterns of drug resistance mutations and correlates among adolescents and young adults: a cross‐sectional study in Tanzania. PLoS One. 2023;18:e0281528. 36821538 10.1371/journal.pone.0281528 PMC9949668

[203] Schrubbe LA , Stöckl H , Hatcher AM , Marston M , Kuchukhidze S , Calvert C . Prevalence and risk factors of unsuppressed viral load among pregnant and breastfeeding women in sub‐Saharan Africa: analysis from population‐based surveys. AIDS. 2023;37(4):659–669. 36511117 10.1097/QAD.0000000000003459

[204] Sher R , Dlamini S , Muloiwa R . Patterns of detectable viral load in a cohort of HIV‐positive adolescents on antiretroviral therapy in South Africa. J Int AIDS Soc. 2020;23(3):1–6. 10.1002/jia2.25474 PMC7076279 32180367

[205] Shiau S , Strehlau R , Shen Y , He Y , Patel F , Burke M , et al. Virologic response to very early HIV treatment in neonates. J Clin Med. 2021;10(10):2074. 34066021 10.3390/jcm10102074 PMC8151270

[206] Tadesse BT , Chala A , Mukonzo J , Chaka TE , Tadesse S , Makonnen E , et al. Rates and correlates of short term virologic response among treatment‐naïve HIV‐infected children initiating antiretroviral therapy in Ethiopia: a multi‐center prospective cohort study. Pathogens. 2019;8(4):161. 31554200 10.3390/pathogens8040161 PMC6963769

[207] Tanyi WN , Gachuno O , Odero T , Farquhar C , Kimosop D , Mayi A . Factors affecting adherence to antiretroviral therapy among children and adolescents living with HIV in the Mbita Sub‐County Hospital, Homa Bay‐Kenya. Afr Health Sci. 2021;21(Suppl):18–24. 34447419 10.4314/ahs.v21i1.4S PMC8367307

[208] Teasdale CA , Odondi J , Kidiga C , Choy M , Fayorsey R , Ngeno B , et al. Group antenatal care for improving retention of adolescent and young pregnant women living with HIV in Kenya. BMC Pregnancy Childbirth. 2022;22(1):1–10. 35291978 10.1186/s12884-022-04527-z PMC8925235

[209] Tsikhutsu I , Bii M , Dear N , Ganesan K , Kasembeli A , Sing'Oei V , et al. Prevalence and correlates of viral load suppression and human immunodeficiency virus (HIV) drug resistance among children and adolescents in South Rift Valley and Kisumu, Kenya. Clin Infect Dis. 2022;75(6):936–944. 35092424 10.1093/cid/ciac059 PMC9522406

[210] Vonasek BJ , Itaye T , Mhango J , Dean AL , Kazembe PN . Socioeconomic factors associated with virologic suppression in children and adolescents living with HIV in Lilongwe, Malawi. J Public Health (Germany). 2021;29(4):795–803.

[211] Wakooko P , Gavamukulya Y , Wandabwa JN . Viral load suppression and associated factors among HIV patients on antiretroviral treatment in Bulambuli district, eastern Uganda: a retrospective cohort study. Infect Dis: Res Treat. 2020;13:1–9. 10.1177/1178633720970632 PMC7656881 33223836

[212] Wilson K , Onyango A , Mugo C , Guthrie B , Slyker J , Richardson B , et al. Kenyan HIV clinics with youth‐friendly services and trained providers have a higher prevalence of viral suppression among adolescents and young adults: results from an observational study. J Assoc Nurses AIDS Care. 2022;33(1):45–53. 34939987 10.1097/JNC.0000000000000302 PMC10329499

[213] Zakaria HF , Raru TB , Hassen FA , Ayana GM , Merga BT , Debele GR , et al. Incidence and predictors of virological failure among adult HIV/AIDS patients on second‐line anti‐retroviral therapy, in selected public hospital of Addis Ababa, Ethiopia: retrospective follow‐up study. HIV/AIDS. 2022;14:319–329. 10.2147/HIV.S367677 PMC9275424 35836751

[214] Zijenah LS , Bandason T , Bara W , Chipiti MM , Katzenstein DA . Impact of Option B+Combination antiretroviral therapy on mother‐to‐child transmission of HIV‐1, maternal and infant virologic responses to combination antiretroviral therapy, and maternal and infant mortality rates: a 24‐month prospective follow‐up study. AIDS Patient Care STDs. 2022;36(4):145–152. 35438521 10.1089/apc.2021.0217 PMC9057887

[215] Stover J , Glaubius R , Kassanjee R , Dugdale CM . Updates to the Spectrum/AIM model for the UNAIDS 2020 HIV estimates. J Int AIDS Soc. 2021;24(Suppl 5):e25778. 34546648 10.1002/jia2.25778 PMC8454674

[216] Makurumidze R , Decroo T , Jacobs BKM , Rusakaniko S , Van Damme W , Lynen L , et al. Attrition one year after starting antiretroviral therapy before and after the programmatic implementation of HIV “Treat All” in sub‐Saharan Africa: a systematic review and meta‐analysis. BMC Infect Dis. 2023;23(1):558. 37641003 10.1186/s12879-023-08551-y PMC10463759

[217] Hlophe LD , Tamuzi JL , Shumba CS , Nyasulu PS . Barriers and facilitators to anti‐retroviral therapy adherence among adolescents aged 10 to 19 years living with HIV in sub‐Saharan Africa: a mixed‐methods systematic review and meta‐analysis. PLoS One. 2023;18(5):e0276411. 37200399 10.1371/journal.pone.0276411 PMC10194875

[218] Schlatter AF , Deathe AR , Vreeman RC . The need for pediatric formulations to treat children with HIV. AIDS Res Treat. 2016;2016:1654938. 27413548 10.1155/2016/1654938 PMC4927993

[219] Maskew M , Technau K , Davies MA , Vreeman R , Fox MP . Adolescent retention in HIV care within differentiated service‐delivery models in sub‐Saharan Africa. Lancet HIV. 2022;9(10):e726–e734. 36088915 10.1016/S2352-3018(22)00137-0 PMC9927242

[220] Casale M , Carlqvist A , Cluver L . Recent interventions to improve retention in HIV care and adherence to antiretroviral treatment among adolescents and youth: a systematic review. AIDS Patient Care STDs. 2019;33(6):237–252. 31166783 10.1089/apc.2018.0320 PMC6588099

[221] Okonkwo NE , Blum A , Viswasam N , Hahn E , Ryan S , Turpin G , et al. A systematic review of linkage‐to‐care and antiretroviral initiation implementation strategies in low‐ and middle‐income countries across sub‐Saharan Africa. AIDS Behav. 2022;26(7):2123–2134. 35088176 10.1007/s10461-021-03558-5 PMC9422958

[222] Vourli G , Katsarolis I , Pantazis N , Touloumi G . HIV continuum of care: expanding scope beyond a cross‐sectional view to include time analysis: a systematic review. BMC Public Health. 2021;21(1):1699. 34535096 10.1186/s12889-021-11747-z PMC8447660

