# Supplementary material for: Characterizing the HIV care continuum among children and adolescents with HIV in eastern and southern Africa in the era of “Universal Test and Treat”: A systematic review and meta‐analysis
Source: J Int AIDS Soc. 2025 Jun 13;28(6):e26526. doi: 10.1002/jia2.26526 (PMC12166130; doi:10.1002/jia2.26526)
Supplement: Supplementary file 1 — File S1. Additional tables and figures. Search criteria, summary tables of the systematic review and forest plots. [file JIA2-28-e26526-s001.docx]

Supplementary Files

[Supplementary Table 1. Search criteria for Embase and PubMed 2](#_Toc169181499)

[Supplementary Table 2. Calendar years of “Universal Test and Treat” (UTT) Implementation by country. 4](#_Toc169181500)

[Supplementary Table 3. Characteristics of studies included in the systematic review. 6](#_Toc169181501)

[Supplementary Figure 1. Forest plots of diagnosis and knowledge of HIV status, by children 0-14 and adolescents 15-19, in Eastern and Southern Africa. 18](#_Toc169181502)

[Supplementary Figure 2. Forest plots of on ART after diagnosis, by children 0-14 and adolescents 15-19, in Eastern and Southern Africa. 19](#_Toc169181503)

[Supplementary Figure 3. Forest plots of rapid ART initiation (<7 days after diagnosis), by children 0-14 and adolescents 15-19, in Eastern and Southern Africa. 20](#_Toc169181504)

[Supplementary Figure 4. Forest plots of retention in HIV care at 6 months, by children 0-14 and adolescents 15-19, in Eastern and Southern Africa. 21](#_Toc169181505)

[Supplementary Figure 5. Forest plots of retention in HIV care at 12 months, by children 0-14 and adolescents 15-19, in Eastern and Southern Africa.  22](#_Toc169181506)

[Supplementary Figure 6. Forest plots of retention in HIV care at 24 months, by children 0-14 and adolescents 15-19, in Eastern and Southern Africa.  23](#_Toc169181507)

[Supplementary Figure 7. Forest plots of adherent to antiretroviral therapy (self- or caregiver-reported), by children 0-14 and adolescents 15-19, in Eastern and Southern Africa.  24](#_Toc169181508)

[Supplementary Figure 8. Forest plots of adherent to antiretroviral therapy (health facility-reported), by children 0-14 and adolescents 15-19, in Eastern and Southern Africa.   25](#_Toc169181509)

[Supplementary Figure 9. Forest plots of having a viral load test after ART initiation, by children 0-14 and adolescents 15-19, in Eastern and Southern Africa.  26](#_Toc169181510)

[Supplementary Figure 10. Forest plots of having a suppressed viral load (<1,000 copies/ml), by children 0-14 and adolescents 15-19, in Eastern and Southern Africa. 28](#_Toc169181511)

# Supplementary Table 1. Search criteria for Embase and PubMed

Note: Each set of search terms (“concept”) was stitched together as follows:

[HIV] AND [Children and adolescents] AND ([HIV care continuum] or [Diagnosis] or [Antiretroviral therapy] or [Linkage to care] or [Retention in care] or [Viral load testing] or [Adherence]) AND [Countries in Eastern and Southern Africa] AND [Publication from 2010]

| **Concept** | **Embase Search Term** | **PubMed Search Term** |
| --- | --- | --- |
| HIV | ('human immunodeficiency virus'/exp OR hiv*:ab,ti,kw OR (('human immun*' NEAR/1 'deficien*' NEAR/1 'virus'):ab,ti,kw)) OR ('acquired immune deficiency syndrome'/exp OR (('acquired' NEAR/1 'immun*' NEAR/1 'deficien*' NEAR/1 'syndrome'):ab,ti,kw)) | HIV[MeSH Terms] OR "HIV infections"[MeSH Terms] OR AIDS[MeSH Terms] OR hiv[tw] OR ('human immun*'[tw] n1 'deficien*'[tw] n1 'virus' [tw]) OR ('acquired'[tw] n1 'immun*'[tw] n1 'deficien*'[tw] n1 'syndrome'[tw]) |
| Children and adolescents | ('child'/exp OR child:ab,ti,kw) OR  ('pediatrics'/exp OR pediatric*:ab,ti,kw OR paediatric*:ab,ti,kw) OR  ('adolescence'/exp OR adolesc*:ab,ti,kw OR teen*:ab,ti,kw) OR  ('young adult'/exp OR ('young*':ab,ti,kw AND 'adult*':ab,ti,kw)) | child[MeSH Terms] OR pediatrics[MeSH Terms] OR adolescent[MeSH Terms] OR young adult[MeSH Terms] OR child[tw] OR pediatric*[tw] OR paediatric*[tw] OR adolesc*[tw] OR teen*[tw] OR (young*[tw] AND adult*[tw]) |
| HIV care continuum | ('patient care'/exp OR (((continuum OR continuity OR cascade OR standard) NEAR/2 (care OR 'health service*')):ab,ti,kw)) OR (health care quality'/exp OR (('health' NEAR/2 'quality'):ab,ti,kw) OR (('standard' NEAR/2 'care'):ab,ti,kw)) OR  ('patient referral'/exp OR 'refer*':ab,ti,kw) OR ('health care access'/exp OR (('health*' NEAR/2 'access*'):ab,ti,kw)) OR ('consultation'/exp OR 'consult*':ab,ti,kw) | ("continuity of patient care"[MeSH Terms] OR "standard of care"[MeSH Terms] OR "referral and consultation"[MeSH Terms] OR "health services accessibility"[MeSH Terms] OR ((continuum[tw] OR continuity[tw] OR cascade[tw] OR standard[tw]) n2 (care[tw] OR 'health service*'[tw]))) OR ("quality of health care"[MeSH Terms] OR ('health'[tw] n2 'quality'[tw]) OR ('standard'[tw] n2 'care'[tw])) OR ('refer*'[tw] OR (health*[tw] n2 access*[tw]) OR consult*[tw]) |
| Diagnosis | 'hiv test'/exp OR (('hiv' NEAR/1 ('point of care' OR 'test*' OR 'diag*')):ab,ti,kw) | "HIV testing"[MeSH Terms] OR (hiv[tw] n1 ('point of care'[tw] OR 'test'[tw] OR 'diag'[tw])) |
| Antiretroviral therapy | 'antiretroviral therapy'/exp OR 'highly active antiretroviral therapy'/exp OR 'anti human immunodeficiency virus agent'/exp OR (((antiretrovir* OR 'anti retrovir*' OR 'anti-retrovir*') NEAR/1 (therap* OR treat* OR agent*)):ab,ti,kw) | "anti-retroviral agents"[MeSH Terms] OR "anti-HIV agents"[MeSH Terms] OR "antiretroviral therapy, highly active"[MeSH Terms] OR ((antiretrovir*[tw] OR 'anti retrovir*'[tw]) n1 (therap*[tw] OR treat*[tw] OR agent*[tw])) |
| Linkage to care | care:ab,ti,kw AND (link*:ab,ti,kw OR enrol*:ab,ti,kw OR consult*:ab,ti,kw OR access*:ab,ti,kw OR engag*:ab,ti,kw OR connect*:ab,ti,kw OR enter*:ab,ti,kw OR entry:ab,ti,kw OR entrance:ab,ti,kw OR initiat*:ab,ti,kw OR integra*:ab,ti,kw OR attend*:ab,ti,kw) OR ((treatment:ab,ti,kw OR therapy:ab,ti,kw) AND (rapid:ab,ti,kw OR 'same day':ab,ti,kw OR 'fast track':ab,ti,kw OR universal:ab,ti,kw OR 'test and treat':ab,ti,kw OR 'treat all':ab,ti,kw OR early:ab,ti,kw OR accelerat*:ab,ti,kw OR instant:ab,ti,kw OR prompt:ab,ti,kw OR fast:ab,ti,kw OR quick:ab,ti,kw OR expedit*:ab,ti,kw OR immediate:ab,ti,kw)) | care[tw] AND (link*[tw] OR enrol*[tw] OR consult*[tw] OR access*[tw] OR engag*[tw] OR connect*[tw] OR enter*[tw] OR entry[tw] OR entrance[tw] OR initiat*[tw] OR integra*[tw] OR attend*[tw]) OR ((treatment[tw] OR therapy[tw]) AND (rapid[tw] OR 'same day'[tw] OR 'fast track'[tw] OR universal[tw] OR 'test and treat'[tw] OR 'treat all'[tw] OR early[tw] OR accelerat*[tw] OR instant[tw] OR prompt[tw] OR fast[tw] OR quick[tw] OR expedit*[tw] OR immediate[tw])) |
| Retention in care | 'retention in care'/exp OR (care:ab,ti,kw AND ('retain':ab,ti,kw OR 'retention':ab,ti,kw)) | "Retention in care"[MeSH Terms] OR (care[tw] AND ('retain'[tw] OR 'retention'[tw])) |
| Viral load testing | ('viremia'/exp OR 'viral load testing'/exp OR 'second-line treatment'/exp) OR (((viral OR virus OR virologic*) NEAR/1 load NEAR/2 (undetect* OR monitor* OR test* OR fail* OR resist* OR suppress* OR success OR response OR level OR concentration OR sustain)):ab,ti,kw) | "Drug Resistance, Viral"[MeSH Terms] OR "anti-retroviral agents"[MeSH Terms] OR "anti-HIV agents"[MeSH Terms] OR "antiretroviral therapy, highly active"[MeSH Terms] OR "viremia" [MeSH Terms] OR "viral load"[MeSH Terms] OR ((viral[tw] OR virus[tw] OR virologic*[tw]) n1 load[tw] n2 (undetect*[tw] OR monitor*[tw] OR test*[tw] OR fail*[tw] OR resist*[tw] OR suppress*[tw] OR success[tw] OR response[tw] OR level[tw] OR concentration[tw] OR sustain[tw])) |
| Adherence | ('patient compliance'/exp OR adheren*:ab,ti,kw OR complian*:ab,ti,kw) | "medication adherence"[MeSH Terms] OR adheren*[tw] OR complian*[tw] |
| Countries in Eastern and Southern Africa | (angola:ab,ti,kw OR botswana:ab,ti,kw OR comoros:ab,ti,kw OR eritrea:ab,ti,kw OR eswatini:ab,ti,kw OR swaziland:ab,ti,kw OR ethiopia:ab,ti,kw OR kenya:ab,ti,kw OR lesotho:ab,ti,kw OR madagascar:ab,ti,kw OR malawi:ab,ti,kw OR mauritius:ab,ti,kw OR mozambique:ab,ti,kw OR namibia:ab,ti,kw OR rwanda:ab,ti,kw OR seychelles:ab,ti,kw OR 'south africa':ab,ti,kw OR 'south sudan':ab,ti,kw OR uganda:ab,ti,kw OR tanzania:ab,ti,kw OR zambia:ab,ti,kw OR zimbabwe:ab,ti,kw) OR ((east*:ab,ti,kw OR south*:ab,ti,kw) AND africa:ab,ti,kw) | (angola[tw] OR botswana[tw] OR comoros[tw] OR eritrea[tw] OR eswatini[tw] OR swaziland[tw] OR ethiopia[tw] OR kenya[tw] OR lesotho[tw] OR madagascar[tw] OR malawi[tw] OR mauritius[tw] OR mozambique[tw] OR namibia[tw] OR rwanda[tw] OR seychelles[tw] OR 'south africa'[tw] OR 'south sudan'[tw] OR uganda[tw] OR tanzania[tw] OR zambia[tw] OR zimbabwe[tw]) OR ("Africa, Eastern"[MeSH Terms] OR "Africa, Southern"[MeSH Terms] OR ((east*[tw] OR south*[tw]) AND africa[tw])) |
| Publication from 2010 | ("2010/01/01"[Date - Publication] : "3000"[Date - Publication]) | [2010-2023]/py |

# Supplementary Table 2. Calendar years of “Universal Test and Treat” (UTT) Implementation by country.

*Note that WHO calendar years were used for countries whose UTT rollout could not be ascertained*.

| Country | Option B+ (Treat all pregnant and breastfeeding women) | Test and Treat < 1 | Test and Treat < 5 | Test and Treat < 10 | Test and Treat < 15 | Test and Treat All |
| --- | --- | --- | --- | --- | --- | --- |
| WHO | 2013^1^ | 2008 (<1), 2010 (<2)^1^ | 2013^1^ | 2015^2^ | 2015^2^ | 2015^2^ |
| Angola | 2013^1^ | 2011^3^ | 2014^4^ |  |  |  |
| Botswana | 2016^4^ | 2012^3^ |  |  | 2016^4^ | 2016^5^ |
| Comoros |  |  |  |  |  |  |
| Eritrea |  |  |  |  |  |  |
| Eswatini (Swaziland) | 2015^4^ |  |  | 2015^3^ |  | 2016^4^ |
| Ethiopia | 2013^1^ | 2012^3^ |  |  | 2014^4^ | 2016^4^ |
| Kenya | 2013^6^ |  |  | 2013^3^ | 2016^4^ | 2016^7^ |
| Lesotho | 2013^1^ |  | 2014^3^ |  | 2016^4^ | 2016^5^ |
| Madagascar | 2013^4^ | 2013^4^ |  |  |  |  |
| Malawi | 2011^8^ |  | 2014^3^ |  | 2016^4^ | 2016^4^ |
| Mauritius |  |  |  |  |  |  |
| Mozambique | 2016^4^ | 2013^4^ | 2014^3^ |  | 2016^4^ | 2016^4^ |
| Namibia | 2016^4^ |  |  |  | 2013^4^ | 2016^4^ |
| Rwanda | 2012^6^ |  | 2010^3^ |  | 2016^4^ | 2016^4^ |
| Seychelles |  |  |  |  |  |  |
| South Africa | 2016^4^ |  | 2013^3^ |  | 2016^4^ | 2016^4^ |
| South Sudan | 2014^4^ |  | 2014^4^ |  |  |  |
| Uganda | 2012^6^ |  |  |  | 2012^3^ | 2016^4^ |
| Tanzania | 2013^9^ | 2012 (<2)^3^ |  |  | 2014^3^ | 2015^4^ |
| Zambia | 2013^6^ | 2010 (<2)^3^ |  |  | 2012^3^ | 2016^4^ |
| Zimbabwe | 2016^4^ |  | 2013^3^ |  | 2016^4^ | 2016^10^ |

When “Test and Treat” was implemented earlier for younger ages, then “Test or Treat All” means that this was the year when “Test and Treat” was implemented for older ages. Using WHO as an example, the table reads as: 2008 was when “Test and Treat” was introduced to infants aged <1, 2010 for children aged 1-2, 2013 for children aged 3-5, and 2015 for children and adolescents older than age 5.

Studies were selected on whether the calendar era of the study implementation occurred during or overlapped with the country’s UTT implementation, and the study population age ranges contained or overlapped with children and adolescents aged 19 and younger with a maximum overlap of 29 years old. Relevant statistics extracted for the systematic review and meta analysis do not include statistics outside of the UTT implementation years and age ranges of interest. For example, consider a study that is a nationally representative survey of all ages of individuals living with -HIV, with viral suppression proportions reported among 0-4, 5-14, 15-29, 30-59, 60+, and during the calendar eras 2007-2010, 2010-2013, and 2014-2016. Following the WHO years of UTT implementation, we would extract age-specific statistics for the following: for 2007-2010, 0-4 years; for 2010-2013, 0-4 years; for 2014-2016; 5-14 years and 15-29 years.

Sources:

1. World Health Organization. Global Update on HIV Treatment 2013: Results, Impact and Opportunities. June 2013 <https://www.unaids.org/sites/default/files/media_asset/20130630_treatment_report_en_0.pdf>
2. World Health Organization. Consolidated guidelines on the use of antiretroviral drugs for treating and preventing HIV infection: recommendations for a public health approach, 2nd ed. June 2016 <https://www.who.int/publications/i/item/9789241549684>
3. International Association of Providers of AIDS Care. Review of Global HIV Treatment Guidelines from 149 Countries. July 2015 <https://web.archive.org/web/20160328043154if_/http://www.hivpolicywatch.org:80/duremaps/data/doc/IAPAC-ART-guidelines-review-presentation.ppt>
4. International Association of Providers of AIDS Care. Global HIV Policy Watch. July 2017 <https://hivpolicywatch.org/index.html>
5. Gupta S and Granich R. When will sub-Saharan Africa adopt HIV treatment for all? South Afr J HIV Med 2016; 17(1): 459.
6. Giphart A. Rollout of Option B+: A Multi-Country Experience. Elizabeth Glaser Pediatric AIDS Foundation (EGPAF). June 2013. <https://www.hsph.harvard.edu/wp-content/uploads/sites/2413/2014/05/Anja-Giphart.pdf>
7. Kimanga DO et al. Uptake and effect of universal test-and-treat on twelve months retention and initial virologic suppression in routine HIV program in Kenya. PLOS One 17(11):e0277675. November 22, 2022.
8. Chersich MF, Newbatt E, Ng’oma K, de Zoysa I. UNICEF’s contribution to the adoption and implementation of option B+ for preventing mother-to-child transmission of HIV: a policy analysis. Globalization and Health 14(55). 2018.
9. Kalua T et al. Lessons Learned From Option B+ in the Evolution Toward “Test and Start” From Malawi, Cameroon, and the United Republic of Tanzania. J Acquir Immune Defic Syndr 75(Suppl1): S43-S50. May 2017.
10. Rufu A et al. Implementation of the ‘Test and Treat’ policy for newly diagnosed people living with HIV in Zimbabwe in 2017. Public Health Action 8(3): 145-150. Sep 2018.

# Supplementary Table 3. Characteristics of studies included in the systematic review.

| **Author, Year** | **Care Continuum Indicators** | **Age range in the entire study** | **Country (ies)** | **Health Facility Setting** | **Urban/ Rural** | **Study Design** | **Follow-up Months** | **Study Population** | **Relevant Sample Size** | **Relevant calendar years of study** | **Risk of bias assessment score*** |
| --- | --- | --- | --- | --- | --- | --- | --- | --- | --- | --- | --- |
| Adedimeji 2017 | Linkage | Children under 15 | Rwanda | Health center/ Clinic | Both urban and rural | Cohort | 120 | Living with HIV | 1397 | 2004-2013 | 8/9 |
| Ahonkhai 2021 | Retention | Adults 15 and older | Mozambique | Health center/ Clinic | Rural | Cohort | 60 | On ART | 24840 | 2012-2016 | 8/9 |
| Alemayehu 2020 | Adherence, Retention | Children and adolescents 19 and younger | Ethiopia | Hospital | Both urban and rural | Cohort | 156 | On ART (On Second-Line ART) | 76 | 2006-2018 | 8/9 |
| Alhaj 2019 | Retention | Children and adults 10 and older | Malawi | Health center/ Clinic, Hospital | Both urban and rural | Cohort | 12 | On ART | 124 | 2015-2017 | 8/9 |
| Alibi 2023 | Suppressed | Adolescents 10-19 | Malawi | Health center/ Clinic | Both urban and rural | Cohort | 24 | On ART | 233 | 2018-2022 | 7/9 |
| Ally 2023 | Adherence, Suppressed | Children under 15 | Tanzania | Home/ Community | Both urban and rural | Cross-sectional | NA | On ART (Orphans/ Vulnerable children) | 1980 | 2018-2020 | 8/9 |
| Amour 2022 | Suppressed | Children and adults 10 and older | Tanzania | Health center/ Clinic | Urban | Cohort | 60 | On ART | 1697 | 2015-2019 | 8/9 |
| Amour 2022 | Adherence | Children and adults 10 and older | Tanzania | Health center/ Clinic | Urban | Cohort | 60 | On ART | 4961 | 2015-2019 | 7/8 |
| Amzel 2018 | Retention, Suppressed | All ages | Lesotho | Health center/ Clinic, Hospital | Both urban and rural | Cohort | 12 | On ART | 1535 | 2015-2017 | 8/9 |
| Antelman 2022 | Adherence, Retention | Adolescents 10-19 | Tanzania | Health center/ Clinic | Both urban and rural | Cohort | 48 | On ART | 645 | 2015-2019 | 8/9 |
| Arpadi 2019 | Linkage, Retention, Suppressed | Children under 5 | Rwanda | Health center/ Clinic, Hospital | Both urban and rural | Cohort | 71 | Living with HIV | 374 | 2009-2015 | 9/9 |
| Augustine 2021 | Linkage | Infants | Zimbabwe | Health center/ Clinic, Hospital | Both urban and rural | Cohort | 9 | Living with HIV | 40 | 2017 | 8/9 |
| Ayieko 2019 | Linkage | Adults 15 and older | Uganda, Kenya | Home/ Community | Both urban and rural | Experimental | 12 | Living with HIV | 2051 | 2013-2014 | 8/9 |
| Bacha 2022 | Linkage, Suppressed | Children and adolescents 19 and younger | Botswana, Eswatini, Lesotho, Malawi, Tanzania, Uganda | Health center/ Clinic | Both urban and rural | Cohort | 72 | Living with HIV | 25370 | 2014-2019 | 8/9 |
| Bacha 2023 | Suppressed | Children and adolescents 19 and younger | Botswana, Eswatini, Lesotho, Malawi, Tanzania, Uganda | Health center/ Clinic | Both urban and rural | Cohort | 36 | On ART | 9419 | 2017-2020 | 8/9 |
| Bachanas 2021 | Linkage, Retention, Suppressed | Adults 15 and older | Botswana | Health center/ Clinic | Both urban and rural | Cohort | 66 | Living with HIV | 542 | 2013-2018 | 8/9 |
| Baisley 2019 | Linkage | Adults 15 and older | South Africa | Home/ Community | Rural | Cohort | 12 | Living with HIV | 209 | 2017 | 7/9 |
| Bajaria 2021 | Linkage | Children and adolescents 19 and younger | Tanzania | Home/ Community | Both urban and rural | Cohort | 12 | Living with HIV (Orphans/Vulnerable children) | 14538 | 2017-2018 | 8/9 |
| Barnhart 2022 | Retention, Adherence, Suppressed | Adults 15 and older | Rwanda | Health center/ Clinic | Rural | Cohort | 12 | On ART (Pregnant women) | 469 | 2017-2019 | 8/9 |
| Bayleyegn 2021 | Adherence, Suppressed | Children under 15 | Ethiopia | Hospital | Both urban and rural | Cross-sectional | NA | On ART | 253 | 2020-2021 | 7/8 |
| Bekele 2022 | Linkage | Children under 15 | Ethiopia | Health center/ Clinic | Both urban and rural | Cohort | 14 | Living with HIV | 41 | 2017-2018 | 8/9 |
| Berihun 2023 | Adherence, Suppressed | Children under 15 | Ethiopia | Health center/ Clinic | Both urban and rural | Cross-sectional | NA | On ART | 522 | 2022 | 8/9 |
| Bermudez 2018 | Suppressed | Adolescents 10-19 | Uganda | Health center/ Clinic | Both urban and rural | Cohort | 24 | On ART | 702 | 2012-2017 | 7/8 |
| Bianchi 2019 | Linkage | Infants | Kenya, Lesotho, Mozambique, Rwanda, Eswatini, Zimbabwe | Health center/ Clinic | Both urban and rural | Cohort | NA | Living with HIV | 789 | 2014-2017 | 8/9 |
| Bimer 2021 | Retention | Children under 15 | Ethiopia | Hospital | Both urban and rural | Cohort | 84 | On ART | 254 | 2013-2019 | 8/9 |
| Biru 2018 | Retention | Children under 15 | Ethiopia | Health center/ Clinic | Both urban and rural | Cohort | 12 | On ART | 304 | 2014-2016 | 8/9 |
| Bitwale 2021 | Adherence, Suppressed | Children and adolescents 19 and younger | Tanzania | Hospital, Health center/ Clinic | Both urban and rural | Cross-sectional | NA | On ART | 300 | 2018-2019 | 7/8 |
| Biyazin 2022 | Adherence, Retention | Children under 15 | Ethiopia | Hospital | Both urban and rural | Cohort | 60 | On ART | 251 | 2014-2018 | 8/9 |
| Boeke 2021 | Linkage | Infants | Ethiopia, Kenya, Zimbabwe | Health center/ Clinic, Hospital | Both urban and rural | Cohort | 12 | Living with HIV | 127 | 2017-2019 | 8/9 |
| Bolton-Moore 2022 | Linkage, Retention | Children and adolescents 19 and younger | Zambia | Health center/ Clinic, Hospital | Both urban and rural | Cohort | 108 | Living with HIV | 26214 | 2011-2019 | 7/9 |
| Bossard 2022 | Diagnosis, Linkage, Suppressed | Children and adults 10 and older | Malawi | Home/ Community | Rural | Cross-sectional | NA | Living with HIV (Female sex workers) | 12 | 2019 | 7/7 |
| Brathwaite 2021 | Adherence, Suppressed | Adolescents 10-19 | Uganda | Health center/ Clinic | Rural | Cohort | 36 | On ART | 637 | 2012-2018 | 8/9 |
| Brown 2016 | Retention | Adults 15 and older | Kenya, Uganda | Home/ Community | Rural | Experimental | 12 | Living with HIV | 257 | 2013-2015 | 8/9 |
| Brown 2018 | Diagnosis, Linkage, Suppressed | Adults 15 and older | Zimbabwe, Malawi, Zambia, Uganda, Eswatini, Tanzania, Lesotho | Home/ Community | Both urban and rural | Cross-sectional | NA | Living with HIV | 29949 | 2015-2017 | 8/9 |
| Burgos-Soto 2020 | Diagnosis, Linkage, Suppressed | Adults 15 and older | Uganda | Home/ Community | Rural | Cross-sectional | NA | Living with HIV (Fishing communities) | 33 | 2016 | 9/9 |
| Cassidy 2022 | Retention | Children and adults 10 and older | South Africa | Health center/ Clinic | Urban | Cohort | 120 | On ART | 5738 | 2008-2018 | 8/9 |
| Chanie 2022 | Retention | Children under 15 | Ethiopia | Hospital | Both urban and rural | Cohort | 168 | On ART | 357 | 2005-2018 | 8/9 |
| Charles 2022 | Retention | All ages | Tanzania | Home/ Community | Both urban and rural | Cohort | 48 | On ART (Orphans/Vulnerable children) | 5304 | 2016-2021 | 8/9 |
| Chouraya 2019 | Suppressed | Children under 15 | Eswatini | Health center/ Clinic | Both urban and rural | Cross-sectional | NA | On ART | 377 | 2017-2018 | 8/9 |
| Ciccacci 2020 | Retention | Adolescents 10-19 | Malawi | Health center/ Clinic | Both urban and rural | Cohort | 12 | On ART | 425 | 2020-2022 | 7/9 |
| Ciccacci 2023 | Retention, Suppressed | Adolescents 10-19 | Mozambique | Health center/ Clinic | Both urban and rural | Cohort | 3 | On ART | 2575 | 2017 | 8/9 |
| Cluver 2021 | Adherence | Adolescents 10-19 | South Africa | Health center/ Clinic | Both urban and rural | Cohort | 18 | On ART | 969 | 2015-2018 | 8/9 |
| Cluver 2023 | Adherence | Adolescents 10-19 | South Africa | Health center/ Clinic | Both urban and rural | Cohort | 36 | On ART | 980 | 2014-2016 | 8/9 |
| Conan 2021 | Diagnosis, Linkage, Suppressed | Adults 15 and older | Malawi | Home/ Community | Both urban and rural | Cross-sectional | NA | Living with HIV | 107 | 2012-2018 | 8/9 |
| Conan 2021 | Diagnosis, Linkage, Suppressed | Adults 15 and older | Kenya | Home/ Community | Both urban and rural | Cross-sectional | NA | Living with HIV | 3360 | 2016-2017 | 9/9 |
| Conan 2022 | Diagnosis, Linkage, Suppressed | Adults 15 and older | South Africa | Home/ Community | Both urban and rural | Cross-sectional | NA | Living with HIV | 205 | 2013-2018 | 8/9 |
| Crowley 2020 | Adherence, Suppressed | Adolescents 10-19 | South Africa | Health center/ Clinic, Hospital | Both urban and rural | Cross-sectional | NA | On ART | 385 | 2017 | 5/8 |
| Denoeud-Ndam 2023 | Linkage | Children under 15 | Zimbabwe | Hospital | Both urban and rural | Cohort | 1 | Living with HIV | 59 | 2020-2021 | 8/9 |
| Desta 2020 | Adherence | Adults 15 and older | Ethiopia | Health center/ Clinic, Hospital | Both urban and rural | Cross-sectional | NA | On ART | 420 | 2015-2019 | 8/9 |
| Desta 2020 | Suppressed | Adults 15 and older | Ethiopia | Health center/ Clinic, Hospital | Both urban and rural | Cohort | 48 | On ART | 420 | 2015-2019 | 8/9 |
| deWaal 2023 | Suppressed | Infants | South Africa | Health center/ Clinic, Hospital | Both urban and rural | Cohort | 12 | On ART | 467 | 2006-2019 | 8/9 |
| Dorward 2020 | Retention, Suppressed | Adults 15 and older | South Africa | Health center/ Clinic | Both urban and rural | Cohort | 18 | Living with HIV | 807 | 2016-2019 | 8/9 |
| Dougherty 2019 | Linkage | Children under 15 | Tanzania | Health center/ Clinic, Hospital | Both urban and rural | Cohort | 18 | Living with HIV | 47 | 2015 | 8/9 |
| Dougherty 2021 | Linkage | Infants | Zambia | Health center/ Clinic, Hospital | Both urban and rural | Cohort | 12 | Living with HIV | 214 | 2016-2018 | 8/9 |
| Dunlop 2022 | Retention | Children under 15 | South Africa | Health center/ Clinic, Hospital | Urban | Cohort | 168 | On ART | 7630 | 2004-2018 | 8/9 |
| Dzangare 2016 | Retention | Adults 15 and older | Zimbabwe | Health center/ Clinic, Hospital | Rural | Cohort | 6 | Living with HIV (Pregnant women) | 16 | 2014 | 8/9 |
| Edun 2022 | Adherence, Suppressed | Adolescents 10-19 | South Africa | Health center/ Clinic | Both urban and rural | Cohort | 48 | On ART | 813 | 2014-2018 | 7/8 |
| Elashi 2022 | Suppressed | Adolescents 10-19 | South Africa | Home/ Community | Both urban and rural | Cohort | 12 | On ART | 4475 | 2019 | 8/9 |
| Endalamaw Alamneh 2023 | Suppressed | Children and adults 10 and older | Ethiopia | Health center/ Clinic | Both urban and rural | Cross-sectional | NA | Living with HIV (Pregnant women) | 79 | 2021-2022 | 5/7 |
| Etoori 2018 | Linkage | Adults 15 and older | Eswatini | Health center/ Clinic | Rural | Cohort | 6 | Living with HIV (Pregnant women) | 271 | 2013-2014 | 8/9 |
| Fenta 2021 | Suppressed | Children under 15 | Ethiopia | Hospital | Both urban and rural | Cross-sectional | NA | On ART | 273 | 2019 | 8/9 |
| Filiatreau 2021 | Suppressed | Children and adults 10 and older | South Africa | Home/ Community | Rural | Cross-sectional | NA | Living with HIV | 362 | 2019 | 8/9 |
| Finocchario-Kessler 2021 | Linkage | Infants | Kenya | Hospital | Both urban and rural | Cohort | 3 | Living with HIV | 4 | 2017-2018 | 8/9 |
| Floyd 2020 | Diagnosis, Linkage | Adults 15 and older | Zambia, South Africa | Home/ Community | Both urban and rural | Cohort | 48 | Living with HIV | 389 | 2015-2017 | 8/9 |
| Gelaw 2021 | Adherence, Suppressed | Children under 15 | Ethiopia | Health center/ Clinic | Both urban and rural | Cross-sectional | NA | On ART | 399 | 2020 | 8/9 |
| Gemechu 2022 | Adherence | Children under 15 | Ethiopia | Hospital | Both urban and rural | Cohort | 132 | Living with HIV | 284 | 2009-2019 | 8/9 |
| Gibbs. 2022 | Diagnosis, Linkage, Suppressed | Children and adults 10 and older | South Africa | Home/ Community | Both urban and rural | Cohort | 18 | Living with HIV | 1118 | 2017-2018 | 7/8 |
| Gill 2020 | Linkage | Children under 15 | Kenya, Uganda | Health center/ Clinic | Both urban and rural | Cohort | 2 | Living with HIV | 172 | 2017-2018 | 7/8 |
| Gitahi-Kamau 2020 | Adherence, Suppressed | Adolescents 10-19 | Kenya | Health center/ Clinic | Both urban and rural | Cross-sectional | NA | Living with HIV | 82 | 2018-2019 | 5/8 |
| Gordon 2022 | Suppressed | Adolescents 10-19 | Uganda | Health center/ Clinic | Both urban and rural | Cross-sectional | NA | On ART | 249 | 2019 | 7/8 |
| Graca 2022 | Linkage | Infants | Mozambique | Hospital | Both urban and rural | Cohort | 6 | Living with HIV | 31 | 2020 | 8/9 |
| Grasso 2021 | Linkage | All ages | Namibia | Home/ Community | Both urban and rural | Cross-sectional | NA | Living with HIV | 109 | 2017 | 8/9 |
| Gross 2023 | Linkage | Children under 15 | Mozambique, South Africa, Kenya, Uganda, Tanzania, Zimbabwe, Ethiopia, Malawi, Zambia, South Sudan | Health center/ Clinic | Both urban and rural | Cross-sectional | NA | Living with HIV | 86101 | 2018-2019 | 8/9 |
| Haghighat 2019 | Retention, Suppressed | Adolescents 10-19 | South Africa | Health center/ Clinic | Both urban and rural | Cohort | 48 | On ART | 951 | 2014-2017 | 8/9 |
| Hakim 2022 | Diagnosis, Linkage, Suppressed | Adults 15 and older | South Sudan | Home/ Community | Both urban and rural | Cohort | 6 | Living with HIV (Female sex workers) | 46 | 2015-2017 | 8/8 |
| Hansoti 2019 | Suppressed | Children and adolescents 19 and younger | South Africa | Hospital | Both urban and rural | Cross-sectional | NA | Living with HIV | 33 | 2016 | 8/9 |
| Hibstie 2020 | Retention, Adherence | Children under 15 | Ethiopia | Hospital | Both urban and rural | Cohort | 156 | On ART | 408 | 2005-2019 | 8/9 |
| Humphrey 2019 | Suppressed | Children under 15 | Kenya | Health center/ Clinic | Both urban and rural | Cross-sectional | NA | On ART | 1698 | 2015-2017 | 8/9 |
| Iyun 2021 | Retention | Children under 5 | Kenya, Lesotho, Malawi, Mozambique, Rwanda, South Africa, Uganda, Zambia, Zimbabwe | Health center/ Clinic, Hospital | Both urban and rural | Cohort | 156 | On ART | 3221 | 2006-2017 | 8/9 |
| Iyun 2929 | Retention | Infants | South Africa | Health center/ Clinic, Hospital | Both urban and rural | Cohort | 144 | On ART | 1847 | 2006-2017 | 8/9 |
| Jackson 2022 | Adherence, Suppressed | Children and adolescents 19 and younger | Zimbabwe, Malawi | Health center/ Clinic | Both urban and rural | Experimental | 11 | Living with HIV (With chronic lung disease) | 345 | 2016-2019 | 8/9 |
| Jonnalagadda 2021 | Diagnosis, Linkage, Suppressed | Children under 15 | Malawi | Home/ Community | Both urban and rural | Cross-sectional | NA | Living with HIV | 99 | 2015-2016 | 9/9 |
| Jubilee 2019 | Linkage | All ages | Lesotho | Health center/ Clinic | Both urban and rural | Cohort | 30 | Living with HIV | 140 | 2015-2017 | 8/9 |
| Kabogo 2018 | Adherence, Suppressed | Children and adolescents 19 and younger | Kenya | Home/ Community | Both urban and rural | Cohort | 6 | Living with HIV | 197 | 2016-2017 | 8/9 |
| Kairania 2022 | Adherence | Adolescents 10-19 | Uganda | Health center/ Clinic, Hospital | Both urban and rural | Cross-sectional | NA | On ART | 514 | 2020 | 7/8 |
| Kalawan 2020 | Linkage, Retention, Suppressed | Infants | South Africa | Health center/ Clinic, Hospital | Both urban and rural | Cohort | 12 | Living with HIV | 236 | 2013-2017 | 8/9 |
| Kerschberger 2020 | Retention | Adults 15 and older | Eswatini | Health center/ Clinic, Hospital | Rural | Cohort | 36 | Living with HIV | 416 | 2014-2016 | 8/9 |
| Kim 2015 | Linkage | Infants | Malawi | Health center/ Clinic | Urban | Cohort | 24 | Living with HIV | 22 | 2011-2013 | 8/9 |
| Kose 2022 | Retention, Suppressed | Adolescents 10-19 | Kenya | Health center/ Clinic, Hospital | Both urban and rural | Cohort | 18 | On ART | 325 | 2017-2018 | 8/9 |
| Lain 2022 | Suppressed | Infants | Mozambique | Health center/ Clinic | Both urban and rural | Cohort | 24 | On ART | 39 | 2017-2019 | 5/6 |
| Leshargie 2022 | Adherence, Suppressed | Adolescents 10-19 | Ethiopia | Hospital | Urban | Cohort | 174 | Living with HIV | 928 | 2005-2020 | 8/9 |
| Levy 2021 | Suppressed | Adolescents 10-19 | Kenya | Health center/ Clinic | Both urban and rural | Quasi-experimental | 1 | On ART (Pregnant women) | 181 | 2018-2019 | 8/9 |
| Low 2021 | Diagnosis, Linkage, Suppressed | Adolescents 10-19 | Malawi, Eswatini, Zimbabwe, Zambia, Lesotho | Home/ Community | Both urban and rural | Cross-sectional | NA | Living with HIV | 707 | 2015-2017 | 9/9 |
| Lulseged 2021 | Diagnosis | Adults 15 and older | Ethiopia | Home/ Community | Both urban and rural | Cross-sectional | NA | Living with HIV | 62 | 2017-2018 | 9/9 |
| MacKellar 2018 | Linkage | Adults 15 and older | Eswatini | Health center/ Clinic | Both urban and rural | Cohort | 21 | Living with HIV | 91 | 2015-2017 | 8/9 |
| Madiba 2021 | Adherence | Adolescents 10-19 | Lesotho | Health center/ Clinic | Both urban and rural | Cross-sectional | NA | On ART | 130 | 2020 | 5/8 |
| Maena 2021 | Suppressed | Adolescents 10-19 | Uganda | Health center/ Clinic, Hospital | Rural | Cross-sectional | NA | On ART | 567 | 2020 | 8/9 |
| Mageda 2023 | Adherence, Suppressed | Children under 15 | Tanzania | Health center/ Clinic, Hospital | Rural | Cross-sectional | NA | On ART | 253 | 2021 | 5/7 |
| Mapangisana 2021 | Suppressed | All ages | Zimbabwe | Hospital | Rural | Cohort | 24 | On ART | 306 | 2018 | 8/9 |
| Marinda 2020 | Diagnosis, Linkage, Suppressed | Adults 15 and older | South Africa | Home/ Community | Both urban and rural | Cross-sectional | NA | Living with HIV | 336 | 2020 | 9/9 |
| Martelli 2019 | Adherence, Suppressed | Children and adolescents 19 and younger | Tanzania | Health center/ Clinic | Rural | Cross-sectional | NA | On ART | 72 | 2017 | 8/9 |
| Masaba 2023 | Suppressed | Children under 15 | Kenya | Health center/ Clinic, Hospital | Both urban and rural | Cohort | 6 | Living with HIV | 782 | 2020-2021 | 8/9 |
| Masoza 2022 | Linkage | Infants | Tanzania | Hospital | Both urban and rural | Cross-sectional | NA | Living with HIV | 35 | 2014-2015 | 8/9 |
| Mathamo 2022 | Suppressed | Children and adolescents 19 and younger | South Africa | Health center/ Clinic | Both urban and rural | Cross-sectional | NA | Living with HIV | 102624 | 2018-2022 | 7/9 |
| Matsinhe 2021 | Linkage | Infants | Mozambique | Hospital | Both urban and rural | Cohort | 9 | Living with HIV | 121 | 2017-2018 | 8/9 |
| Mburu 2019 | Suppressed | Adolescents 10-19 | Kenya | Health center/ Clinic | Both urban and rural | Cohort | 27 | On ART | 2195 | 2019 | 7/9 |
| Mburugu 2021 | Suppressed | Children and adults 10 and older | Kenya | Hospital | Both urban and rural | Cohort | 6 | On ART | 159 | 2017 | 8/9 |
| McBride 2019 | Adherence | Adolescents 10-19 | Malawi | Health center/ Clinic | Both urban and rural | Cohort | 6 | On ART | 589 | 2017 | 8/9 |
| Mengistu 2022 | Retention, Adherence | Children under 15 | Eritrea | Hospital | Both urban and rural | Cohort | 300 | Living with HIV | 822 | 2005-2020 | 7/9 |
| Mengistu 2023 | Retention, Adherence, Suppressed | Children and adolescents 19 and younger | Eritrea | Hospital | Both urban and rural | Cohort | 300 | Living with HIV | 822 | 2005-2020 | 8/9 |
| Menshw Snr 2021 | Adherence, Retention | Children under 15 | Ethiopia | Hospital | Both urban and rural | Cohort | 120 | On ART | 488 | 2010-2020 | 7/8 |
| Merrill 2021 | Suppressed | Adults 15 and older | Zambia | Health center/ Clinic | Both urban and rural | Cross-sectional | NA | On ART | 272 | 2017-2019 | 8/9 |
| Mhlanga 2022 | Suppressed | Children and adolescents 19 and younger | Zimbabwe | Hospital | Both urban and rural | Cohort | 60 | Living with HIV | 7592 | 2014-2018 | 8/9 |
| Millar 2020 | Retention, Suppressed | Infants | South Africa | Hospital | Both urban and rural | Cohort | 12 | Living with HIV | 110 | 2015-2019 | 8/9 |
| Moyo 2020 | Suppressed | Children and adolescents 19 and younger | Zimbabwe | Hospital | Both urban and rural | Cohort | 295 | On ART | 295 | 2017-2018 | 8/9 |
| Mugo 2023 | Adherence, Suppressed | Adults 15 and older | Kenya | Health center/ Clinic | Both urban and rural | Cohort | 12 | On ART | 1011 | 2019-2020 | 8/9 |
| Munthali 2020 | Linkage | Children under 15 | Zambia | Health center/ Clinic, Hospital | Both urban and rural | Cohort | 12 | Living with HIV | 7212 | 2004-2017 | 7/9 |
| Munyayi 2020 | Suppressed | Adolescents 10-19 | Namibia | Health center/ Clinic, Hospital | Both urban and rural | Cohort | 24 | On ART | 385 | 2019-2021 | 8/9 |
| Munyayi 2022 | Retention | Adolescents 10-19 | Namibia | Health center/ Clinic, Hospital | Both urban and rural | Cohort | 24 | On ART | 695 | 2019-2021 | 8/9 |
| Munyayi 2023 | Retention, Adherence, Suppressed | Adolescents 10-19 | Namibia | Health center/ Clinic, Hospital | Both urban and rural | Cohort | 24 | On ART | 695 | 2015-2017 | 8/9 |
| Mushy 2023 | Retention | Adults 15 and older | Tanzania | Health center/ Clinic, Hospital | Both urban and rural | Cohort | 24 | On ART | 4195 | 2018-2020 | 8/9 |
| Mussa 2022 | Adherence | Children under 15 | Tanzania | Health center/ Clinic, Hospital | Both urban and rural | Cross-sectional | NA | On ART | 333 | 2018 | 7/8 |
| Mutisya 2022 | Diagnosis, Linkage, Suppressed | Children under 15 | Kenya | Home/ Community | Both urban and rural | Cross-sectional | NA | Living with HIV | 57 | 2018 | 9/9 |
| Muwanguzi 2021 | Retention | Adults 15 and older | Uganda | Health center/ Clinic | Rural | Cross-sectional | NA | On ART | 102 | 2020 | 7/8 |
| Mwangi 2021 | Adherence, Suppressed | Adolescents 10-19 | Kenya | Hospital | Both urban and rural | Cross-sectional | NA | On ART | 908 | 2017 | 7/9 |
| Mwango 2020 | Linkage | Adults 15 and older | Zambia | Health center/ Clinic | Both urban and rural | Cohort | 12 | Living with HIV | 516 | 2018-2019 | 8/9 |
| Nabunya 2023 | Adherence | Adolescents 10-19 | Uganda | Health center/ Clinic | Rural | Experimental | 6 | On ART | 702 | 2012-2018 | 7/8 |
| Nasuuna 2018 | Suppressed | Children and adolescents 19 and younger | Uganda | Health center/ Clinic | Both urban and rural | Cross-sectional | NA | On ART (Previous treatment failure) | 449 | 2015-2016 | 8/9 |
| Natukunda 2019 | Adherence, Suppressed | Adolescents 10-19 | Uganda | Hospital | Both urban and rural | Cross-sectional | NA | On ART | 200 | 2016 | 8/8 |
| Nega 2020 | Suppressed | Children and adults 10 and older | Ethiopia | Hospital | Both urban and rural | Cross-sectional | NA | Living with HIV | 20 | 2019 | 8/9 |
| Negash 2020 | Suppressed | All ages | Ethiopia | Hospital | Both urban and rural | Cross-sectional | NA | On ART | 393 | 2019 | 7/8 |
| Ng'ambi 2022 | Suppressed | All ages | Malawi | National laboratory information system | Both urban and rural | Cross-sectional | NA | On ART | 60737 | 2021 | 9/9 |
| Ngandu 2022 | Suppressed | Adults 15 and older | South Africa | Health center/ Clinic | Rural | Cross-sectional | NA | On ART (Pregnant women) | 105 | 2019 | 6/8 |
| Nguyen 2023 | Adherence | Adolescents 10-19 | Mozambique | Health center/ Clinic | Both urban and rural | Cross-sectional | NA | On ART | 213 | 2019 | 6/8 |
| Nhampossa 2020 | Retention | Children under 15 | Mozambique | Health center/ Clinic, Hospital | Rural | Cohort | 24 | On ART | 351 | 2013-2016 | 8/9 |
| Nimwesiga 2023 | Retention | Adolescents 10-19 | Uganda | Health center/ Clinic, Hospital | Both urban and rural | Cohort | 24 | On ART | 84 | 2019-2021 | 8/9 |
| Njuguna 2020 | Suppressed | Children and adults 10 and older | Kenya | Health center/ Clinic | Both urban and rural | Cross-sectional | NA | Living with HIV | 10096 | 2016-2017 | 8/9 |
| Ntabanganyimana 2022 | Retention | All ages | Rwanda | Health center/ Clinic, Hospital | Both urban and rural | Cohort | 12 | On ART | 60 | 2018-2019 | 8/9 |
| Ntombela 2022 | Diagnosis, Linkage, Suppressed | Children and adults 10 and older | South Africa | Health center/ Clinic | Rural | Cross-sectional | NA | Living with HIV (Pregnant women) | 26 | 2016-2017 | 8/9 |
| Nuttall 2018 | Suppressed | Children and adolescents 19 and younger | South Africa | Hospital | Both urban and rural | Cohort | 36 | On ART (On Third-Line ART) | 35 | 2013-2016 | 8/9 |
| Nyakato 2022 | Retention, Suppressed | Adolescents 10-19 | South Africa | Health center/ Clinic, Hospital | Both urban and rural | Cohort | 24 | On ART | 2733 | 2004-2019 | 8/9 |
| Nzivo 2023 | Suppressed | All ages | Kenya | Hospital | Both urban and rural | Cross-sectional | NA | Living with HIV | 85 | 2015-2021 | 8/9 |
| Okonji 2021 | Suppressed | Adolescents 10-19 | South Africa | Health center/ Clinic | Both urban and rural | Cohort | 216 | On ART | 9386 | 2002-2019 | 8/9 |
| Onyango 2023 | Suppressed | Children and adolescents 19 and younger | Kenya | Health center/ Clinic, Hospital | Both urban and rural | Cross-sectional | NA | On ART (Orphans/Vulnerable children) | 31291 | 2019-2020 | 8/9 |
| Osman 2020 | Adherence, Suppressed | Children under 15 | Ethiopia | Health center/ Clinic | Both urban and rural | Cross-sectional | NA | On ART | 140 | 2019 | 8/9 |
| Pathmanathan 2021 | Linkage | Adults 15 and older | Mozambique | Home/ Community | Both urban and rural | Cross-sectional | NA | Living with HIV | 115 | 2014-2017 | 7/9 |
| Philbert 2023 | Diagnosis, Linkage, Suppressed | Children and adults 10 and older | Tanzania | Health center/ Clinic | Both urban and rural | Cross-sectional | NA | Living with HIV | 1124 | 2016-2021 | 9/9 |
| Porter 2020 | Suppressed | Children under 15 | South Africa | Health center/ Clinic, Hospital | Rural | Cross-sectional | NA | On ART | 235 | 2017-2019 | 5/7 |
| Ravishankar 2022 | Suppressed | Children and adolescents 19 and younger | Zambia | Hospital | Both urban and rural | Cross-sectional | NA | On ART (Children with Epilepsy) | 36 | 2016-2017 | 8/9 |
| Ross 2019 | Suppressed | Adults 15 and older | Rwanda | Health center/ Clinic, Hospital | Both urban and rural | Cohort | 40 | Living with HIV | 448 | 2016-2019 | 8/9 |
| Ross 2020 | Retention, Suppressed | Adults 15 and older | Rwanda | Health center/ Clinic, Hospital | Both urban and rural | Cross-sectional | NA | Living with HIV | 1028 | 2018 | 8/9 |
| Rufu 2018 | Linkage, Retention | All ages | Zimbabwe | Health center/ Clinic | Both urban and rural | Cohort | 3 | Living with HIV | 245 | 2014-2017 | 7/9 |
| Rugemalila 2023 | Linkage | Children and adults 10 and older | Tanzania | Health center/ Clinic | Both urban and rural | Cross-sectional | NA | On ART | 570 | 2017 | 8/9 |
| Schrubbe 2023 | Suppressed | Adults 15 and older | Ethiopia, Eswatini, Lesotho, Malawi, Namibia, Uganda, Zambia, Zimbabwe | Home/ Community | Both urban and rural | Cross-sectional | NA | Living with HIV (Pregnant women) | 94 | 2020 | 9/9 |
| Shah 2018 | Suppressed | All ages | Kenya | Health center/ Clinic | Urban | Cohort | 34 | Living with HIV (Street-connected children) | 27 | 2015-2017 | 9/9 |
| Sher 2020 | Linkage, Retention, Suppressed | Adolescents 10-19 | South Africa | Health center/ Clinic | Both urban and rural | Cohort | 12 | On ART | 482 | 2015-2017 | 8/9 |
| Shiau 2021 | Suppressed | Infants | South Africa | Hospital | Both urban and rural | Cohort | 18 | Living with HIV | 61 | 2002-2016 | 7/9 |
| Sifr 2018 | Suppressed | Children under 15 | Ethiopia | Hospital | Both urban and rural | Cohort | 60 | On ART | 143 | 2013-2018 | 8/9 |
| Sikhondze 2017 | Retention | Children under 5 | Eswatini | Health center/ Clinic | Both urban and rural | Cohort | 12 | Living with HIV | 32 | 2014-2018 | 8/9 |
| Ssanyu 2020 | Retention | Children under 5 | Uganda | Health center/ Clinic, Hospital | Both urban and rural | Cross-sectional | NA | On ART | 206 | 2014-2016 | 7/9 |
| Sutcliffe 2020 | Adherence | Infants | Zambia | Hospital, Health center/ Clinic | Both urban and rural | Cohort | 36 | Living with HIV | 23 | 2019 | 7/8 |
| Tadesse 2019 | Linkage | Children under 15 | Ethiopia | Health center/ Clinic, Hospital | Both urban and rural | Cohort | 12 | On ART | 110 | 2016-2018 | 8/9 |
| Tadesse 2021 | Suppressed | Children and adolescents 19 and younger | Ethiopia | Health center/ Clinic, Hospital | Both urban and rural | Cohort | 12 | On ART | 484 | 2019 | 8/9 |
| Tanyi 2021 | Adherence, Suppressed | Children and adolescents 19 and younger | Kenya | Hospital | Both urban and rural | Cohort | 12 | On ART | 178 | 2019 | 8/9 |
| Tapera 209 | Suppressed | All ages | Zimbabwe | Home/ Community | Both urban and rural | Cohort | 6 | On ART | 1193 | 2016-2017 | 8/9 |
| Teasdale 2022 | Linkage, Retention, Suppressed | Adults 15 and older | Kenya | Health center/ Clinic, Hospital | Both urban and rural | Cohort | 6 | Living with HIV (Pregnant women) | 543 | 2017-2018 | 7/8 |
| Teasdale 2022 | Diagnosis, Suppressed | Children under 15 | Eswatini, Lesotho, Malawi, Namibia, Tanzania, Zambia, Zimbabwe | Home/ Community | Both urban and rural | Cross-sectional | NA | Living with HIV | 594 | 2015-2017 | 9/9 |
| Technau 2018 | Retention | Infants | South Africa | Hospital | Urban | Cohort | 12 | Living with HIV | 4450 | 2016-2019 | 8/9 |
| Tekliye 2021 | Linkage, Retention, Suppressed | Children and adolescents 19 and younger | Ethiopia | Hospital | Urban | Cohort | NA | On ART (On Second-Line ART) | 75 | 2013-2016 | 8/9 |
| Tesfahunegn 2023 | Adherence, Suppressed | Children under 15 | Ethiopia | Health center/ Clinic, Hospital | Both urban and rural | Cross-sectional | NA | On ART | 250 | 2009-2019 | 8/9 |
| Tesha 2022 | Adherence | Adolescents 10-19 | Tanzania | Health center/ Clinic, Hospital | Both urban and rural | Cohort | 24 | Living with HIV | 25484 | 2016 | 8/9 |
| Thin 2019 | Retention | Adults 15 and older | Lesotho | Home/ Community | Both urban and rural | Cross-sectional | NA | Living with HIV | 330 | 2014-2016 | 9/9 |
| Tong 2020 | Diagnosis, Linkage, Suppressed | Children under 5 | South Sudan | Hospital | Both urban and rural | Cross-sectional | NA | On ART | 126 | 2016-2017 | 9/9 |
| Tsikhutsu 2022 | Adherence | Children and adolescents 19 and younger | Kenya | Health center/ Clinic | Both urban and rural | Cross-sectional | NA | On ART | 935 | 2019 | 7/8 |
| Tsondai 2020 | Suppressed | Adolescents 10-19 | Lesotho, Malawi, Mozambique, South Africa, Zambia, Zimbabwe | Health center/ Clinic, Hospital | Both urban and rural | Cohort | 36 | On ART | 5516 | 2018-2020 | 7/8 |
| Tweya 2020 | Retention, Suppressed | Children and adolescents 19 and younger | Malawi | Health center/ Clinic | Both urban and rural | Cohort | 36 | On ART | 1847 | 2004-2017 | 7/9 |
| Tymejczyk 2020 | Retention, Suppressed | Adolescents 10-19 | Kenya, Malawi, Uganda, Zambia, Rwanda | Health center/ Clinic, Hospital | Both urban and rural | Cohort | 1 | Living with HIV | 2022 | 2014-2017 | 8/9 |
| Umar 2019 | Linkage | Children and adults 10 and older | Malawi | Hospital | Both urban and rural | Cross-sectional | NA | On ART | 209 | 2010-2015 | 8/9 |
| van Liere 2021 | Adherence, Suppressed | Children under 15 | South Africa | Health center/ Clinic, Hospital | Both urban and rural | Cohort | 36 | On ART | 2739 | 2016 | 6/8 |
| Vonasek 2021 | Retention, Suppressed | Children and adolescents 19 and younger | Malawi | Health center/ Clinic | Both urban and rural | Cohort | 8 | On ART | 308 | 2015-2018 | 8/9 |
| Wakokoo 2020 | Suppressed | All ages | Uganda | Health center/ Clinic | Both urban and rural | Cohort | 30 | On ART | 134 | 2016-2018 | 8/9 |
| Weldemariam 2022 | Suppressed | Children under 5 | Ethiopia | Health center/ Clinic,Hospital | Both urban and rural | Cohort | 60 | Living with HIV | 376 | 2016-2018 | 8/9 |
| Wilson 2022 | Adherence | Children and adults 10 and older | Kenya | Health center/ Clinic, Hospital | Both urban and rural | Cohort | 18 | On ART | 5316 | 2014-2019 | 8/9 |
| Woldesenbet 2020 | Suppressed | Adults 15 and older | South Africa | Health center/ Clinic, Hospital | Both urban and rural | Cross-sectional | NA | On ART (Pregnant women) | 504 | 2015-2017 | 8/9 |
| Woldesenbet 2022 | Diagnosis, Linkage, Suppressed | Adults 15 and older | South Africa | Health center/ Clinic, Hospital | Both urban and rural | Cohort | 24 | Living with HIV | 2433 | 2017-2019 | 8/9 |
| Yihun 2019 | Diagnosis, Linkage | Children under 15 | Ethiopia | Hospital | Both urban and rural | Cohort | 72 | On ART | 402 | 2017 | 7/8 |
| Zakaria 2022 | Adherence, Suppressed | Adults 15 and older | Ethiopia | Hospital | Urban | Cohort | 29 | On ART (On Second-Line ART) | 56 | 2011-2012 | 8/9 |
| Zhou 2021 | Suppressed | Adolescents 10-19 | South Africa | Health center/ Clinic, Hospital | Both urban and rural | Cohort | 48 | On ART | 933 | 2018-2021 | 8/9 |
| Zijenah 2022 | Adherence | Infants | Zimbabwe | Health center/ Clinic | Urban | Cohort | 12 | On ART | 7 | 2014-2018 | 7/9 |
| Zingoni 2020 | Suppressed | Adults 15 and older | Zimbabwe | Health center/ Clinic, Hospital | Both urban and rural | Cohort | 156 | On ART | 43972 | 2017-2020 | 7/8 |

* The denominator in the risk of bias assessment score changes because some questions may not be applicable to the study being assessed.


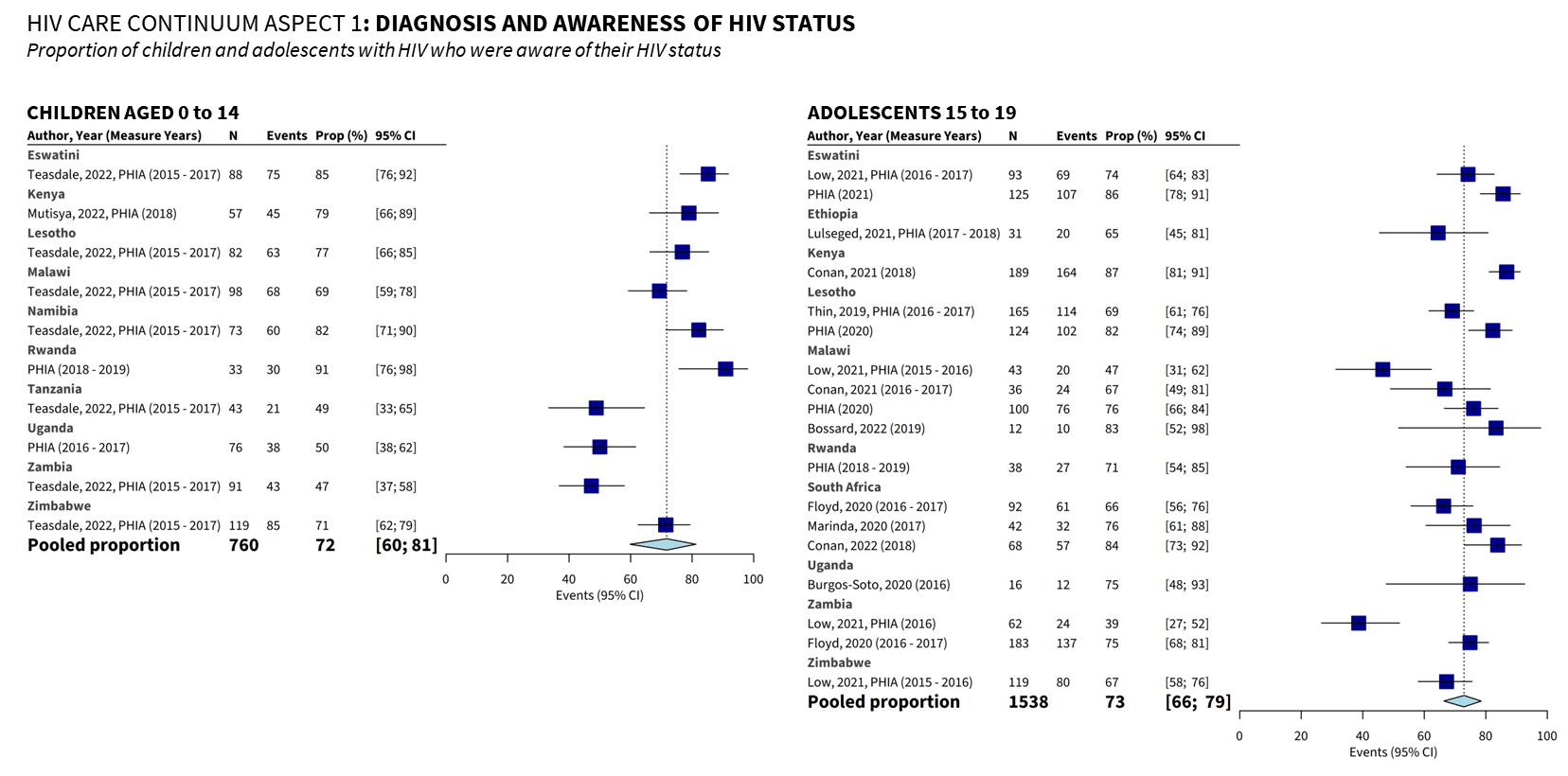


# Supplementary Figure 1. Forest plots of diagnosis and knowledge of HIV status, by children 0-14 and adolescents 15-19, in Eastern and Southern Africa.


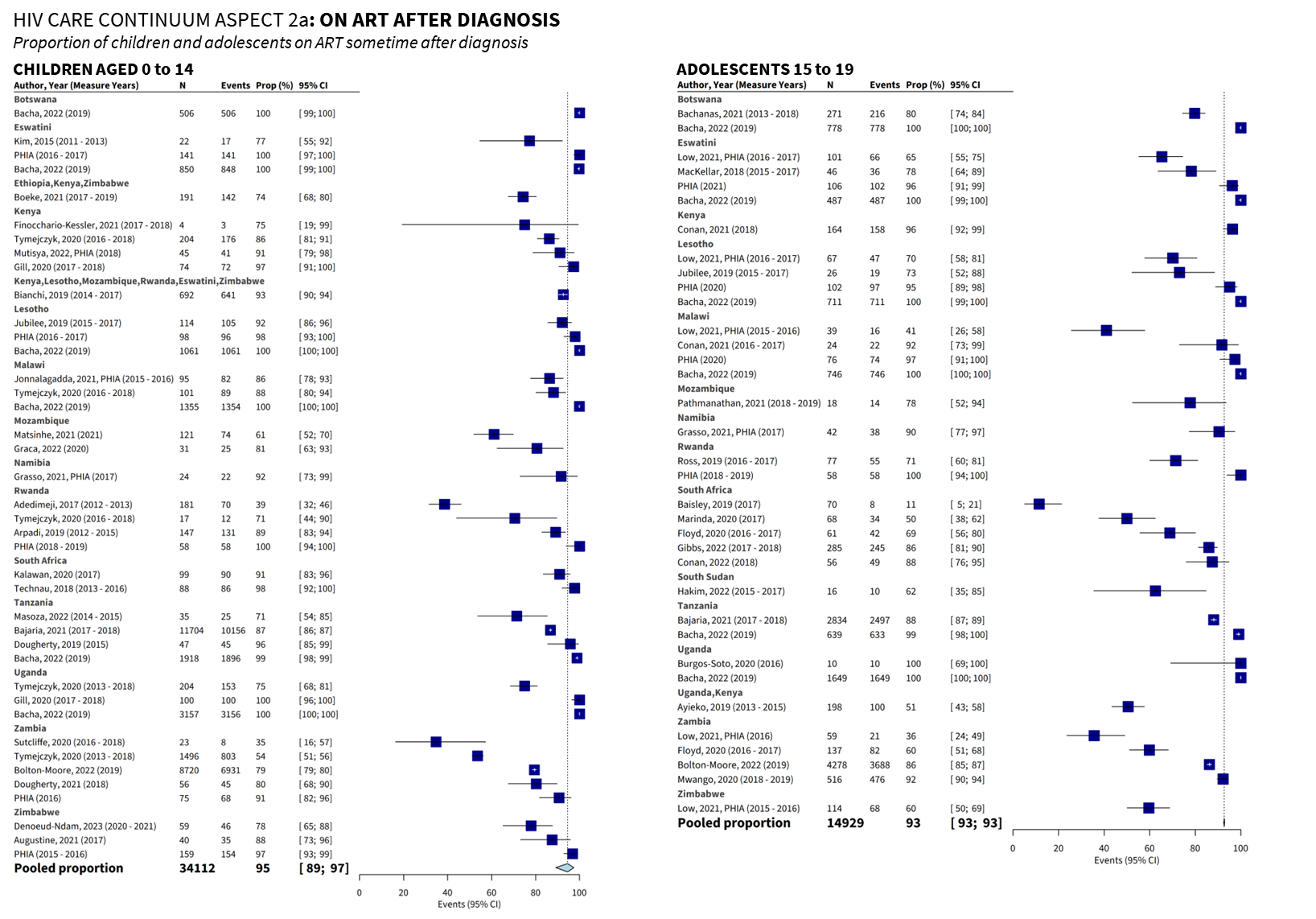


# Supplementary Figure 2. Forest plots of on ART after diagnosis, by children 0-14 and adolescents 15-19, in Eastern and Southern Africa.


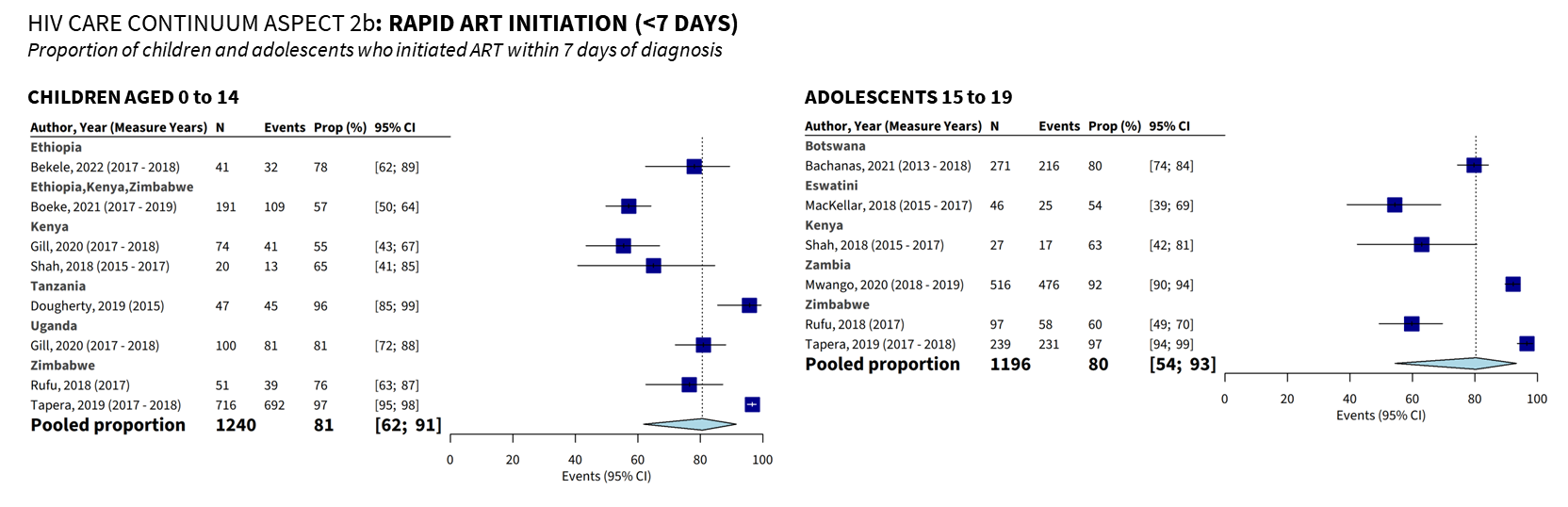


# Supplementary Figure 3. Forest plots of rapid ART initiation (<7 days after diagnosis), by children 0-14 and adolescents 15-19, in Eastern and Southern Africa.


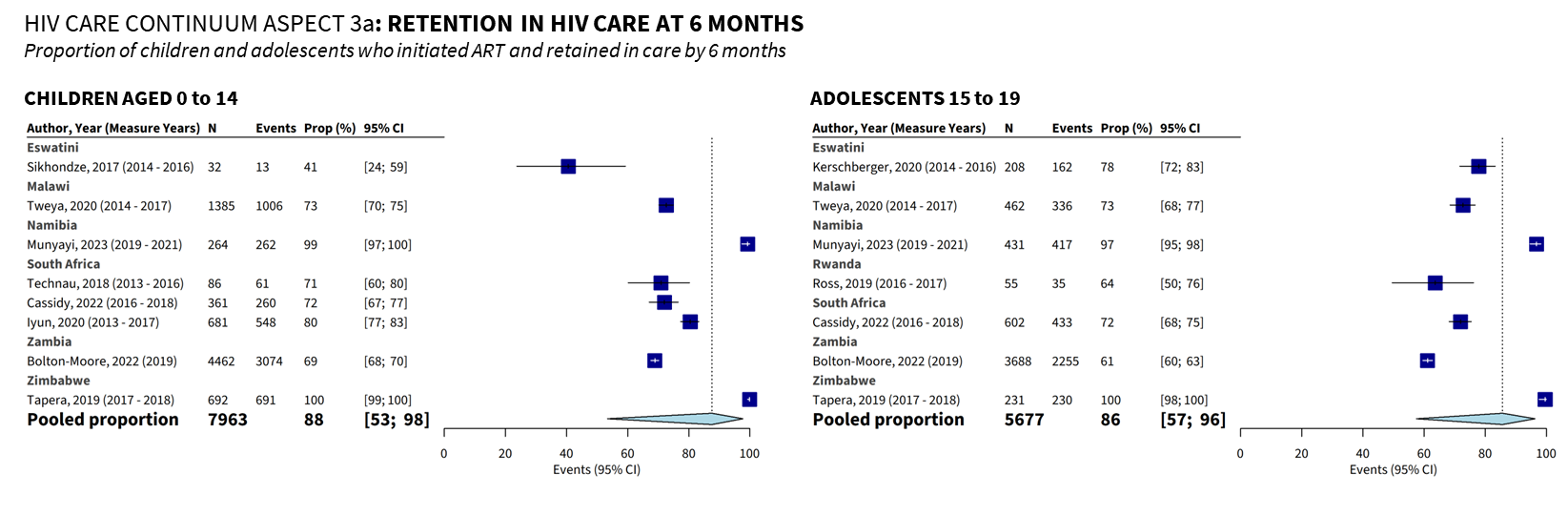


# Supplementary Figure 4. Forest plots of retention in HIV care at 6 months, by children 0-14 and adolescents 15-19, in Eastern and Southern Africa.


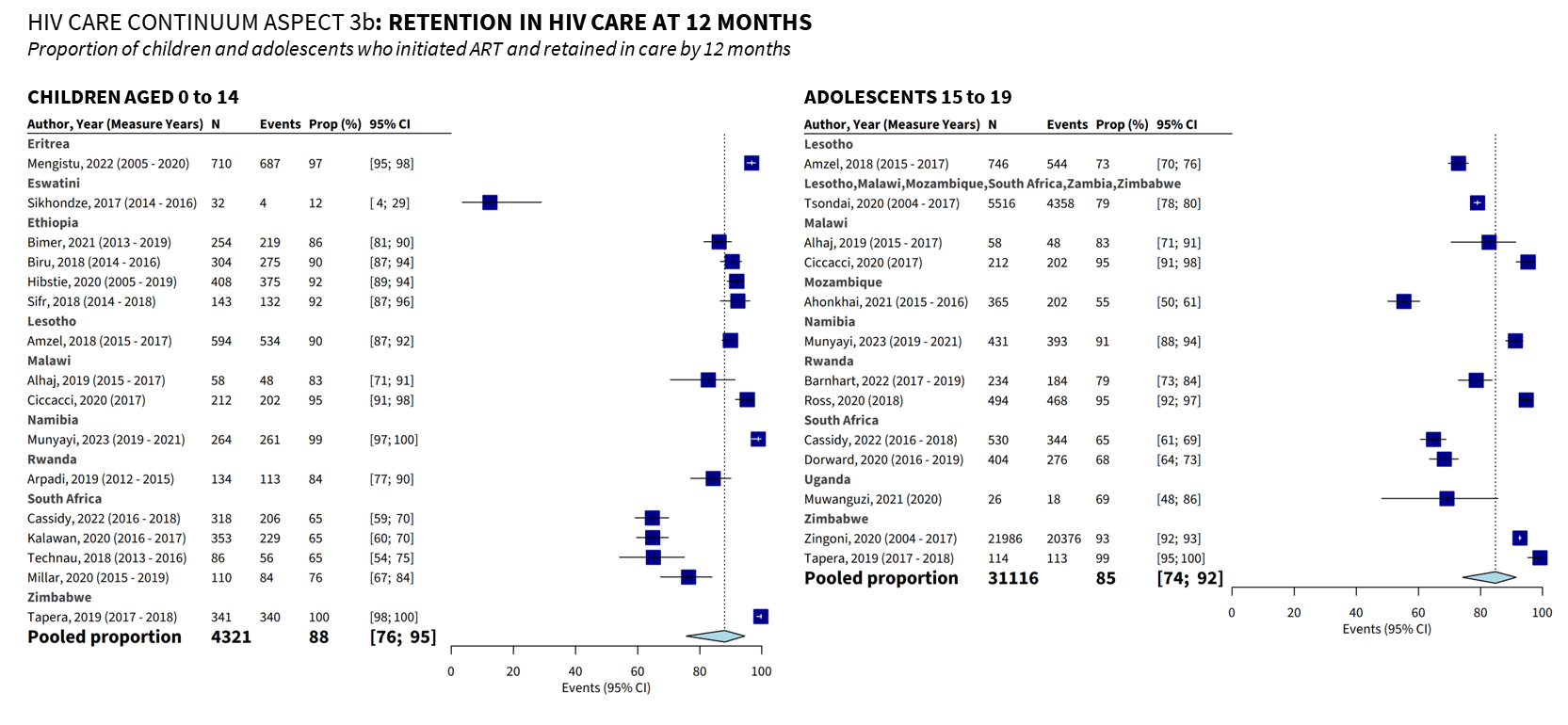


# Supplementary Figure 5. Forest plots of retention in HIV care at 12 months, by children 0-14 and adolescents 15-19, in Eastern and Southern Africa.


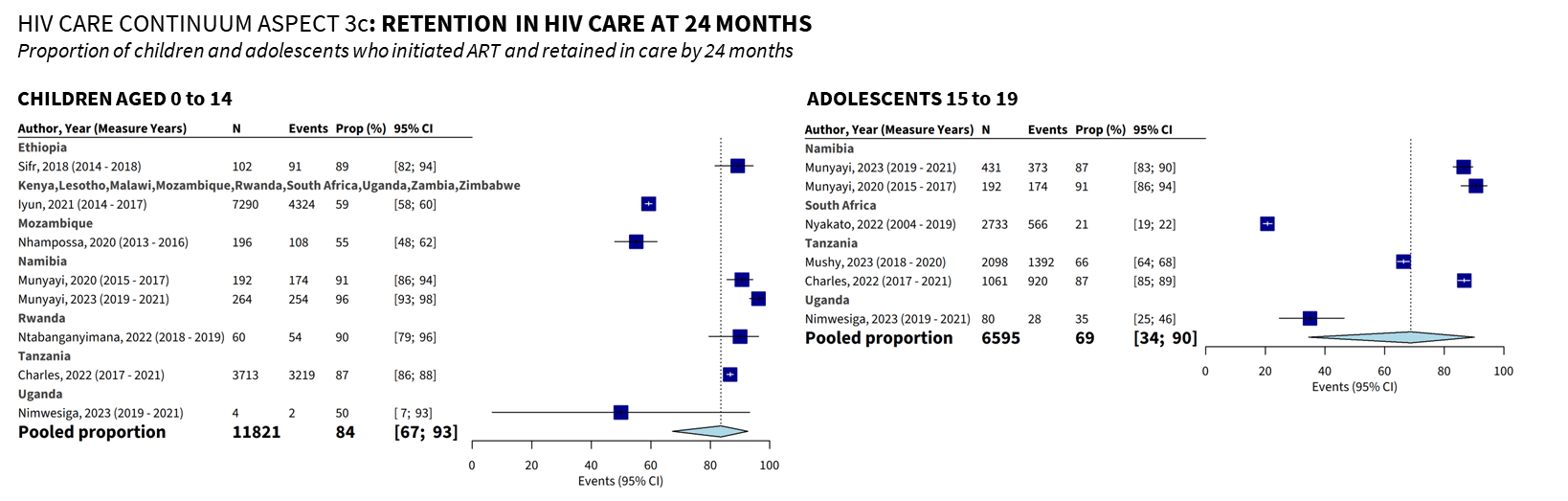


# Supplementary Figure 6. Forest plots of retention in HIV care at 24 months, by children 0-14 and adolescents 15-19, in Eastern and Southern Africa.


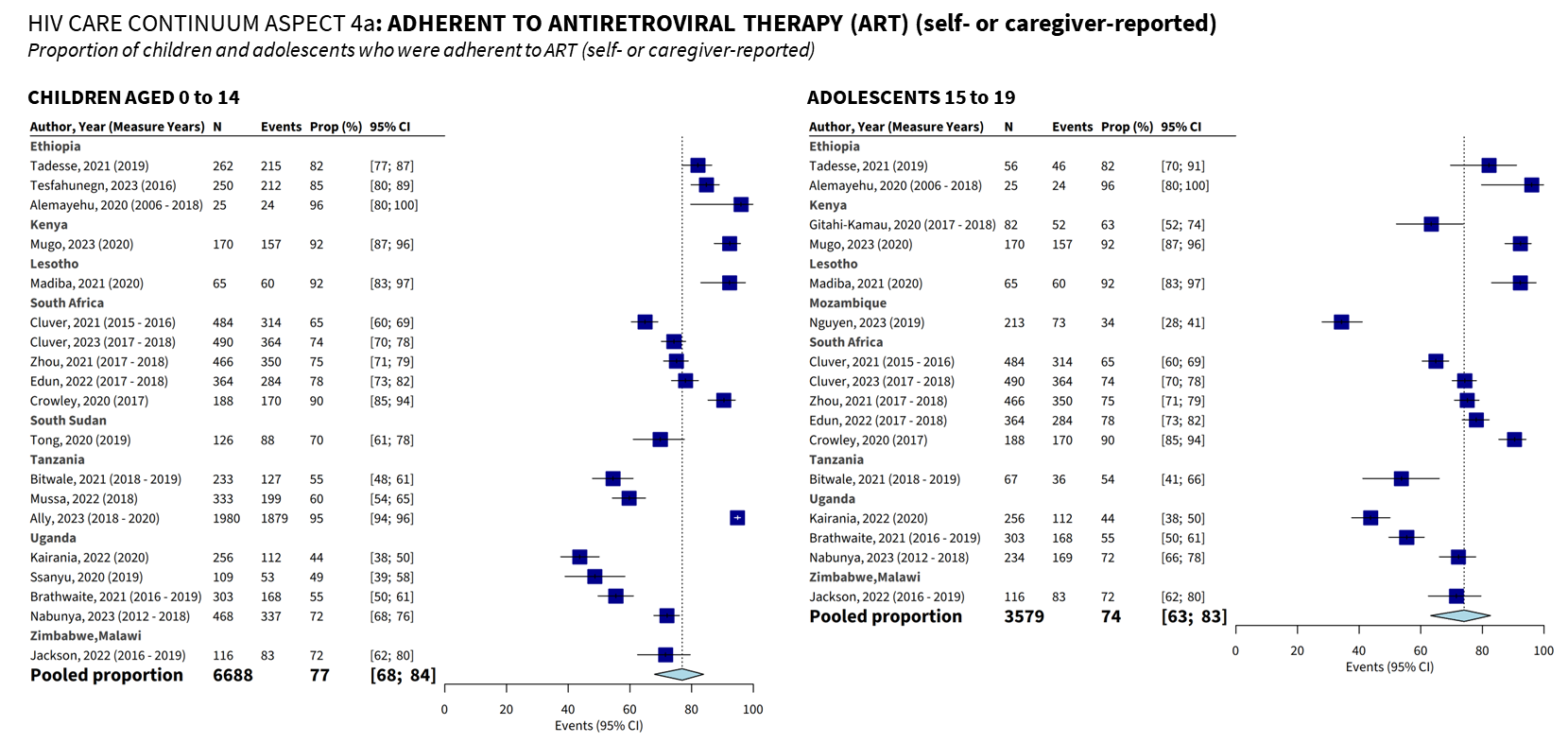


# Supplementary Figure 7. Forest plots of adherent to antiretroviral therapy (self- or caregiver-reported), by children 0-14 and adolescents 15-19, in Eastern and Southern Africa.


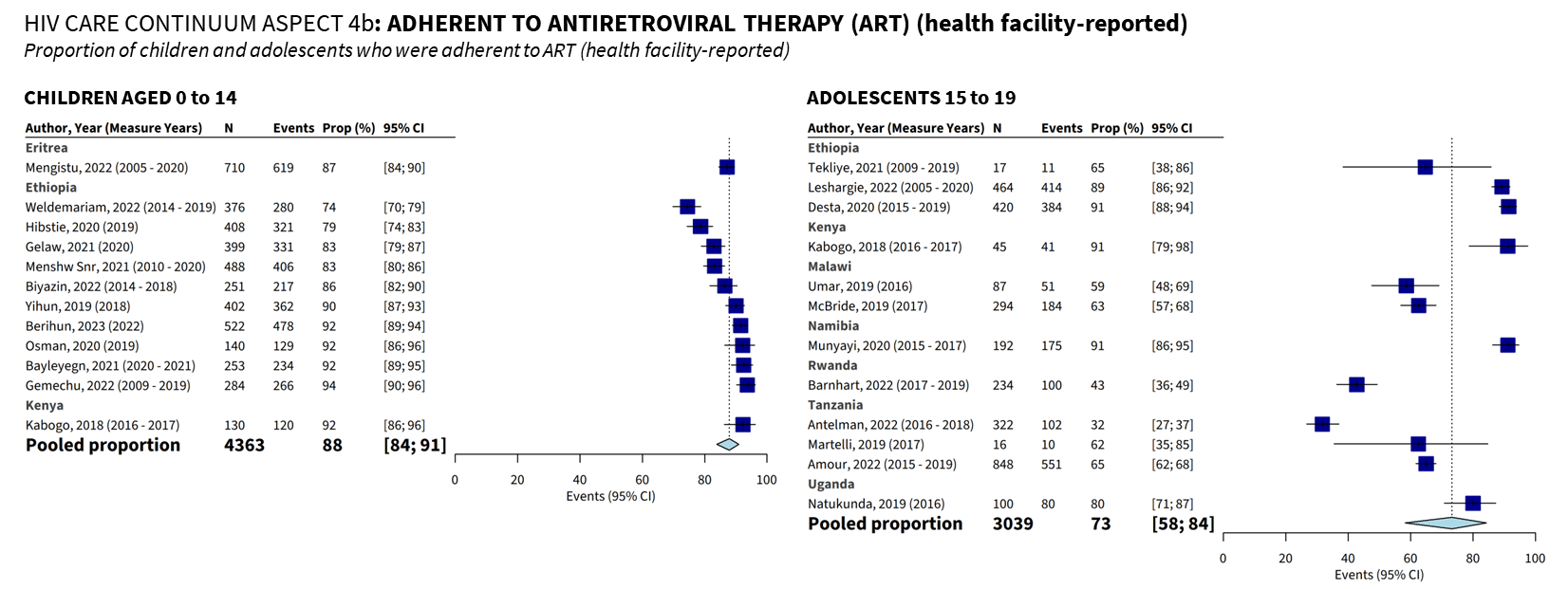


# Supplementary Figure 8. Forest plots of adherent to antiretroviral therapy (health facility-reported), by children 0-14 and adolescents 15-19, in Eastern and Southern Africa.


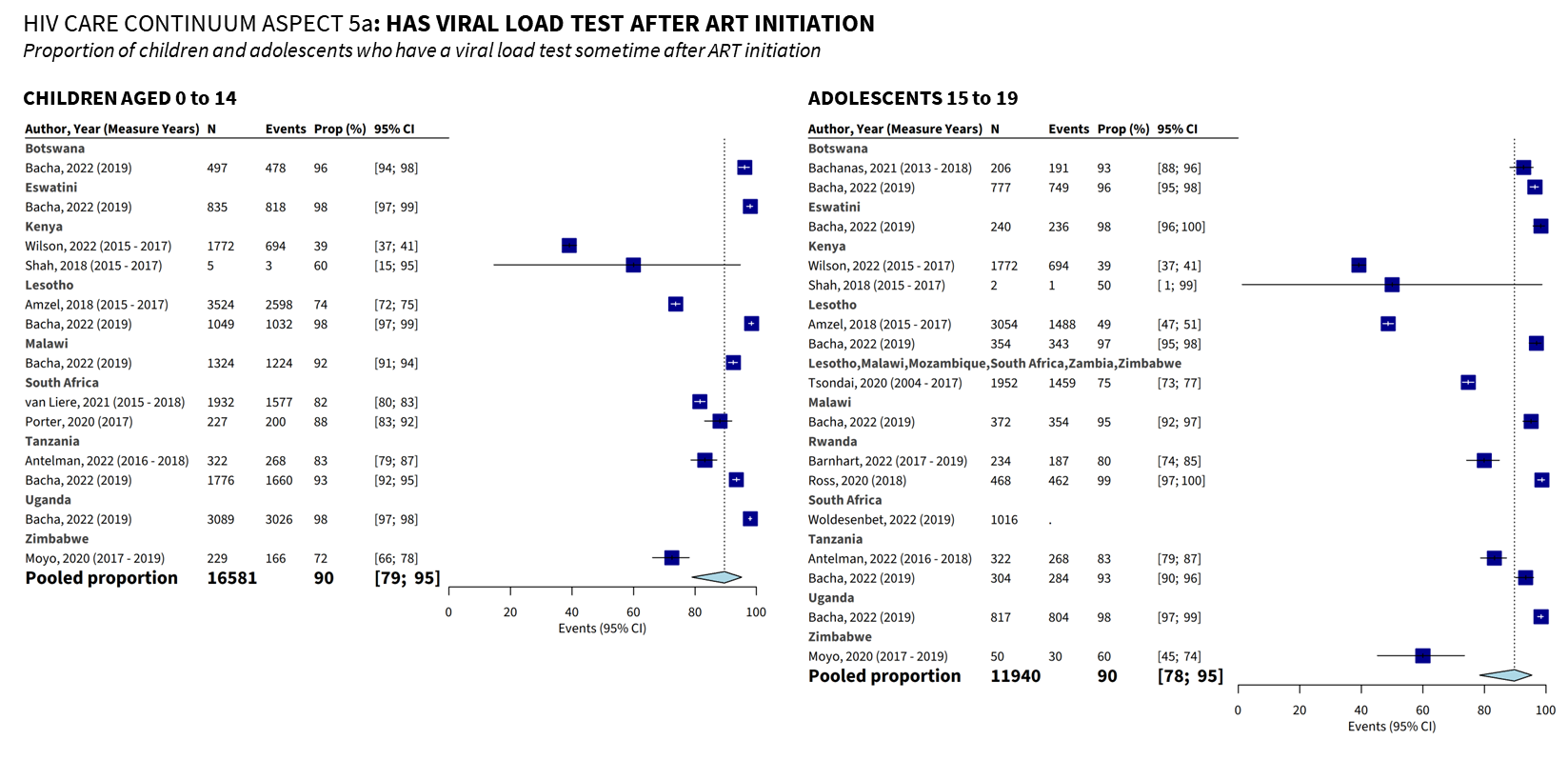


# Supplementary Figure 9. Forest plots of having a viral load test after ART initiation, by children 0-14 and adolescents 15-19, in Eastern and Southern Africa.


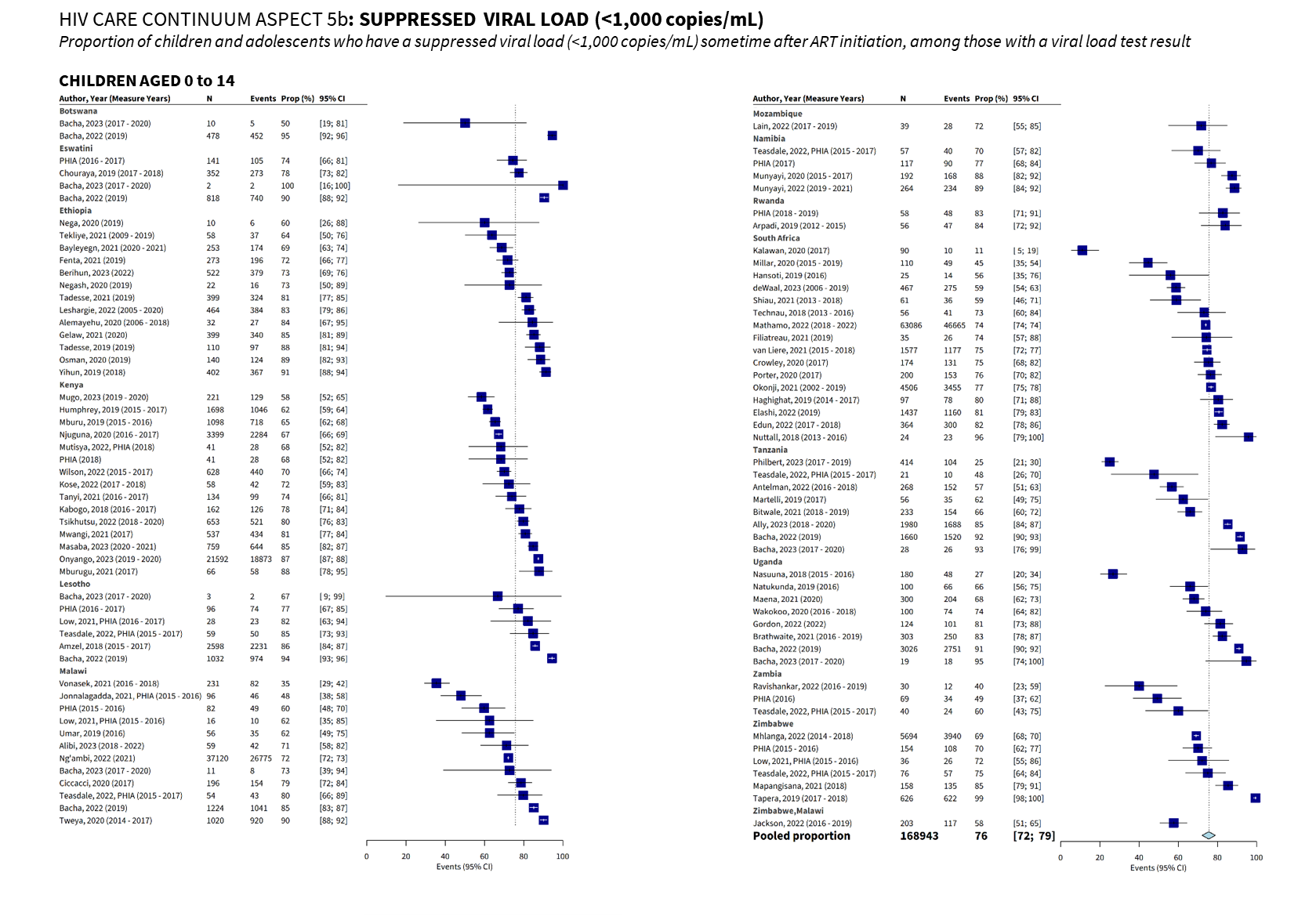


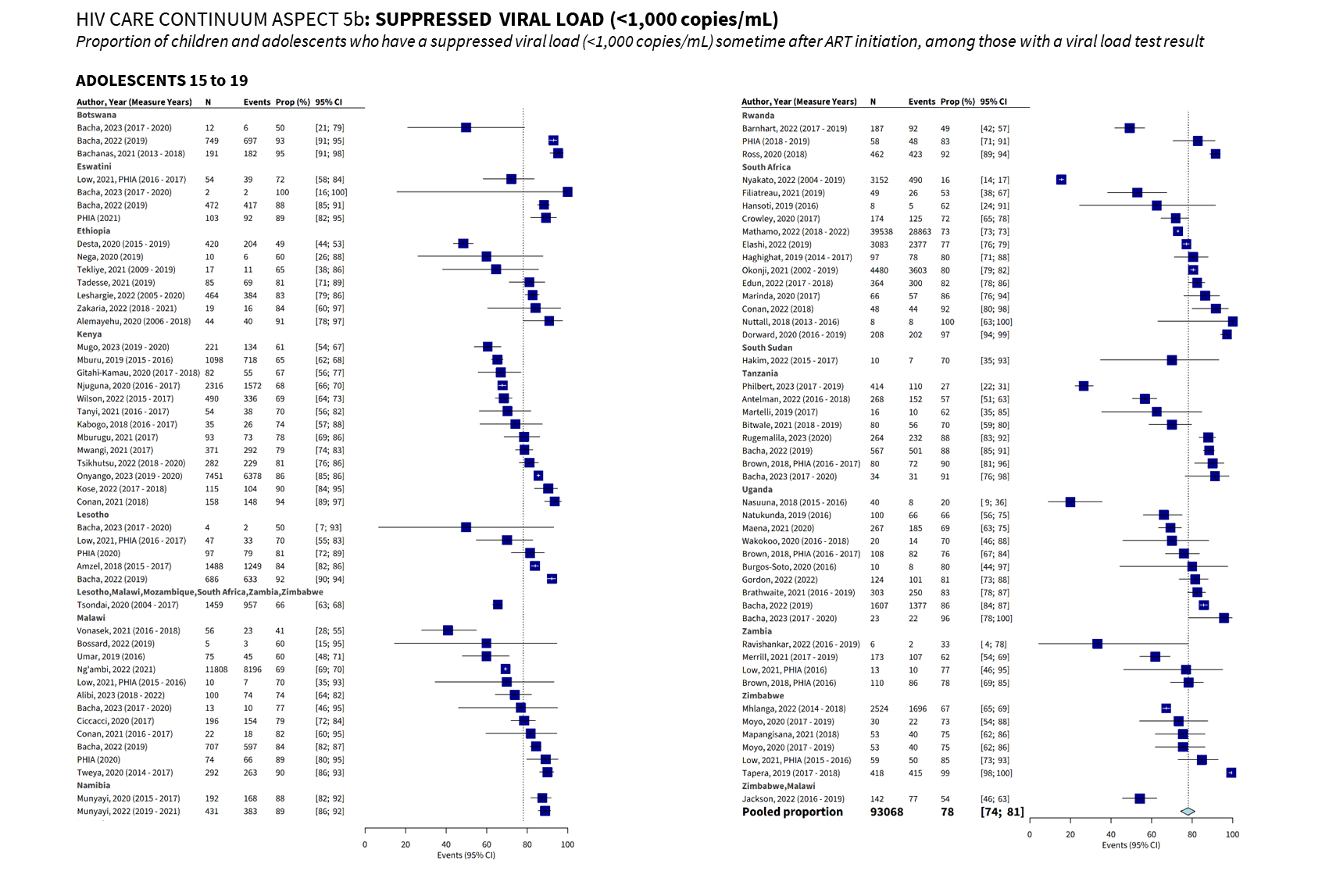


# Supplementary Figure 10. Forest plots of having a suppressed viral load (<1,000 copies/ml), by children 0-14 and adolescents 15-19, in Eastern and Southern Africa.
